# Integrative open workflow for confident annotation and molecular networking of metabolomics MS^E^/DIA data

**DOI:** 10.1093/bib/bbae013

**Published:** 2024-02-07

**Authors:** Albert Katchborian-Neto, Matheus F Alves, Paula C P Bueno, Karen de Jesus Nicácio, Miller S Ferreira, Tiago B Oliveira, Henrique Barbosa, Michael Murgu, Ana C C de Paula Ladvocat, Danielle F Dias, Marisi G Soares, João H G Lago, Daniela A Chagas-Paula

**Affiliations:** Chemistry Institute, Federal University of Alfenas, 37130-001, Alfenas, Minas Gerais, Brazil; Chemistry Institute, Federal University of Alfenas, 37130-001, Alfenas, Minas Gerais, Brazil; Chemistry Institute, Federal University of Alfenas, 37130-001, Alfenas, Minas Gerais, Brazil; Leibniz Institute of Vegetable and Ornamental Crops (IGZ), Theodor-Echtermeyer-Weg 1, 14979, Großbeeren, Germany; Department of Chemistry, Federal University of Mato Grosso, 14040-901, Cuiabá, Mato Grosso, Brazil; Chemistry Institute, Federal University of Alfenas, 37130-001, Alfenas, Minas Gerais, Brazil; Department of Pharmacy, Federal University of Sergipe, 49100-000, São Cristóvão, Sergipe, Brazil; Center of Natural Sciences and Humanities, Federal University of ABC, 09210-180, Santo Andre, São Paulo, Brazil; Waters Corporation, Alameda Tocantins 125, Alphaville, 06455-020, São Paulo, São Paulo, Brazil; Department of Pharmaceutical Sciences, Federal University of Juiz de Fora, 36036-900, Juiz de Fora, Minas Gerais, Brazil; Chemistry Institute, Federal University of Alfenas, 37130-001, Alfenas, Minas Gerais, Brazil; Chemistry Institute, Federal University of Alfenas, 37130-001, Alfenas, Minas Gerais, Brazil; Center of Natural Sciences and Humanities, Federal University of ABC, 09210-180, Santo Andre, São Paulo, Brazil; Chemistry Institute, Federal University of Alfenas, 37130-001, Alfenas, Minas Gerais, Brazil

**Keywords:** chemical annotation, data-independent acquisition, open software, Ocotea, mass spectrometry

## Abstract

Liquid chromatography coupled with high-resolution mass spectrometry data-independent acquisition (LC-HRMS/DIA), including MS^E^, enable comprehensive metabolomics analyses though they pose challenges for data processing with automatic annotation and molecular networking (MN) implementation. This motivated the present proposal, in which we introduce DIA-IntOpenStream, a new integrated workflow combining open-source software to streamline MS^E^ data handling. It provides ‘in-house’ custom database construction, allows the conversion of raw MS^E^ data to a universal format (.mzML) and leverages open software (MZmine 3 and MS-DIAL) all advantages for confident annotation and effective MN data interpretation. This pipeline significantly enhances the accessibility, reliability and reproducibility of complex MS^E^/DIA studies, overcoming previous limitations of proprietary software and non-universal MS data formats that restricted integrative analysis. We demonstrate the utility of DIA-IntOpenStream with two independent datasets: dataset 1 consists of new data from 60 plant extracts from the *Ocotea* genus; dataset 2 is a publicly available actinobacterial extract spiked with authentic standard for detailed comparative analysis with existing methods. This user-friendly pipeline enables broader adoption of cutting-edge MS tools and provides value to the scientific community. Overall, it holds promise for speeding up metabolite discoveries toward a more collaborative and open environment for research.

## INTRODUCTION

Classical purification/isolation procedures for chemical characterization in the field of natural products (NPs) are known for their laborious nature, involving multiple chromatographic steps and frequently afford well-known compounds. To solve this problem, more recently, chemical annotation using liquid chromatography coupled with high-resolution mass spectrometry (LC-HRMS) has become the gold standard in the pursuit of a more rapid and efficient metabolite content assessment for either known compounds, as well as the isolation of the unknown ones [1–4].

Data-independent acquisition (DIA) is a mass spectrometry (MS) acquisition mode that systematically fragments precursor ions within a specific mass-to-charge ratio (*m/z*) range. It has the advantage of detecting low-abundance metabolites, which are often overlooked by conventional data-dependent acquisition (DDA) methods, due to their unavoidable loss of MS data coverage [5, 6]. MS^E^, developed by Waters™ for Quadrupole Time of Flight (Q-TOF) MS analyzers, is a DIA method that fragments all precursor ions within the entire acquisition window by alternating between low- and high-collision energies, thereby obtaining consecutive scans of precursors and their fragments. This unbiased tandem MS approach is therefore considered DIA due to its unbiased fragmentation of precursors, irrespective of their abundance [7, 8]. The terms MS^all^ and all-ion fragmentation (AIF) have been also employed for a similar type of fragmentation with Orbitrap analyzer-based instruments from Thermo Fisher™ [5, 7, 9].

Despite the advancement in MS techniques, challenges persist, especially in software availability for processing data obtained through DIA methods. While there are robust options for proprietary-specific software, e.g. UNIFI (Waters™), open software options are limited. In this scenario, the MS-DIAL is an extensively used option for users of a wide variety of mass spectrometers. Other more recent approaches for processing and annotation include DIAMetAlyzer, DecoID and MetaboMSDIA, each of which has particular advantages and limitations [10–13].

In molecular networking (MN), each processed mass spectrum is represented as a node, and spectral similarities between nodes can be calculated using different algorithms such as the cosine similarity of the Global Natural Product Social Molecular Networking (GNPS) [14, 15]. Despite its potential, processing only DIA-MS data for automated annotation and MNs in metabolomics remains challenging compared to the well-established DDA workflows [14, 16]. Therefore, these challenges have motivated the DIA-IntOpenStream pipeline to be built. The present study brings novelty by offering a comprehensive pipeline for processing LC-HRMS-DIA/MS^E^ data that automates the generation of custom databases using free commercial software. We additionally showcased the pipeline’s advantages with the successful utilization of the universal .mzML MS data format to process, annotate and generate functional MN. Finally, we focus on the current challenges in LC-HRMS-DIA/MS^E^ data analysis, offering strategies to mitigate these drawbacks and providing critical insights for future advancements.

This integrative approach enhances confidence in the annotation of known compounds and facilitates the discovery of novel and/or structurally related compounds. Therefore, it also enables the prioritization of unknown metabolites of interest for further investigation. Our study validates the DIA-IntOpenStream pipeline with two independent datasets. Dataset 1 consists of LC-HRMS/DIA data from 60 *Ocotea* plant extracts, showcasing the pipeline’s applicability in plant metabolomics. Dataset 2 is a publicly available actinobacterial extract dataset enriched with a diverse pool of chemical authentic standards, encompassing a range of antimicrobial and naturally occurring compounds. The inclusion of known standards allows evaluation of the pipeline’s annotation accuracy and efficiency. Thus, this dataset provides a solid foundation for a detailed comparative study with the original well-designed research that has performed the study using non-open software [17]. A key advantage of DIA-IntOpenStream is that it relies exclusively on open-source software. Thus, it is a cost-effective alternative to achieve equivalent and even complementary results to the standard approaches, thereby also enabling high-quality metabolomics analysis based on LC-MS/DIA data.

## RESULTS

### General pipeline workflow

LC-HRMS/DIA techniques such as MS^E^ generate highly complex datasets that require specialized software for processing and annotation. Until recently, MN generation required vendor software or the use of non-universal MS data format (e.g. ABF from MS-DIAL), limiting the execution of integrated and fully MS^E^ data analyses. In contrast, DIA-IntOpenStream uses a standardized MS data format (.mzML) and open software tools for MS^E^ data processing and annotation. The integrated workflow provides enhanced confidence for general, automated annotation strategies and provides an accessible way to increase the reliability of metabolome annotation coverage using DIA data. Indeed, the pipeline is adaptable for any MS^E^ or AIF LC-MS analysis. Step 1 starts with the raw MS^E^ acquisition data. In step 2, MS^E^ raw data are converted into standard .mzML format using a Waters2mzML (https://github.com/AnP311/Waters2mzML), first published to GitHub in late 2022; however it is still under development and limited to Microsoft Windows operating systems. Waters2mzML implements a Python-based wrapper for ProteoWizard msConvert (https://proteowizard.sourceforge.io/), the most used open MS converter software. Step 3 is the Konstanz Information Miner (KNIME) workflow that can be rapidly executed, and the result is the generation of custom ‘in-house’ databases (DBs). These DBs are imported in the following steps 4 (MZmine 3 processing) and 5 (MS-DIAL processing). Step 3 is important for automatic enhanced annotation with level 3 of confidence during the data processing steps, leveraging the quality, processing power and annotation of both MZmine 3 and MS-DIAL 4.9 software [18, 19]. Of note, the generated KNIME database output is exported as a .csv file and subsequently imported into MZmine 3, while a.txt file is used for MS-DIAL. Furthermore, from MS-DIAL, DIA data are exported in .mgf spectra format together with the GNPS feature table (.csv). In step 6, these two files and the additional metadata are submitted using WinSCP remote server software for feature-based molecular networking (FBMN) in the GNPS platform. Step 7 applies FBMN analysis and automated annotation with level 2 of confidence. Step 8 consists of semi-automated strategies in Cytoscape software that are employed for data, inspection, visualization and integration of FBMN and in-house DB annotations.

**Figure 1:**
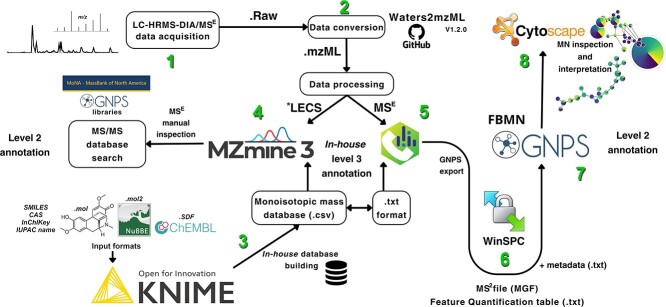
Schematic representation of the integrated process for generating an ‘in-house’ automatic database and performing LC-HRMS/DIA data processing to create molecular networking. After data acquisition (step 1), LC-HRMS/DIA data are converted to the standardized .mzML universal format using Waters2mzML V1.2.0 (step 2). Custom ‘in-house’ DB specific to the research is automatically prepared in the KNIME platform (step 3) using drawn chemical structures or can be downloaded from online libraries in various standard formats (.mol, .mol2, .sdf). Table formats (.csv) containing SMILES, CAS number, InchKey, or IUPAC chemical name can also be utilized. The converted .mzML data are then imported into MZmine 3 (step 4) and MS-DIAL (step 5) for processing, where the output ‘in-house’ DB generated in KNIME is integrated to enable automatic annotation (level 3 of confidence). MS-DIAL exports the align results as a GNPS feature table (.txt) and MS2 file (.mgf) that along with the custom metadata (.txt) are submitted via WinSCP remote server software to the GNPS environment (step 6). Feature-Based Molecular Networking (FBMN) analysis with automated level 2 of confidence annotation is performed on GNPS (step 7). Semi-automated strategies in Cytoscape software are employed for the data visualization and integration of FBMN and in-house DB annotations at levels 2 and 3 of confidence, respectively (step 8). ^*^Low energy channel scans (LECS)/MS^1^.

The integration of the processed data with online MS spectral libraries allowed for the automated annotation of metabolites. Supplementation with data gathered from customized ‘in-house’ annotations has bolstered confidence in the annotation of the molecular families generated. The construction of tailored in-house DBs with metabolites of interest is critical to increase annotation reliability, as it provides matches with metabolites specific to a given taxon under investigation. For example, the utilization of the *Ocotea*DB and *Actinomarine*DB built with KNIME dramatically enhanced the reliability of our metabolite annotations by reducing the likelihood of potential false positives, thereby increasing true hits. Overall, strategic integration of automatic custom, automated ‘in-house’ annotations with online libraries and optimized GNPS parameters described in this pipeline enables MN with robust metabolite annotation of DIA/MS^E^ data, as schematically demonstrated in Figure 1.

### In-house database and KNIME workflow

**Figure 2:**
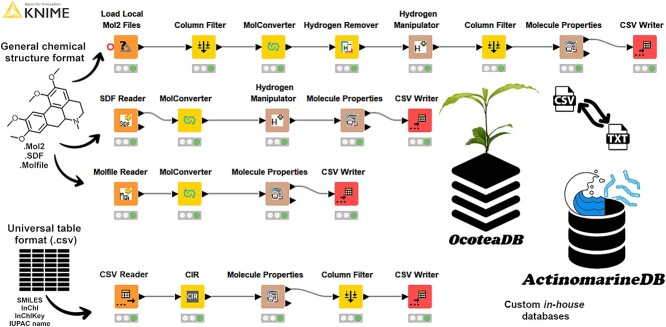
KNIME workflow for high confidence in-house automatic database assembly. The workflow includes .mol, .mol2 and .sdf chemical structures as input data for the respective node readers. The last node is a .csv table reader that accepts tables containing either SMILES, CAS numbers, InChIKey or IUPAC names as input data. The final output is a .csv (or .txt) file with three columns: chemical name, calculated molecular formula and calculated monoisotopic mass. The in-house DB specific for the aiming samples can now be integrated into the data processing step to increase confidence in the metabolite annotation.

In general, this workflow accepts the four most common types of chemical input data, namely, .mol, .mol2, .sdf and .csv. The table input files could be formatted as Simplified Molecular Input Line Entry System (SMILES), International Chemical Identifier (InChIKey), Chemical Abstracts Service (CAS) number or International Union of Pure and Applied Chemistry (IUPAC) names. The output is a .csv file with three columns: chemical structure name, calculated molecular formula and calculated monoisotopic mass. To generate the *Ocotea*DB dataset, the KNIME workflow was run with 492 molecular structures from *Ocotea* spp. in .mol format, drawn from online databases. The total runtime of the workflow was 84 s (see Methods section for desktop configuration check) and was used for later ‘in-house’ annotation during data processing. The *Actinomarine*DB dataset was generated with 6481 NPs sourced from the online npatlas database (https://www.npatlas.org/) in .csv format and comprised of the genera of *Actinomyces*, *Streptomyces*, *Salinospora*, *Micromonospora*, *Nocardia*, *Actinomadura* and *Rhodococcus*, running for 63 s. The *Ocotea*DB and the *Actinomarine*DB .csv files were successively uploaded into MZmine 3 for annotation. Additionally, .txt export versions can be imported into MS-DIAL for annotation. Figure 2 illustrates the reader, converter and writer nodes.

### LC-HRMS data conversion and processing

Dataset 1 along with quality controls (QCs) and blanks in the Waters™ .raw format was effectively converted with Waters2mzML to generate functional centroided .mzML files. The same step was performed for dataset 2. These conversions took ~36 and 1.5 h, respectively, on our computer configuration (detailed in the Methods section). The files were then processed using the MZmine 3 and MS-DIAL 4.9. For MZmine 3, despite the large cohort of dataset 1, final batch processing required only ~7 min per ionization mode, while dataset 2 took only 1 min. While actual processing is quite fast (a few minutes), software parameter optimization is time-demanding, although empowers robust data processing for complex samples. Dataset 1 yielded 18 805 aligned features in the positive mode, including 3983 annotation hits from *Ocotea*DB with all potential adducts identified. Similarly, the negative mode yielded 23 304 features with 3216 database annotation hits.

In contrast, the positive mode analysis with MS-DIAL 4.9 required ~2.11 h for dataset 1, resulting in the annotation of 22 572 features, while the analysis from the negative mode analysis took around 1.58 h, yielding 21 838 features. For dataset 2, MS-DIAL 4.9 processing required only 4 min. The dataset acquisition parameters and data size have a great influence on processing time, especially aligning an elevated number of samples, as in the case of dataset 1. In addition, despite inherent variations in parameters and algorithms employed by the two programs, the results generated were comparable. More specifically, MS-DIAL exhibited a longer processing time as it can appropriately process and assign MS^2^ fragment ions to MS^1^ precursor ions in DIA data. This step is the slowest during data processing and is particularly mandatory for MN implementation. Details of the dataset 2 processing results are provided at SM-4 and 5.

Although DIA processing algorithms are present in MZmine 3, they were not employed in this pipeline as they remain in an experimental phase without publicly available guides or tutorials to standardize parameter values, different from DDA data processing, which is very well established. As such, we used MZmine only to perform MS^1^ data processing, which explains the increased speed of data processing results when compared to MS-DIAL. Nevertheless, despite this limitation, we highlight that the MZmine 3 demonstrates remarkable transparency and guidance in data processing and annotation. Further insights and considerations are further provided in the Discussion section.

### Chemical annotation

**Figure 3:**
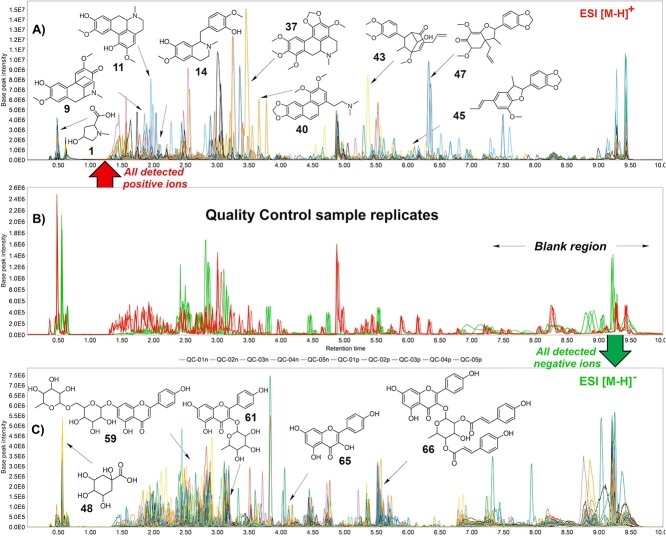
LC-HRMS-DIA/MS^E^ metabolic fingerprints displayed as base peak ions (BPIs) showing the overlapped QC replicates (pool of 60 *Ocotea* spp. extracts) and overlapped individual chromatograms. (**A**) The overlapped metabolic fingerprint of *Ocotea* species in positive ionization mode, including the amino acid derivative 4-hydroxy-*N*-methylproline (1), the alkaloids, flavinantine (9), isoboldine (11), leucoxylonine (37), thalicthuberine (40), as well as the lignoids ocophyllol B (43), licarin B (45) and armenin B (47). (**B**) Pooled, overlapped QC samples, highlighting the blank region. Positive mode is displayed in red and negative in green. Peaks that are found in common among blanks are located after 7 min. (**C**) The overlapped metabolic fingerprint of *Ocotea* species in negative ionization mode, and representative annotation of some compounds, such as quinic acid (48) and the flavonoids apigenin-7-*O*-rutinoside (59), afzelin (61) and kaempferol 3-(2″,4″-di-(*E*)-*p*-coumaryl-rhamnoside (66).

Following established guidelines for untargeted metabolomics, QC samples were prepared for dataset 1. After data analyses, consistent peak distributions and reproducible metabolic fingerprints across the QC injections were observed (Figures 3 and S1). Using QC samples to ensure that predefined quality thresholds are met is a critical step, thereby validating that the analytical system can acquire high-quality metabolomics data from the experimental samples [20, 21]. The developed LC-HRMS/MS^E^ method effectively separated and detected major and minor metabolite components in *Ocotea* spp. The comprehensive list of annotations with level 2 of confidence for the acquired metabolic fingerprint is shown in Table 1. Even with the complexity of our metabolomics data, careful and rigorous data processing led to reliable and clear reproducible results, as shown by the superimposed chromatograms of experimental replicates (Figure S2). The superimposed chromatograms of extract samples and QCs from both positive and negative modes are illustrated in Figures 3 and S3–S5, evidencing the high chemical complexity of data. Details of dataset 2 are provided in Figures S16**–**S18 and Tables S3 and S4.

**Table 1:** ESI-MS^E^ positive and negative modes including annotation with level 2 of confidence of *Ocotea* metabolites from based on the in-house database. This table includes 41 alkaloids (pyrrolidine, proaporphine, noraporphine, aporphine, benzylisoquinoline, morphinandienone and protoberberine subclasses), 6 lignoids (1 lignan and 5 neolignans), 18 flavonoids (glycosylated quercetin, kaempferol and apigenin derivative subclasses) and a cyclic polyol. The last two columns indicate the MS^2^ spectral source used for manual annotation and if spectra were matched automatically on the GNPS platform, respectively

ID	*R*_t_ (min)	Putative metabolite	Metabolite class	MF	*Ocotea* DB	Observed *m/z*	Adduct	Error (ppm)	Spectral references	Automatic GNPS annotation
1	0.49	4-Hydroxy-*N*-methylproline	Pyrrolidine alkaloid	C_6_H_11_NO_3_	Yes	146.08119	[M + H]^+^	0.14	Proposed	No
2	1.45	Crotsparine	Proaporphine alkaloid	C_17_H_17_NO_3_	Yes	284.12770	[M + H]^+^	−1.48	GNPS	Yes
3	1.48	Glaziovine	Proaporphine alkaloid	C_18_H_19_NO_3_	Yes	298.14359	[M + H]^+^	−0.60	GNPS	No
4	1.52	*N*-Methylcoclaurine	Benzylisoquinoline alkaloid	C_18_H_21_NO_3_	Yes	300.15932	[M + H]^+^	−0.33	GNPS	No
5	1.60	3-Hydroxynornuciferine	Noraporphine alkaloid	C_18_H_19_NO_3_	Yes	298.14355	[M + H]^+^	−0.74	GNPS	Yes
6	1.61	Laurelliptine	Noraporphine alkaloid	C_18_H_19_NO_4_	Yes	314.13850	[M + H]^+^	−0.57	Proposed	No
7	1.74	Laurolitsine	Noraporphine alkaloid	C_18_H_19_NO_4_	Yes	314.13853	[M + H]^+^	−0.48	GNPS	No
8	1.63	Pallidine	Morphinandienone alkaloid	C_19_H_21_NO_4_	Yes	328.15444	[M + H]^+^	0.34	GNPS	No
9	1.81	Flavinantine	Morphinandienone alkaloid	C_19_H_21_NO_4_	Yes	328.15448	[M + H]^+^	0.46	Proposed	No
10	1.87	Boldine	Aporphine alkaloid	C_19_H_21_NO_4_	Yes	328.15447	[M + H]^+^	0.43	GNPS	No
11	1.96	Isoboldine	Aporphine alkaloid	C_19_H_21_NO_4_	Yes	328.15440	[M + H]^+^	0.21	GNPS	Yes
12	2.05	Corytuberine	Aporphine alkaloid	C_19_H_21_NO_4_	Yes	328.15438	[M + H]^+^	0.15	GNPS	No
13	2.00	Lauroscholtzine	Aporphine alkaloid	C_20_H_23_NO_4_	Yes	342.17014	[M + H]^+^	0.47	GNPS	Yes
14	2.07	Reticuline	Benzylisoquinoline alkaloid	C_19_H_23_NO_4_	Yes	330.16918	[M + H]^+^	−2.42	MoNA	Yes
15	2.19	Armepavine	Benzylisoquinoline alkaloid	C_19_H_23_NO_3_	Yes	314.17505	[M + H]^+^	−0.06	GNPS	No
16	1.93	Zenkerine	Noraporphine alkaloid	C_18_H_19_NO_3_	Yes	298.14367	[M + H]^+^	−0.34	GNPS	No
17	2.08	Tuduranine	Noraporphine alkaloid	C_18_H_19_NO_3_	No	298.14343	[M + H]^+^	−1.14	Proposed	No
18	2.08	Diospirifoline	Aporphine alkaloid	C_19_H_19_NO_4_	Yes	326.13859	[M + H]^+^	−0.28	Proposed	No
19	2.27	Thaliporphine	Aporphine alkaloid	C_20_H_23_NO_4_	Yes	342.16985	[M + H]^+^	−0.38	GNPS	No
20	2.43	Predicentrine	Aporphine alkaloid	C_20_H_23_NO_4_	Yes	342.17007	[M + H]^+^	0.26	MoNA	No
21	2.47	Nuciferine	Aporphine alkaloid	C_19_H_21_NO_2_	No	296.16469	[M + H]^+^	0.61	GNPS	No
22	2.54	Corydine	Aporphine alkaloid	C_20_H_23_NO_4_	Yes	342.17042	[M + H]^+^	1.29	GNPS	Yes
23	2.50	Domesticine	Aporphine alkaloid	C_19_H_19_NO_4_	Yes	326.13834	[M + H]^+^	−1.04	GNPS	No
24	2.98	Dehydrodicentrine	Aporphine alkaloid	C_20_H_21_NO_4_	Yes	338.13891	[M + H]^+^	0.68	Proposed	No
25	2.46	Norisocorydine	Noraporphine alkaloid	C_19_H_21_NO_4_	Yes	328.15421	[M + H]^+^	−0.37	GNPS	No
26	2.56	Laurotetanine	Noraporphine alkaloid	C_19_H_21_NO_4_	Yes	328.15429	[M + H]^+^	−0.12	GNPS	No
27	2.79	Nordicentrine	Noraporphine alkaloid	C_19_H_19_NO_4_	Yes	326.13862	[M + H]^+^	−0.18	GNPS	No
28	3.01	Nornantenine	Noraporphine alkaloid	C_19_H_19_NO_4_	Yes	326.13864	[M + H]^+^	−0.12	GNPS	Yes
29	3.08	Nornuciferine	Noraporphine alkaloid	C_18_H_19_NO_2_	Yes	282.14870	[M + H]^+^	−0.57	GNPS	No
30	2.69	Lirinidine	Aporphine alkaloid	C_18_H_19_NO_2_	Yes	282.14850	[M + H]^+^	−1.28	GNPS	Yes
31	2.92	Glaucine	Aporphine alkaloid	C_21_H_25_NO_4_	Yes	356.18513	[M + H]^+^	−1.40	GNPS	Yes
32	3.07	Roemerine	Aporphine alkaloid	C_18_H_17_NO_2_	Yes	280.13274	[M + H]^+^	−1.68	GNPS	Yes
33	2.87	Nantenine	Aporphine alkaloid	C_20_H_21_NO_4_	Yes	340.15406	[M + H]^+^	−0.79	MoNA	No
34	3.07	Dicentrine	Aporphine alkaloid	C_20_H_21_NO_4_	Yes	340.15426	[M + H]^+^	−0.21	GNPS	No
35	3.15	Dehydronuciferine	Aporphine alkaloid	C_19_H_19_NO_2_	No	294.14903	[M + H]^+^	0.58	Proposed	No
36	3.31	Dicentrinone	Oxoaporphine alkaloid	C_19_H_13_NO_5_	Yes	336.08626	[M + H]^+^	−0.86	GNPS	No
37	3.52	Leucoxylonine	Aporphine alkaloid	C_22_H_25_NO_6_	Yes	400.17563	[M + H]^+^	0.42	Proposed	No
38	3.05	Stephenanthrine	Phenanthrene alkaloid	C_19_H_19_NO_2_	No	294.14881	[M + H]^+^	−0.17	Proposed	No
39	3.19	Argentinine	Phenanthrene alkaloid	C_19_H_21_NO_2_	Yes	296.16515	[M + H]^+^	2.16	GNPS	Yes
40	3.66	Thalicthuberine	Phenanthrene alkaloid	C_21_H_23_NO_4_	Yes	354.16966	[M + H]^+^	−0.90	GNPS	No
41	3.86	Discretamine	Protoberberine alkaloids	C_19_H_21_NO_4_	Yes	328.15342	[M + H]^+^	−2.77	GNPS	No
42	3.27	Sesamin	Lignan	C_20_H_18_O_6_	Yes	355.11784	[M + H]^+^	0.65	MoNA	No
43	5.38	Ocophyllol B	Neolignan	C_21_H_26_O_5_	Yes	359.18565	[M + H]^+^	0.97	Proposed	No
44	5.45	Eusiderin	Neolignan	C_22_H_26_O_6_	Yes	387.17956	[M + H]^+^	−1.70	Proposed	No
45	5.99	Licarin B	Neolignan	C_20_H_20_O_4_	Yes	325.14306	[M + H]^+^	−1.17	Proposed	No
46	6.30	Licarin A	Neolignan	C_20_H_22_O_4_	Yes	327.15865	[M + H]^+^	−1.34	MoNA	No
47	6.34	Armenin B	Neolignan	C_21_H_24_O_6_	Yes	373.16390	[M + H]^+^	−1.77	Proposed	No
48	0.56	Quinic acid	Cyclic polyol	C_7_H_12_O_6_	Yes	191.05433	[M-H]^−^	−9.32	MoNA	No
49	1.75	Taxifolin	Flavanonol	C_15_H_12_O_7_	Yes	303.04983	[M-H]^−^	−3.96	GNPS	No
50	2.07	Catechin/ Epicatechin	Flavonol	C_15_H_14_O_6_	Yes	289.07091	[M-H]^−^	−2.94	MoNA	No
51	2.40	Isoquercitrin	Glycosylated flavone	C_21_H_20_O_12_	Yes	463.08689	[M-H]^−^	−2.83	MoNA	Yes
52	2.42	Vitexin-2’-*O*-rhamnoside	Glycosylated flavone	C_27_H_30_O_14_	Yes	577.15701	[M-H]^−^	1.26	GNPS	No
53	2.46	Rutin	Glycosylated flavone	C_27_H_30_O_16_	No	609.14699	[M-H]^−^	1.44	GNPS	Yes
54	2.54	Quercimeritrin	Glycosylated flavone	C_21_H_20_O_12_	Yes	463.08762	[M-H]^−^	−1.25	Proposed	No
55	2.54	Vitexin	Glycosylated flavone	C_21_H_20_O_10_	Yes	431.09731	[M-H]^−^	−2.46	GNPS	No
56	2.66	Quercitrin	Glycosylated flavone	C_21_H_20_O_11_	Yes	447.09299	[M-H]^−^	−0.67	GNPS	Yes
57	2.73	Reynoutrin	Glycosylated flavone	C_20_H_18_O_11_	Yes	433.07686	[M-H]^−^	−1.71	MoNA	Yes
58	2.74	Astragalin	Glycosylated flavone	C_21_H_20_O_11_	Yes	447.09298	[M-H]^−^	−0.69	MoNA	No
59	2.74	Apigenin-7-*O*-rutinoside	Glycosylated flavone	C_27_H_30_O_14_	Yes	577.15675	[M-H]^−^	0.81	MoNA	Yes
60	2.85	Schaftoside/ Isoschaftoside	Glycosylated flavone	C_26_H_28_O_14_	Yes	563.14164	[M-H]^−^	1.79	GNPS	Yes
61	3.15	Afzelin	Glycosylated flavone	C_21_H_20_O_10_	Yes	431.09706	[M-H]^−^	−3.04	MoNA	No
62	4.46	Kaempferol 3-4″-*p-*coumaryl-rhamnoside	Glycosylated flavone	C_30_H_26_O_13_	Yes	577.13474	[M-H]^−^	−0.71	Proposed	No
63	3.57	Quercetin	Flavonol	C_15_H_10_O_7_	Yes	301.03286	[M-H]^−^	−8.37	MoNA	No
64	4.04	Apigenin	Glycosylated flavone	C_15_H_10_O_5_	Yes	269.04407	[M-H]^−^	−5.32	GNPS	No
65	4.10	Kaempferol	Flavonol	C_15_H_10_O_6_	Yes	285.03873	[M-H]^−^	−6.07	GNPS	No
66	5.46 / 5.55	Kaempferol 3-(2″,4″- di-(E)-*p-*coumaryl-rhamnoside)/ Kaempferol 3-(3″,4″- di-(E)-*p-*coumaryl-rhamnoside)	Glycosylated flavone	C_39_H_32_O_14_	Yes	723.17282	[M-H]^−^	1.23	GNPS	Yes

The IDs with the respective ion fragments observed on MS^E^ spectra are detailed in Table S1. Chemical structures are provided in Figures S8–S12. Spectral matching was done manually for all IDs using online MS reference spectra libraries (MoNA and GNPS). The proposed fragmentations were based on the literature with diagnostic ions for the annotated metabolite classes [23–27].

For dataset 1, alkaloids, lignoids and flavonoids were the main annotated classes in the *Ocotea* spp. samples (Figure 3). In the positive mode, high-intensity levels were observed for an amino acid derivative 4-hydroxy-*N*-methylproline (**1**, *m/z* 146.0812—*R*_t_ 0.49 min), the morphinan alkaloid isomers of flavinantine (**9**, *m/z* 328.1546—*R*_t_ 1.75 min), the aporphine alkaloid isoboldine (**11**, *m/z* 328.1546—*R*_t_ 1.96 min), also some lignoids, including the ocophyllol B (**43**, *m/z* 359.1856—*R*_t_ 5.38 min) and flavonoids such as apigenin-7-*O*-rutinoside (**61**, *m/z* 577.1569—*R*_t_ 2.75 min) and the kaempferol 3-(2″,4″-di-(*E*)-*p*-coumaryl-rhamnoside) (**66**, *m/z* 723.1728—*R*_t_ 5.46 min). Alkaloids and lignoids were ionized mainly in the positive mode, while flavonoids were annotated in the negative ionization mode.

**Figure 4:**
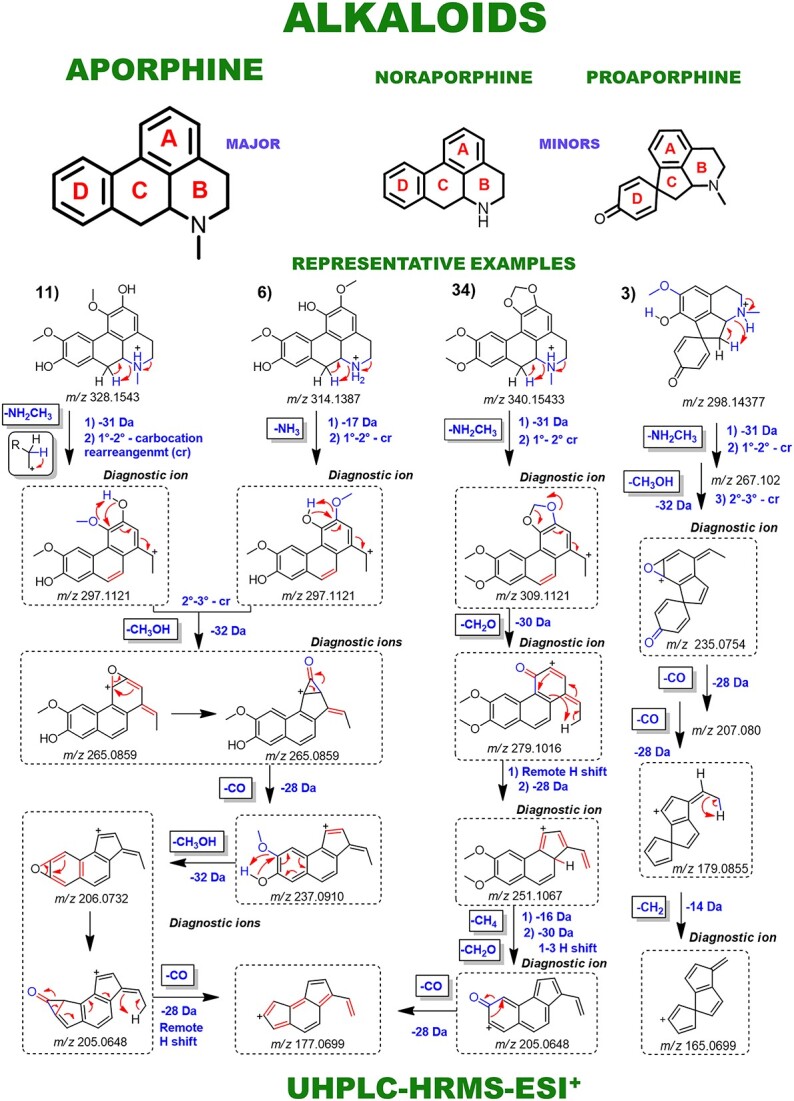
Main fragmentation pattern of proaporphine, noraporphine and aporphine alkaloids, along with their key diagnostic ions. Representative examples are demonstrated: proaporphines: 3—glaziovine. Norapomorphines: 6—laurelliptine and aporphines: 10—boldine.

**Figure 5:**
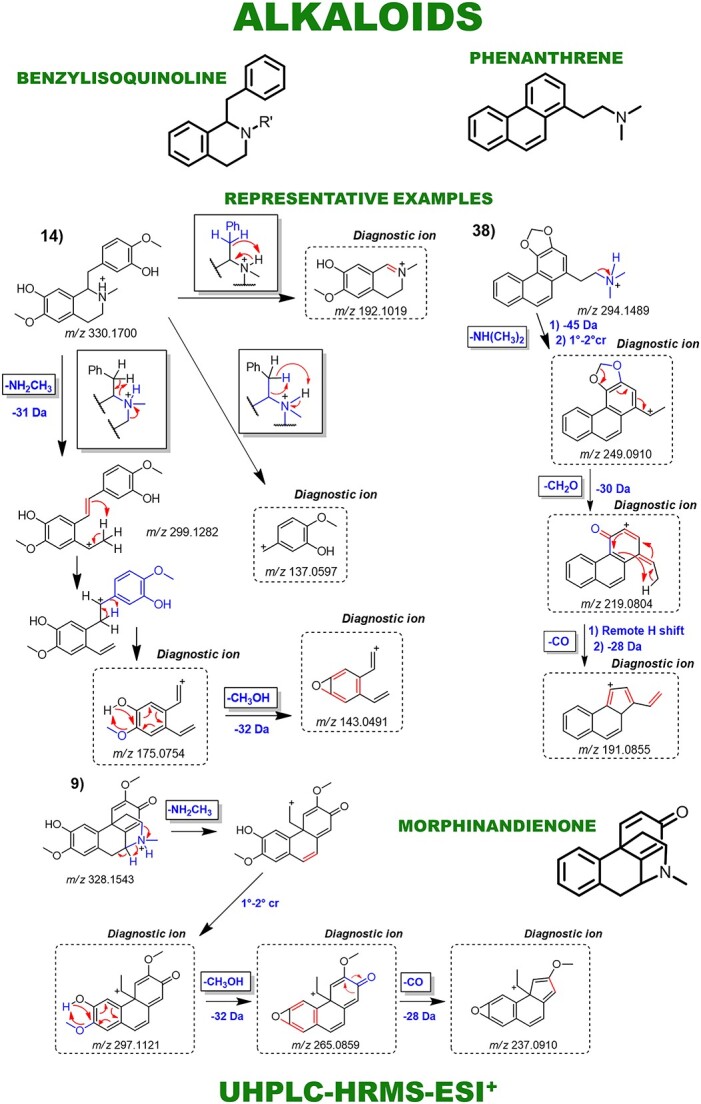
Main fragmentation pattern of benzylisoquinoline, phenanthrene and morphinan alkaloids, along with their key diagnostic ions. Representative examples are demonstrated: Benzylisoquinoline: 14—reticuline. Morphinandienone: 9—flavinantine. Phenanthrene: 38—stephenanthrine.

**Figure 6:**
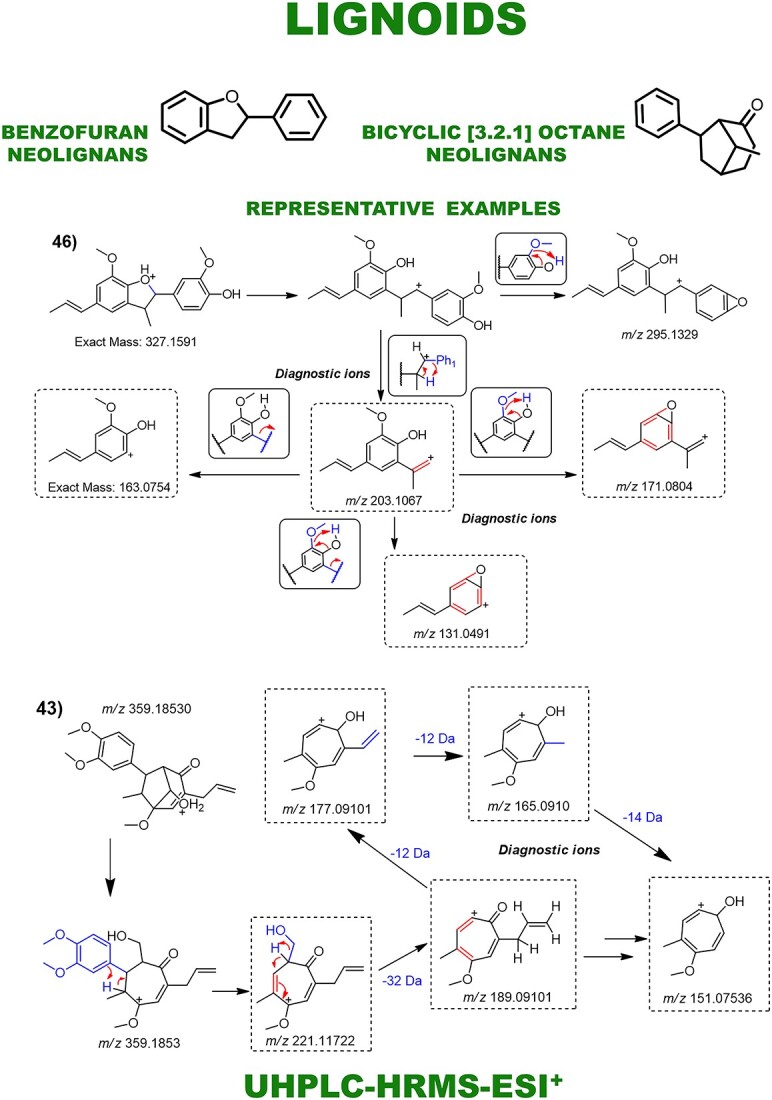
Main fragmentation pattern of lignoids and their key diagnostic ions. Representative examples are demonstrated: Lignoids: 43—ocophyllol B and 46—licarin A.

Aporphines and benzylisoquinoline alkaloids were annotated as major compounds, all of which have known fragmentation patterns and were typically distinguished from each other by neutral losses (Figure 4). Fragmentation patterns of the morphinandienones and phenanthrenes alkaloids, as well as the NP subclasses of lignoids and flavonoids, were also demonstrated (Figures 5 and 6). Automated integration of ‘in-house’ DB by matching MS^1^ monoisotopic masses enabled manual examination of MS^E^ spectra using MZmine 3, leading to level 2 confidence identification of 66 specialized metabolites. No public mass spectral data were available for 15 annotated metabolites, so fragmentation was proposed based on relevant literature and chemical knowledge. Thus, for these metabolites, level 2 of confidence was given based on the classification by Schymanski *et al.* [22]. Diagnostic ion fragments were searched on the Mass Bank of North America (MoNA) and GNPS libraries. In parallel, automated annotation of MS^E^ spectra via MS-DIAL-GNPS-FBMN identified 155 NPs. A comparison between the manual and automated annotations revealed just 18 shared annotations (Table 1). The complete observed product ions and GNPS annotations are detailed in Table S1 and online (https://zenodo.org/records/10383866), respectively.

**Figure 7:**
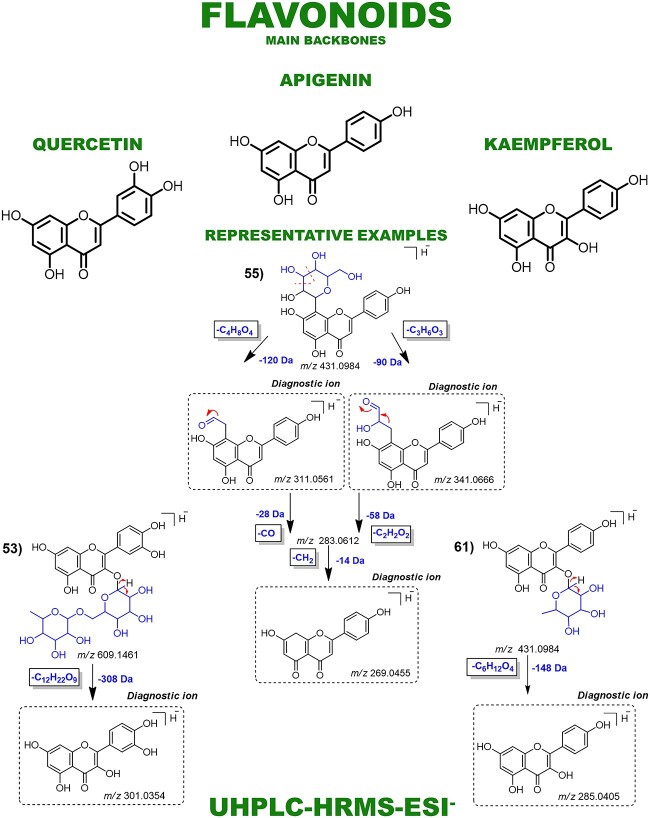
Main fragmentation pattern of flavonoids and their key diagnostic ions. Representative examples are demonstrated: Glycosylated apigenin: 51—vitexin. Glycosylated quercetin: 54—rutin. Glycosylated kaempferol: 64—afzelin.

### Gas-phase fragmentation reactions

The key distinction observed for alkaloids was based on the *N*-substitution pattern. Norapomorphines showed a 14.01 Da mass reduction versus aporphines due to the presence of a radical hydrogen instead of a methyl *N*-substitution. This allowed a distinction between the two alkaloid subclasses. Prevalent neutral losses were 17.03 Da (NH_3_) and 31.01 Da (CH_3_NH_2_) from isoquinoline ring opening (Figure 4). Additionally, losses of CH_3_OH (32.03 Da) occurred due to adjacent hydroxyl and methoxy groups in aporphine rings followed by neutral CO loss (27.99 Da). Fragmentation patterns of some of the less common alkaloids found in *Ocotea* spp., including benzylisoquinolines, morphinandienones and phenanthrenes, are also depicted in Figure 5. This reveals some shared and distinctive fragmentation patterns among the diversity of the annotated alkaloids, as explained in ST-1. Fragmentation proposals were based on chemical knowledge and supported by the literature [23–27].

Flavonoids and lignoids displayed characteristic neutral losses and fragment ions as well (Figure 6). The fragmentation of lignoids was evidenced by neutral losses of methyl (14.01 Da), methoxy (32.03 Da), retro-Diels-Alder reactions and aromatic ring cleavages. These fragmentations formed diagnostic ions that allowed the differentiation of bicyclo neolignans and benzofuran lignoids. For flavonoids, fragmentation predominantly involved glycosidic bond cleavages and losses of saccharide units. These included losses of pentoses (132.04 Da), deoxyhexoses (146.05 Da), hexoses (162.05 Da), glucuronic acids (176.03 Da) and rutinoses (308.09 Da), giving characteristic product ions. These neutral losses provided clues to the types of glycosylation present on the flavonoid scaffolds. Key diagnostic ions for flavonoid aglycones allowed differentiation between subclasses such as apigenin, quercetin and kaempferol. Overall, these characteristic fragmentation patterns allowed differentiation between the main flavonoid subclasses present in *Ocotea* species (ST-1).

### FBMN

**Figure 8:**
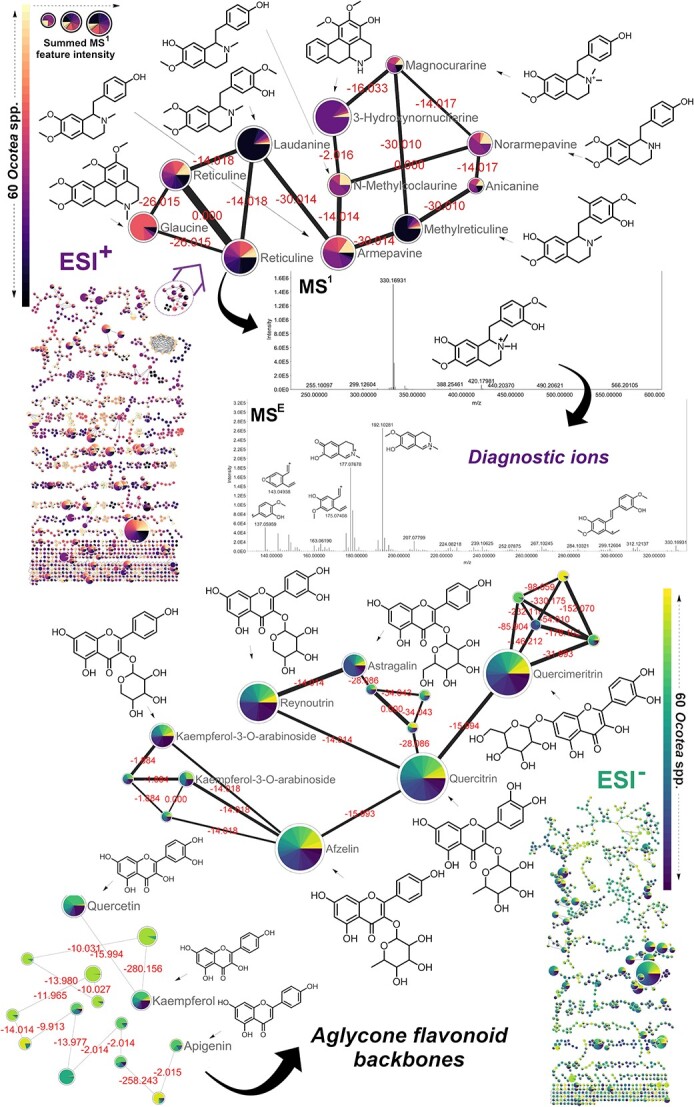
Molecular families of aporphine and benzylisoquinoline alkaloids as well as the glycosylated flavonoid cluster families derived from the FBMN. Different alkaloids and flavonoids were annotated with levels 2 and 3 of confidence using GNPS and MoNA spectral matches, and the ‘in-house’ *Ocotea*DB. ESI^+^ demonstrates representative reticuline alkaloid MS^E^ spectra and fragmentation product ions. ESI^−^—Clustering of predominantly *O*-glycosylated flavonoids identified across *Ocotea* spp. and respective aglycones. Each node represents an MS^E^-acquired mass spectrum, and the edges connecting them show MS/MS fragmentation similarity (cosine > 0.6). The pie charts show the relative abundance of each *Ocotea* plant species (*n* = 60). In MS^1^ scans, node diameters are related to the sum of peak regions of the precursor ion in both positive (upper) and negative (lower) modes of ionization.

**Figure 9:**
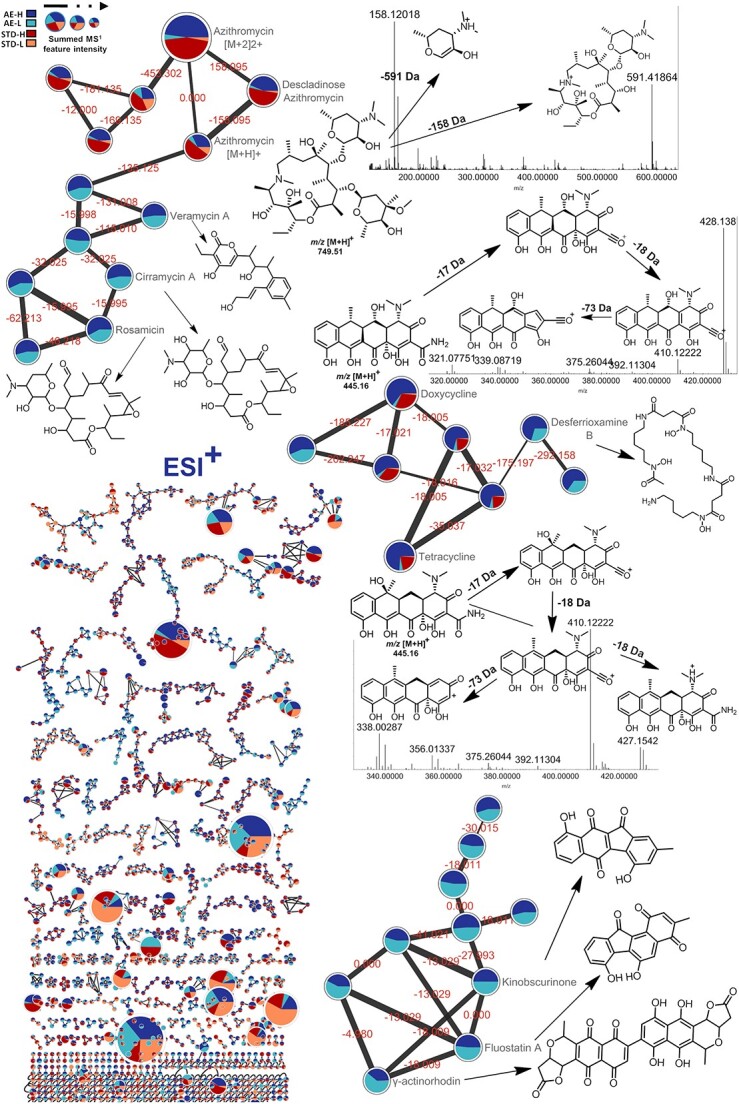
Molecular families of the actinobacterial extract derived from the FBMN at high and low concentrations of spiked chemical authentic standards. Different chemical standards were annotated with level 2 confidence using GNPS spectral matches, including azithromycin, tetracycline and doxycycline. Each node represents an MS^E^-acquired mass spectrum, and the edges connecting them show MS/MS fragmentation similarity (cosine > 0.6). The pie charts show the relative abundance of each sample (AE-H—actinobacterial extract spiked with a high concentration of standards, AE-L—actinobacterial extract spiked with a low concentration of standards, STD-H—chemical standards at high concentration and STD-H—chemical standards at low concentration). In MS^1^ scans, node diameters are related to the sum of the peak regions of the precursor ion in positive ionization mode.

Regarding the FBMN jobs with GNPS, positive mode analysis required ~5 h, whereas the negative mode took ~6 h. These analyses resulted in the generation of highly complex metabolic networks (Figures S6 and S7). Besides overall complexity, MN revealed intricate cluster families in the metabolome of *Ocotea* spp., which could be individually analyzed to get deeper information. In addition, to extract more nuanced insights, LC-HRMS-DIA/MS^E^ data were reprocessed with higher amplitude cutoffs (e.g. 50 000 counts). The FBMN jobs from both datasets required less than 15 min to finish. The simplified MNs generated from the reprocessed data aided in the visualization and identification of key *Ocotea* spp. molecular families (Figure 8) and actinobacterial MNs (Figure 9).

Spectra were queried against GNPS libraries related to our dataset (e.g. IQAMDB and NIH NPs for positive and negative mode, respectively) and a complete list of matches is listed in Tables 1 and S1 and online at the Zenodo open digital library (https://zenodo.org/records/10383866). The FBMN analysis revealed distinct families of aporphine and benzylisoquinoline alkaloids (positive mode), alongside predominately *O*-glycosylated flavonoids (negative mode) across the *Ocotea* spp. (Figure 8). The pie charts illustrate relative metabolite abundance across 60 *Ocotea* species based on MS^1^ precursor ion areas. Visual inspection of a positive mode alkaloid cluster shows 3-hydroxynornuciferine as highly abundant but specific to only a few *Ocotea* species, while glaucine to only a few others. Reticuline appears conserved across most *Ocotea* spp., suggesting a potential genus chemomarker. *N*-methylcoclaurine also arises broadly present, but with reduced abundance in this cluster family.

All of the spectral matches represented were thoroughly inspected to check their annotation and spectral similarity accuracy. Regarding the MN in negative mode, the highlighted family of glycosylated flavonoids is demonstrated, with the main metabolites across the highlighted cluster family including quercetin-3-*O*-rhamnoside (quercitrin), quercetin-3-*O*-galactoside (quercimetrin), quercetin-3-*D*-xyloside (reynoutrin), kaempferol-3-*O*-rhamnoside (afzelin), kaempferol-3-*O*-glucoside (astragalin) and kaempferol-3-*O*-arabinoside. The array of quercetin and kaempferol glycosides shows widespread distribution across the *Ocotea* spp. dataset. The broad interspecies conservation of these flavonoid metabolites suggests the presence of essential, shared biosynthetic pathways within the *Ocotea* genus.

## DISCUSSION

A core challenge in omics fields, including metabolomics, is the conversion and processing of DIA data (e.g. MS^E^) compared to traditional DDA workflows. In DDA, preselected precursors are fragmented, enabling straightforward data conversion and processing by most open tools. However, DDA induces significant losses in spectral data coverage, varying with the sensitivity of the instrument and, in relation, the cycle time of the method, because it selects only ions above a certain cut-off area or intensity for fragmentation [5, 28]. In contrast, MS^E^ methods fragment all ions without any previous precursor ion selection, generating complex but unbiased spectra with a more complete metabolomic data coverage [7]. The lack of predefined precursors in MS^E^ means that fragment–precursor relationships must be reconstructed post-acquisition through computational deconvolution approaches, which can be performed using different algorithms [5–7, 29]. This is more challenging compared to the inherent precursor-to-fragment associations made with DDA methods. Herein, we present an integrative strategy to leverage MS^E^ data in the community standard .mzML format using only freely available software and platforms. This study demonstrates the power of MS^E^ with accessible tools while highlighting current, ongoing challenges in data conversion, processing and interpretation.

This integrated pipeline provides an efficient and customizable solution for extracting the most biological information from MS^E^ data. To date, this is the first mention of the Waters2mzML in an applicability case, which is a recently introduced tool specifically designed to address the challenges associated with converting and centroiding Waters MS^E^ data, without the need to use vendor software such as UNIFI, Symphony or Progenesis QI. Waters2mzML is a simple tool that ensures compatibility and offers independence to convert raw Waters MS^E^ data into a more widely used format. This open tool can correctly reassign MS^2^-level data to MS^E^ MS/MS scans. Therefore, it is now possible to freely generate standard and functional .mzML spectra from MS^E^ data for further integrated downstream metabolomics analyses.

Moreover, this pipeline includes the ability to generate a custom, ‘in-house’ database through a KNIME workflow, enhancing its utility. This database integration into LC-HRMS- DIA/MS^E^ data processing significantly improves metabolite annotation of MNs when using MZmine and MS-DIAL. In our case studies, our focus was to enhance the annotation of metabolites specifically from *Ocotea* plants and marine actinobacteria datasets. The creation and incorporation of *Ocotea*DB and *Actinomarine*DB provided a tailored metabolite reference library to complement the capabilities of the open-source software tools. This pipeline has demonstrated efficacy and accuracy, enabling streamlined annotations at confidence levels 2 and 3 according to Metabolomics Standards Initiative (MSI) guidelines [30, 31].

Regarding data processing, the export functionality of MS-DIAL allows DIA data to be cosine matched with high-quality spectral libraries on the GNPS platform, also allowing integration with any GNPS tools for enhanced data interpretation, including FBMN, which was used in this workflow. It also allows other GNPS analyses, such as MS2LDA, Network Annotation Propagation (NAP) and MolNetEnhancer, which are already well-implemented for DDA data in the GNPS platform [32–34]. MS-DIAL software therefore enables the full processing of MS^E^ spectra with correct GNPS export in a generic file format (.mgf). Importantly, processed data from MS-DIAL can be employed for spectral similarity searches through a range of different algorithms and tools. The strength of MS-DIAL lies in its robust algorithm, MS^2^dec, which successfully deconvolutes precursor ions and reassociates precursor–fragment links and whose effectiveness has been widely proven [14, 19, 32, 33].

In contrast, MZmine 3 is a powerful software for processing and analyzing DDA data, while effectively handling DIA data and integrating with GNPS are still ongoing challenges. The dissociation of MS^1^–MS^2^ scans in DIA data remains a significant impediment for current MZmine 3. Full DIA-enabled algorithms within MZmine 3 are still in active development; nonetheless, the latest MZmine 3 can visualize DIA scans chronologically, enabling manual inspection of raw chromatograms and spectra, which was previously only possible with vendor-provided software. While the integration of DIA algorithms within MZmine 3 remains a work in progress, it has emerged as a well-known ecosystem for open MS data processing [11, 18].

Even though MZmine 3 could not export processed DIA data to GNPS, it was utilized as the software platform in this pipeline to visualize the metabolic fingerprints of the pooled QC and crude extract samples, as well as to perform accurate level 3 annotation (Figure 3), providing an integrative MS^E^ data analysis. The flexibility in setting up parameters is particularly beneficial for the sample alignment step, allowing researchers to make modifications and visualize new results without the need to reprocess previous steps, as required in MS-DIAL. The utilization of MZmine 3 therefore enabled us to generate precise metabolic fingerprint images of the crude extracts from both dataset cases. Manual annotation of metabolites matching the ‘in-house’ DBs and online spectral libraries, such as the MoNA spectra database, was also incorporated for increased confidence in the annotation of matched metabolites.

The great advantage of this integrated pipeline is the ability to reliably process the entirety of MS^E^ data, providing optimal visualization of MS^1^ and MS^2^ raw and processed data within a user-friendly and open software pipeline environment. Even though both programs used here present limitations, we tried to benefit from their advantages to help overcome the bottlenecks of MS^E^ data analysis with MN implementation. The extensive development of MZmine 3 is evident through its active GitHub community and frequent updates, showcasing its commitment to continuous improvement. This dynamic environment fosters innovation, and DIA implementation tools seem to be on the horizon. In contrast, MS-DIAL has seen slower recent development, less frequent updates and fewer data processing features and parameters, indicating the need for further improvements. However, the limitations of MS-DIAL do not diminish its effectiveness in performing the necessary tasks for DIA data analysis.

This workflow offers guidance to the community for handling LC-HRMS-DIA/MS^E^ (and AIF), for which standardized protocols were previously lacking. Future software developments (e.g. DIA algorithms in MZmine 3) will build upon, rather than invalidate, the core foundations established here, as we have delineated key data handling steps for DIA workflow implementation, from data conversion to parameter tuning (see Supplementary Section). Overall, this provides an open-source framework to empower DIA-AIF/MS^E^ users with customizable workflows for enhanced metabolomics analyses.

Specific parameter adjustments were performed to ensure reliable results for our MS^E^ data during FBMN jobs, considering that most MN examples available are based on DDA data. Given the larger size of the dataset and the complexity of MS^E^, we carefully modified search parameters such as cosine score, number of matched fragment ions and network organization parameters including TopK and maximum connected component size. It is crucial to fine-tune these parameters due to the lack of MS^2^ specificity in MS^E^ data, where fragment ions originate from all co-eluted precursors (see Methods section). Thus, the TopK value directly influences the number of edges retained in the network and should be considered, as it influences the connections between nodes and the overall structure of the MN. We highlight the importance of considering appropriate values for TopK in the investigation of molecular families of any DIA or MS^E^ data. In addition, DDA is generally less effective compared to DIA for low-abundance compounds [35]. Lowering cosine parameters is also applicable to DIA data since the search criteria need to be less restrictive for matches to occur. The association of these strategies provides a solid foundation for future improvements in metabolite identification and cluster analysis of DIA, AIF or MS^E^ data.

Furthermore, we recommend using more specific metabolite libraries in GNPS—like IQAMDB (IsoQuinoline and Annonaceous Metabolites Database) and NIH natural products—for broad metabolite coverage. DB-based annotation was consolidated with FBMN through feature metrics. For optimal annotation accuracy, curated, phylogeny-relevant libraries are preferable over comprehensive public counterparts. Targeted matching of detected metabolites to expected biosynthetic origins avoids erroneous assignments. Overall, harnessing biosynthetic knowledge through tailored libraries boosts reliability by connecting metabolites specifically to validated biological sources [36].

In this study, we rigorously demonstrate the utility and robustness of the DIA-IntOpenStream pipeline through its application to two distinct and carefully selected datasets, each chosen to showcase different aspects and capabilities of the workflow. In addition, the gas-phase fragmentation reactions were proposed for the different NP classes. The selection of dataset 1 was driven by its potential impact and applicability. Although, *Ocotea* spp. hold significant ethnobotanical importance, display promising medicinal potential and face taxonomic and ecological challenges. In addition, only a limited number of species within the genus have been chemically characterized. Given the high therapeutic potential of the *Ocotea* genus for drug discovery, there is an urgent need for NP chemical studies to support the bioprospecting use of *Ocotea* species, particularly those endangered in Brazil. Research topics focusing on the *Ocotea* genus have importance by themselves, and thus, this dataset also adds value and purpose to our study.

Dataset 2, an actinobacterial extract of MS public data spiked in high and low concentrations with 20 different chemical standards, was specifically chosen for a detailed comparative analysis with existing methods. The addition of known standards allows robust validation of the pipeline’s annotation accuracy and efficiency. By using an actinobacterial extract, we also demonstrate the workflow’s applicability to microbial metabolomics, an area of significant interest due to the role of microorganisms in environmental processes and human health. A comparative analysis with existing methods that have used this dataset highlights the advancements and improvements that DIA-IntOpenStream offers in terms of data processing efficiency, annotation accuracy and the ability to handle complex NP matrices. Several high-confidence annotations for both datasets were achieved. The results include a significant number of chemical annotations with level 2 confidence according to MSI guidelines [30, 31, 37, 38], with spectra having matched comprehensive spectral libraries of standard compounds (GNPS and MoNA).

Dataset 2, previously examined in a high-quality study [17]^,^ involved an advanced LC-HRMS analysis of complex NP mixtures. Among the strategies explored was the use of MS data acquired by DIA, specifically MS^E^. In that study, vendor software was used for data analysis, which is an extremely commonly used approach at the time of this study. We re-analyzed their DIA data with the IntOpenStream pipeline, and the obtained results reinforced the pipeline’s effectiveness. It successfully allowed the annotation of many authentic chemical standards in the complex NP microbial sources, a key indicator of its reliability. The initial study successfully identified 18 high-abundance and 16 low-abundance standards. In comparison, our pipeline yielded similar results, with a minimal difference of only two and three standards fewer at each concentration level, respectively (Figure S15 and Table S3).

On the other hand, our pipeline demonstrated enhanced efficacy regarding 5 main aspects. (i) FBMN matched seven authentic standards with annotation confidence level 2 (Table S3), surpassing the two standard matches in the original study that used classical MN. (ii) We obtained an additional seven annotations with confidence level 2 for the original actinobacterial extract by utilizing the built-in *Actinomarine*DB and manual data inspection (Figures S16–S19 and Table S4). (iii) FBMN analysis revealed various GNPS-matched standards and related compounds in the actinobacterial extract, including the annotation of rosamicin in the azithromycin standard cluster family. (iv) We uniquely detected important genus-specific compounds such as fluostatin A, kinobscurinone and γ-actinorhodin in the actinobacterial extract (Figures 9 and S14). (v) Lastly, our pipeline annotated 450 features at confidence level 3 (details published online at https://zenodo.org/records/10383866). As such, our approach not only aligns with existing literature data but also provides complementary insights. The robust comparison against a dataset of established benchmarks highlights the reliability and validity of DIA-IntOpenStream. Our commitment is to offer a freely accessible, robust tool for the metabolomics community to provide independence from the advantages of proprietary software.

As a final comment, the metabolite annotation of constitutional isomers, as observed in the *Ocotea* dataset, can be facilitated using standard compounds for combined MS/MS experiments. However, the stereochemistry of compounds with a high degree of structural similarity demands additional characterization to confirm chemical identity, as in the case of the aporphines boldine and isoboldine, which exhibit the same parent ion and product ions (Figure 4 and Table 1). The ratios and proportions of formed ion fragments differ and might help to elucidate isomers and epimers, at standardized MS conditions, for reliable spectral matching. Implementation of our pipeline enabled us to state the chemical diversity of the studied *Ocotea* species as mainly alkaloid producers. Multiple aporphine alkaloids bearing various substituent patterns were annotated with level 2 of confidence. A range of different glycoside flavonoids were annotated as well. Lastly, a wide variety of lignoids were annotated with level 3 confidence (available online at https://zenodo.org/records/10383866). It is worth mentioning that the first report on the evaluation of the chemical composition of several of these endemic *Ocotea* spp. in Brazil was published in 2023 [39].

Several metabolites not previously reported in the *Ocotea* genus were annotated at level 2 confidence. For instance, dehydronuciferine (24) annotated here in some *Ocotea* extracts has only been documented in other plants like the Nymphaeaceae family, encountered in the sacred lotus *Nelumbo nucifera*. NP research on the *N. nucifera* allowed authors to isolate the dehydronuciferine together with other aporphines such as the nuciferine (21) and nornuciferine (29), which are common compounds found in the *Ocotea* genus and also reported by us in the present investigation. Also, the alkaloid leucoxylonine (37) is reported in the literature as produced only by two species of the *Ocotea* genus, including *Ocotea leucoxylon* and *Ocotea minarum* [40, 41]. In this work, it was successfully annotated in other species with high-intensity peak areas, for the first time, in the VZ, VA and PU *Ocotea* extracts (Table S2). In this manner, the present research is also filling this gap and might contribute with chemical characterization data to further taxonomic classification studies associating chemosystematics strategies.

Using a custom database of metabolites previously isolated from the same biological source greatly aids annotation confidence. Large databases can complement this approach but require careful analysis to avoid improbable assignments. Our ‘in-house’ DBs built in KNIME enabled high-confidence annotations since matched compounds were previously isolated in the targeted genera of the studies. For study cases of biological samples such as urine and blood, a range of other databases are available in HMDB (https://hmdb.ca/) as well as other online repositories. In addition, automated annotation with a higher level of confidence can be also performed in MS-DIAL with metabolomics MSP spectral kits or by directly exporting data to the GNPS platform and selecting available spectral libraries. Critically important to perform quality analyses, manual and automated annotations were largely complementary. For example, dataset 1 contained only 18 common level 2 annotations, indicating both strategies are relevant and that combining them can be highly effective.

In conclusion, all these ongoing challenges around LC-HRMS/DIA analysis have motivated us to build this pipeline. We believe it represents an advancement in the field, providing an accessible and efficient workflow for handling complex MS^E^ data and conducting MN analyses. It can globally aid bioprospecting NP, as we did by unlocking the chemical diversity of plants and bacterial marine extracts. Also, the inclusion of known standards in dataset 2 allowed robust validation of the pipeline’s annotation accuracy and efficiency. The use of both datasets highlighted DIA-IntOpenStream’s versatility and potential in diverse metabolomics studies. By prioritizing accessibility and transparency, our pipeline ensures that all aspects of data analysis, including processing steps, parameters, software versions and computational setup, are precisely documented and available to the scientific community. This commitment to reproducibility fosters scientific progress and collaboration. Future works may integrate other valuable open data pre-processing, MS/MS annotation and *in silico* fragmentation tools into this pipeline, such as the TidyMS python library, SIRIUS software and MS-FINDER, respectively [42–44]. Overall, this pipeline embodies scientific rigor, and its implementation holds promise for speeding up chemical discoveries, ultimately guiding researchers toward a more collaborative and open environment for research.

## METHODS

### Solvents, plant material and crude extract preparation

Details regarding the solvents used and sample preparation methods are provided in the Supplementary Material SM-1. Information on solvent sources, purity levels, vegetal material, maceration extraction conditions and sample-handling procedures are all included.

### Data acquisition and sample analysis

Chromatographic analysis was performed on an ultra-performance liquid chromatography–quadrupole time-of-flight tandem MS instrument (Xevo qTOF MS, Waters Corp., Milford, USA). Details concerning the QC preparation, chromatographic column, method details and mobile phase system information are described in Supplementary Material SM-2. The electrospray ionization (ESI) source operated in both positive and negative ion modes to capture a comprehensive range of analytes. MS^E^, a type of DIA analysis, was conducted using MassLynx™ (v4.2; Waters Corp., Milford, USA). The mass spectrometer and MS^E^ acquisition parameters are fully detailed in Supplementary Material SM-2.

### Public samples dataset

We validated our pipeline using a publicly available LC-HRMS-DIA/MS^E^ dataset of a marine actinobacteria extract. This dataset, as described in the publication by Carnevale *et al.*, was enriched with a pool of 20 authentic standards [17]. The chosen standards encompass a wide range of antimicrobial and chemotherapeutic agents, along with naturally occurring compounds, thereby providing a diverse chemical profile suitable for comprehensive analysis. The dataset was obtained from the MassIVE repository (MSV000088316) and is accessible through the Global Natural Products Social Molecular Networking (GNPS) platform.

### Data processing and annotation workflow

The KNIME workflow and subsequent open software in the pipeline were executed on a Windows 11 desktop computer with a 12-core (8 used) 64-bit Intel Core i7-12700—2.10 GHz processor with 32 GB of RAM. The GPU consisted of an NVIDIA T1000 8GB.

### KNIME in-house database workflow

To perform the experiments, we have developed a robust workflow to establish an integrated ‘in-house’ database within Mzmine and MS-DIAL data processing software using the KNIME (University of Konstanz, Zurich, Switzerland, version 4.6.5). KNIME (www.knime.org) is an open-source workflow system with a graphical user interface built on a set of nodes known as ‘extensions’ that process data and transmit it via connections between those nodes. Thus KNIME provides a simple visual workbench that allows scientists to build and visualize complex workflows [45, 46]. The workflow is online and is publicly available to use (https://hub.knime.com/-/spaces/-/~8bZEbbknV8tVptea/current-state/). Details regarding our custom ‘in-house’ DB are provided in the SM-3. The ‘in-house’ database allows level 3 annotation following the guidelines of the MSI [30, 31]. However, it holds more confidence because the ‘in-house’ DB supports fast annotation of previous metabolites previously isolated in the family or genus of the study.

### MS data conversion

To ensure compatibility, accessibility and comparability, the raw Waters MS^E^ data from both independent datasets were converted to the widely used .mzML format using the recently developed open-source tool Waters2mzML 1.2.0, available on GitHub (https://github.com/AnP311/Waters2mzML) (SM-3). The generated .mzML files can be readily processed using Mzmine 3 and MS-DIAL software for further analyses and interpretation.

### Mzmine 3 data processing and analysis

The raw data containing peak area and *R*_t_–*m/z* pairs of 71 *Ocotea* samples (60 *Ocotea* spp. sample extracts, four QCs, four blanks and three VI sample extracts [replicates]) and 17 samples from actinobacterial extract (replicates and blanks), previously converted to .mzML format, were imported into MZmine 3.4.27 (https://mzmine.github.io/; MZmine Development Team). One QC and one blank sample replicate were excluded from processing due to higher shifts in the *R*_t_ compared to other replicates. The detected peaks were deconvoluted, isotopes were eliminated, identical peaks in the different chromatograms were aligned, the remaining gaps were filled, duplicated features were filtered and the blank chromatograms were subtracted. Then, the features were annotated according to their monoisotopic masses. Data from each ionization mode were processed separately. The data processing parameters are fully detailed in the Supplementary material SM-4.

The treated MS data was then exported in .xlxs format. The Mass Bank of North America (MoNA) (https://mona.fiehnlab.ucdavis.edu/) was used for manual spectral comparisons and fragment MS data matches. These manual annotations were listed as level 2 according to the current standards initiative [31, 37, 38, 47]. The chosen modules and algorithms of processing were the standard ones, although this software offers an array of different modern tools that can be used to improve data processing results [6, 11, 18].

### MS-DIAL data processing and analysis

The ‘Analysis Base File’ (.abf) format, generated using Reifys Abf converter software (https://www.reifycs.com/AbfConverter), is a traditional data format for MS-DIAL MS^E^ data processing aimed at MN implementation. However, to ensure maximum compatibility with other software, we chose a more universal approach by converting the data into .mzML format, enabling simultaneous MZmine and MS-DIAL usage and MS data comparison.

The converted .mzML data were successfully loaded into MS-DIAL version 4.9.2 (http://prime.psc.riken.jp/compms/msdial/main.html) for data processing, following a procedure similar to the one used with MZmine 3. In MS-DIAL, we configured the project settings according to our specific data requirements: ionization mode (soft ionization; chromatography; conventional DIA-all-ions method-AIF), the experiment file (available in the Supplementary Material) and data type (i.e. centroid MS^1^ and MS/MS data). It is also necessary to process positive and negative ion modes data separately. For data processing, the parameters are fully detailed in the Supplementary Material (SM-5). Subsequently, the data were uploaded to the GNPS server using the open FTP tool named WinSCP (https://winscp.net/eng/download.php). All the other existing parameters not mentioned were left at software default.

### Molecular networking and metabolite annotation analysis

Metabolite annotation in our study involved a combination of automated and manual approaches (detailed in SM-6). Post-data processing is performed by exporting the results from MS-DIAL, i.e. ‘MS2 File’ (.mgf), ‘Feature Quantification Table’ (.txt) and the metadata to the GNPS (https://gnps.ucsd.edu/) environment. The metadata file was built in .txt format with the filenames and the respective attributes of species, sample type, region/state of plant collection and endemic occurrence in Brazil. These files are available online on the Zenodo platform (https://zenodo.org/records/10383866). FBMN was generated using the respective workflow in the GNPS ecosystem [48] using FBMN parameters described in SM-6. Most of the metabolites were annotated at levels 2 and 3 according to MSI levels. All combined FBMN jobs with level 3 annotated metabolites are listed in Tables S3 and S4 and can be found on the Zenodo platform (https://zenodo.org/records/10383866).

### Molecular networking visualization and interpretation

The generated networks from GNPS were downloaded and visualized using Cytoscape network software (version 3.8.2). The metadata-rich GNPS table, when opened in Cytoscape, can be exported as a .csv file. This facilitates semi-automated integration with the ‘in-house’ annotation. Subsequently, the annotated table can be reimported into the software to perform MN investigation and analysis.

**Key Points**

- An open, integrated workflow is presented that leverages both universal data formats (.mzML) and open-source software tools (KNIME, MZmine, MS-DIAL and GNPS) for enhanced DIA-MS^E^ data handling.
- The workflow demonstrated its applicability by characterizing *Ocotea* crude plant and marine actinobacterial extract, revealing the chemical diversity of different natural product classes.
- By promoting open science, the pipeline provides a framework to advance DIA-MS^E^ data handling, transparency, reproducibility and analysis through integrative approaches, overcoming the limitations of commercial solutions.
- We aim to propel the field forward, empowering researchers to achieve more accessible MS^E^ data processing, with a reliable annotation process, leveraging the potential of DIA-MS to drive the community toward further improvements.

## Supplementary Material

Revised_supplemental_material_bbae013

## Data Availability

The *Ocotea* dataset of the current study was deposited to the Mass Spectrometry Interactive Virtual Environment (MassIVE) repository. The data that support the findings of this study are available at ftp://MSV000093006@massive.ucsd.edu under the registry code MSV000093006. Guidelines for files submitted to MassIVE for public access can be found online (https://massive.ucsd.edu/ProteoSAFe/static/massive.jsp).

## ADDITIONAL INFORMATION

Accompanying information supplements this paper online at Zenodo open digital library: https://zenodo.org/records/10383866.

## Author Biographies

**Albert Katchborian-Neto**, a doctoral candidate at the Institute of Chemistry of the Federal University of Alfenas, Brazil, focuses his research on natural products by applying mass spectrometry and machine learning to metabolomics for exploring pharmacological applications with particular interest in the psychedelics science.

**Matheus F. Alves**, a master’s student at the Institute of Chemistry of the Federal University of Alfenas, Brazil, focuses his research on mass spectrometry-based metabolomics, exploring psychedelic natural products and biodiversity bioprospecting studies.

**Paula C.P. Bueno** is a scientist at the Leibniz Institute of Vegetable and Ornamental Crops (IGZ), Germany, and a guest researcher at the Chemistry Institute of the Federal University of Alfenas, Brazil. Her research line is focused on plant metabolomics, ecophysiology and chemical ecology.

**Karen de Jesus Nicácio**, a professor at the Chemistry Department at the Federal University of Mato Grosso, Brazil, researches natural products by isolating and identifying metabolites from plants and microorganisms using classical and modern metabolomics approaches.

**Miller S. Ferreira** is a post-doctoral researcher at the Institute of Chemistry of the Federal University of Alfenas, Brazil. His research interests include chemometrics and machine learning techniques applied to the chemistry of natural products.

**Tiago B. Oliveira** is a professor at the Department of Pharmacy of the Federal University of Sergipe, Brazil. His research interests are green chemistry methods including metabolomics, biochemometrics and virtual screenings to obtain food safety.

**Henrique Barbosa** is a doctoral student at the Center of Natural Sciences and Humanities at the Federal University of ABC, Brazil, focusing his research on bioprospecting natural resources using computational methods.

**Michael Murgu** is a senior scientist at Waters Corporation, São Paulo. He has expertise in metabolite characterization, tandem mass spectrometry and high-performance liquid chromatography, to drive innovation in mass spectrometry technologies.

**Ana C.C. de Paula Ladvocat** is a professor at the Department of Pharmacy at the Federal University of Juiz de Fora. Her interests are mainly in stem cells for regenerative medicine, modeling diseases and assessing pharmacological properties.

**Danielle F. Dias**, a professor at the Institute of Chemistry of the Federal University of Alfenas, Brazil, works with the synthesis and characterization of novel bioactive compounds with analgesic, anti-inflammatory, antimicrobial and antiparasitic activities.

**Marisi G. Soares**, a professor at the Institute of Chemistry of the Federal University of Alfenas, Brazil, researches natural products using chromatography, nuclear magnetic resonance and mass spectrometry for structural characterization and identification.

**João H.G. Lago** is a professor in the Center of Natural Sciences and Humanities at the Federal University of ABC, Brazil. His research interests involve the discovery of bioactive natural products, especially those with antiparasitic, anti-inflammatory or anticancer activity.

**Daniela A. Chagas-Paula**, a professor at the Institute of Chemistry of the Federal University of Alfenas, Brazil, focuses her research on natural products chemistry and implementation of metabolomics, biochemometric modeling and pharmacological evaluations, with additional interests in the biochemical basis of Parkinson’s disease.
