# Supplementary material for: Integrative open workflow for confident annotation and molecular networking of metabolomics MSE/DIA data
Source: Brief Bioinform. 2024 Feb 7;25(2):bbae013. doi: 10.1093/bib/bbae013 (PMC10849173; doi:10.1093/bib/bbae013)

**Integrative open workflow for confident annotation and molecular networking of metabolomics MS^E^/DIA data**

Albert Katchborian-Neto^1^, Matheus Fernandes Alves^1^, Paula Carolina Pires Bueno^1,2^, Karen de Jesus Nicácio^3^, Miller Santos Ferreira^1^, Tiago Branquinho Oliveira^4^, Henrique Barbosa^5^, Michael Murgu^6^, Ana Cláudia Chagas de Paula Ladvocat^7^, Danielle Ferreira Dias^1^, Marisi Gomes Soares^1^, João Henrique Ghilardi Lago^5^, Daniela Aparecida Chagas-Paula^1^*

^1^Chemistry Institute, Federal University of Alfenas, 37130-001, Alfenas, Minas Gerais, Brazil

^2^Leibniz Institute of Vegetable and Ornamental Crops (IGZ), Theodor-Echtermeyer-Weg 1, 14979, Großbeeren, Germany

^3^Department of Chemistry, Federal University of Mato Grosso, 14040-901, Cuiabá, Mato Grosso, Brazil

^4^Department of Pharmacy, Federal University of Sergipe, 49100-000, São Cristóvão, Sergipe, Brazil

^5^Center of Natural Sciences and Humanities, Federal University of ABC, 09210-180, Santo Andre, São Paulo, Brazil.

^6^Waters Corporation, Alameda Tocantins 125, 27th floor, Alphaville, 06455-020, São Paulo, São Paulo, Brazil

^7^Department of Pharmaceutical Sciences, Federal University of Juiz de Fora, 36036-900, Juiz de Fora, Minas Gerais, Brazil

**Supplementary material**

**Supplementary Figures**…………………………………………………………………….....1

**Supplementary Tables**……………………………………………….…….………………..20

**Supplementary Texts**…………………….…………..……………….….………………….39

**Supplementary Protocol**..................................................................................................…...46

**Supplementary Material and Methods**…..............................................................................54

**Supplementary FMBN jobs and Zenodo links**...…………………...………………………60

**Supplementary Figures**

**
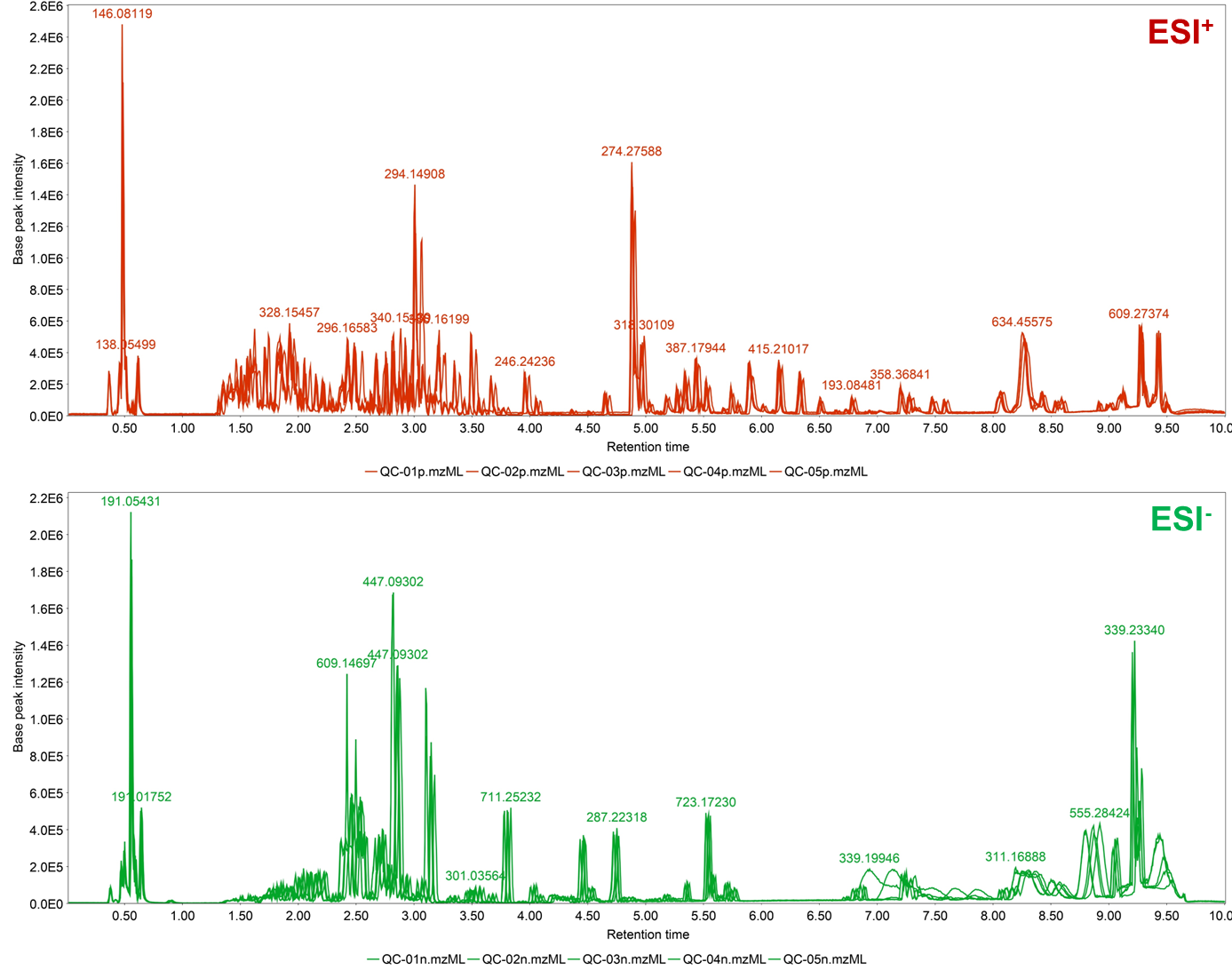
**

**Fig. S1 | LC-HRMS-MS^E^/DIA metabolic fingerprints shown as Base Peak Ion (BPI) chromatograms displaying the overlapped quality control (QC) replicates (pooled from 60 *Ocotea* spp. leaf extracts).** The QC replicates were acquired in both positive (red) and negative (green) electrospray ionization modes under similar conditions. The overlay of the BPI traces for 5 replicate injections demonstrates highly reproducible chromatographic and mass spectrometric performance essential for robust comparative metabolomics analysis.

**
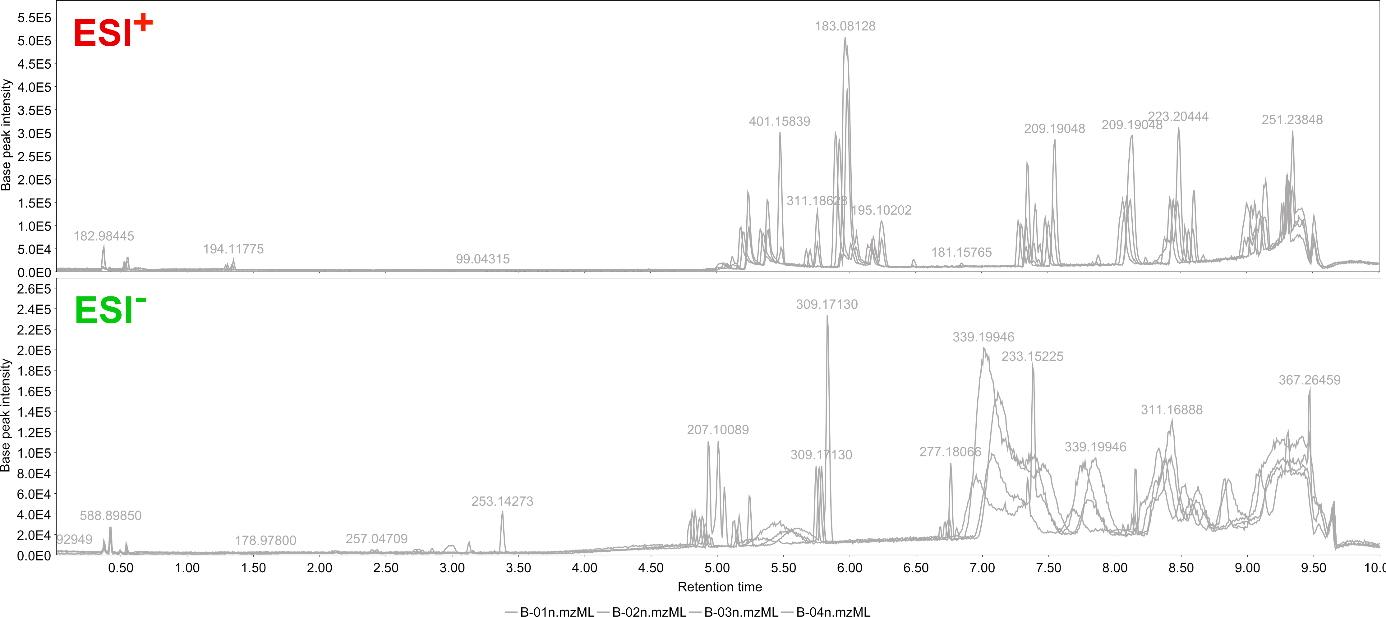
**

**Fig. S2 | LC-HRMS-MS^E^/DIA fingerprints shown as Base Peak Ion (BPI) chromatograms displaying the overlapped blank samples from the *Ocotea* dataset 1.** The blank replicates were acquired in both positive (red) and negative (green) electrospray ionization modes under identical conditions. The overlay of the BPI traces for 5 replicate injections demonstrates highly reproducible chromatographic analysis.

**
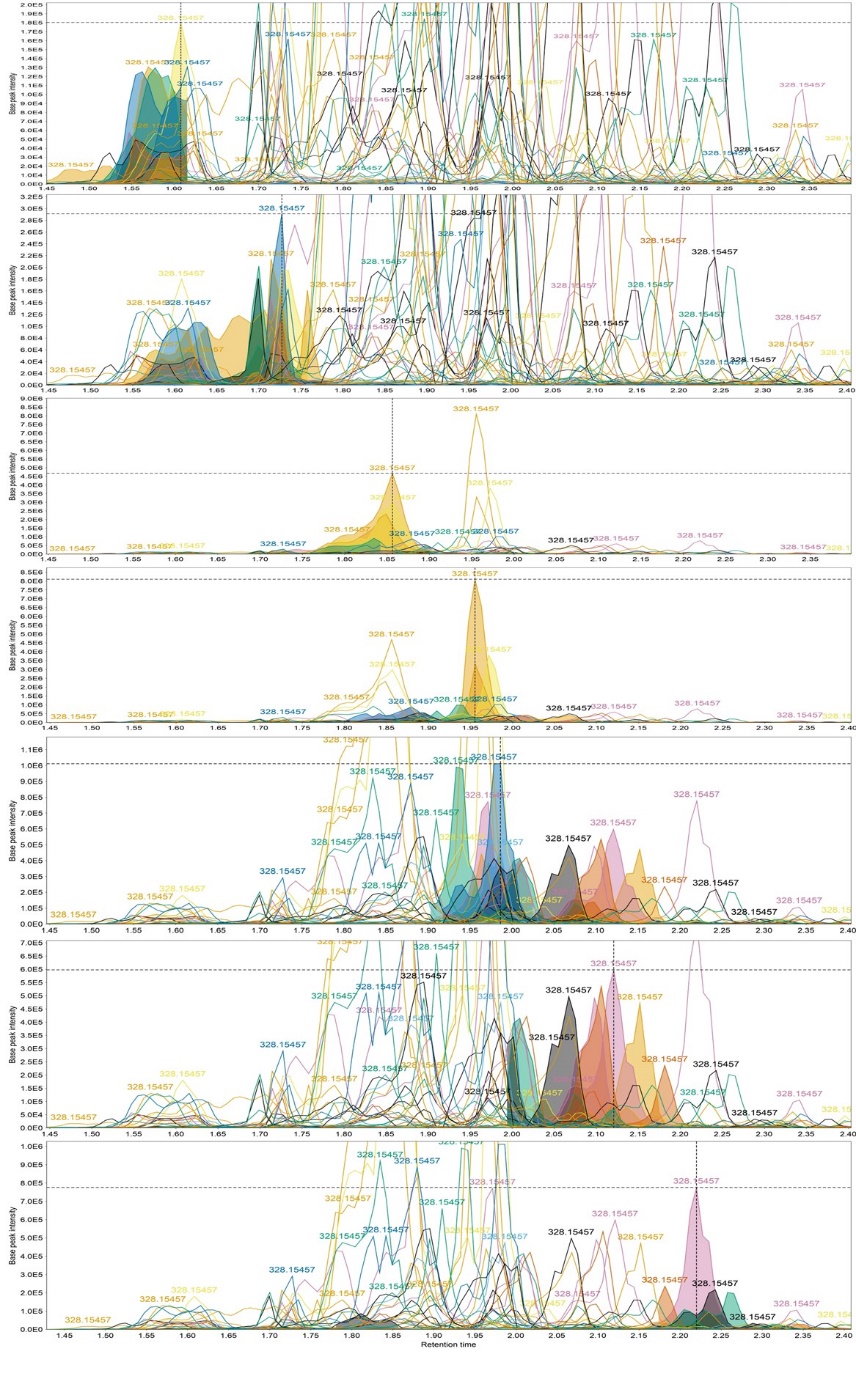
**

**Fig. S3 | Aligned chromatograms from *Ocotea* spp. samples, with zoom on the features with *m/z* 328.154 on a 1.45-2.40 min retention time (R_t_) window.** The overlaid chromatograms exhibit precise retention time alignment of the isomeric compounds and high resolution of the close features.

**
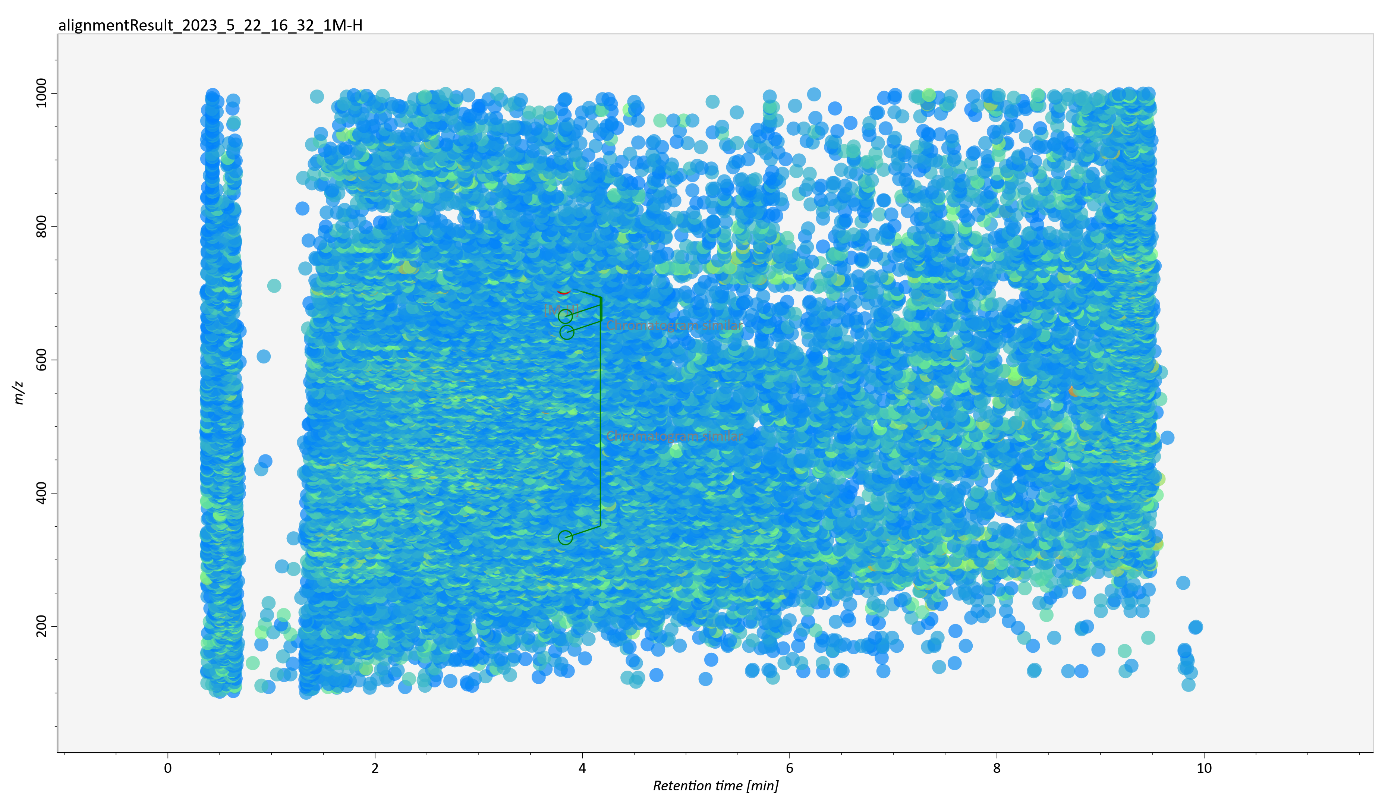
**

**Fig. S4 | MS-DIAL alignment result of negative ionization mode from *Ocotea* dataset.** Each dot (circle format) represents a particular feature of the processing with the colour pallet from blue to green varying with the intensity, where the green ones represent the most intense ions. In detail, the ions at *m/z* 711.2523 and R_t_ 3.82.

**
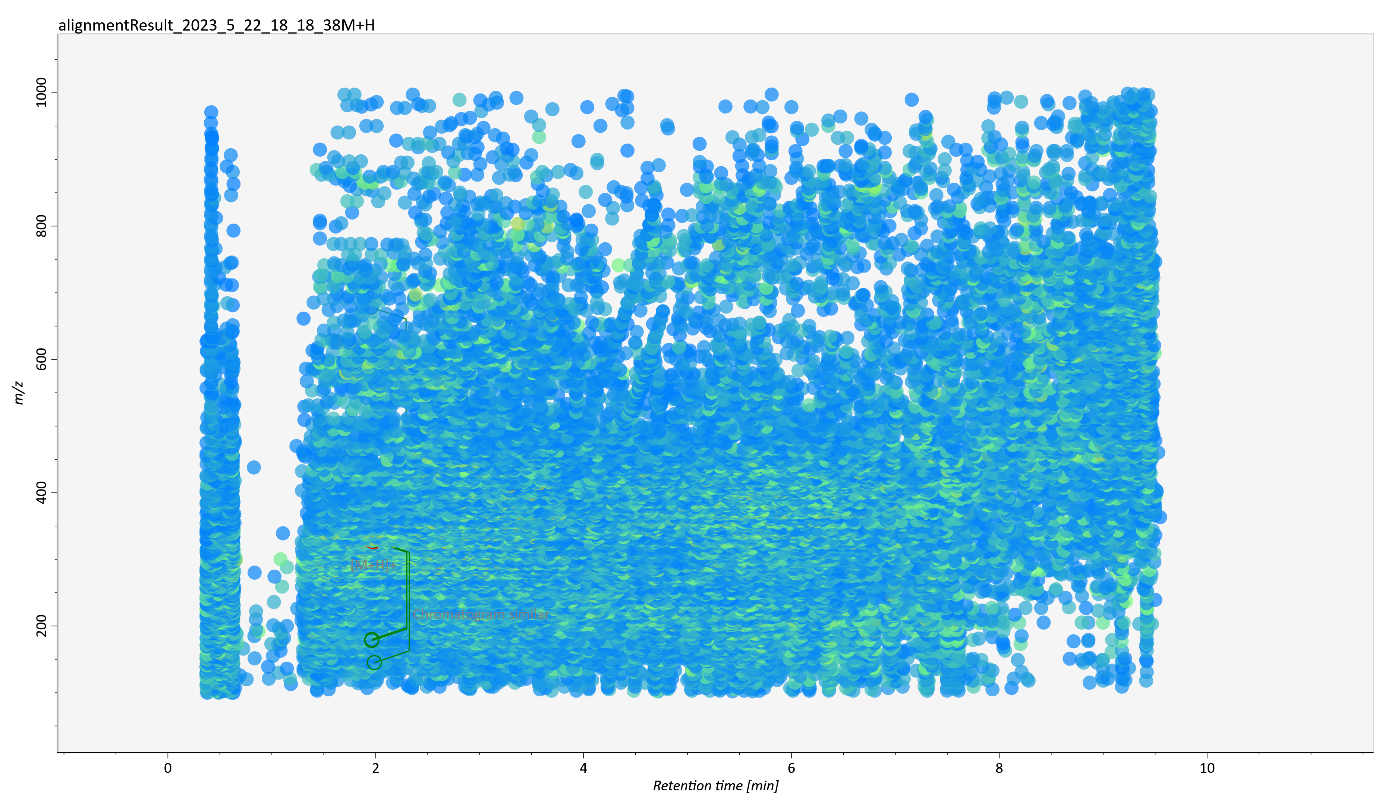
**

**Fig. S5 | MS-DIAL alignment result of positive ionization mode from *Ocotea* dataset.** Each dot (circle format) represents a particular feature of the processing with the same variation on the colour pallet. In detail, the ions at *m/z* 328.1546 and R_t_ 1.98.

**
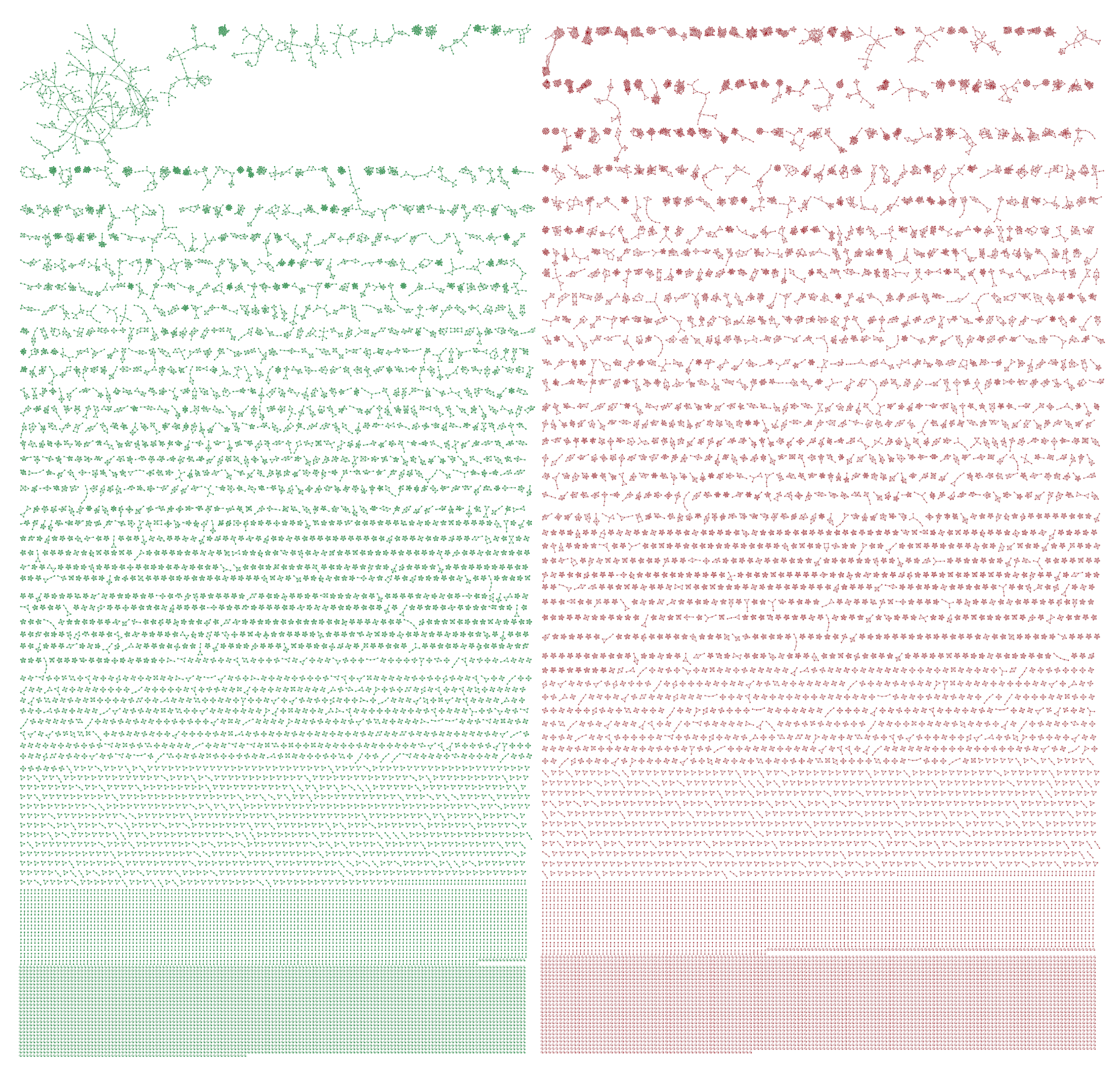
Fig. S6 | FBMN of positive (red) mode of ionization from *Ocotea* dataset.** Each node on networks represents a feature (*m/z*/ R_t_) from MS-DIAL processing connected with a basis on MS/MS spectral similarity. 22.572 nodes are shown on positive modes. The amplitude used in the MS-DIAL data processing for visualization of these MN was set at 1000. The complexity of our data is illustrated by these networks, showing that complex matrices as plant extracts aligned in big datasets (n=60) demand sophisticated tools for reliable processing, visualization and interpretation of the results.

**
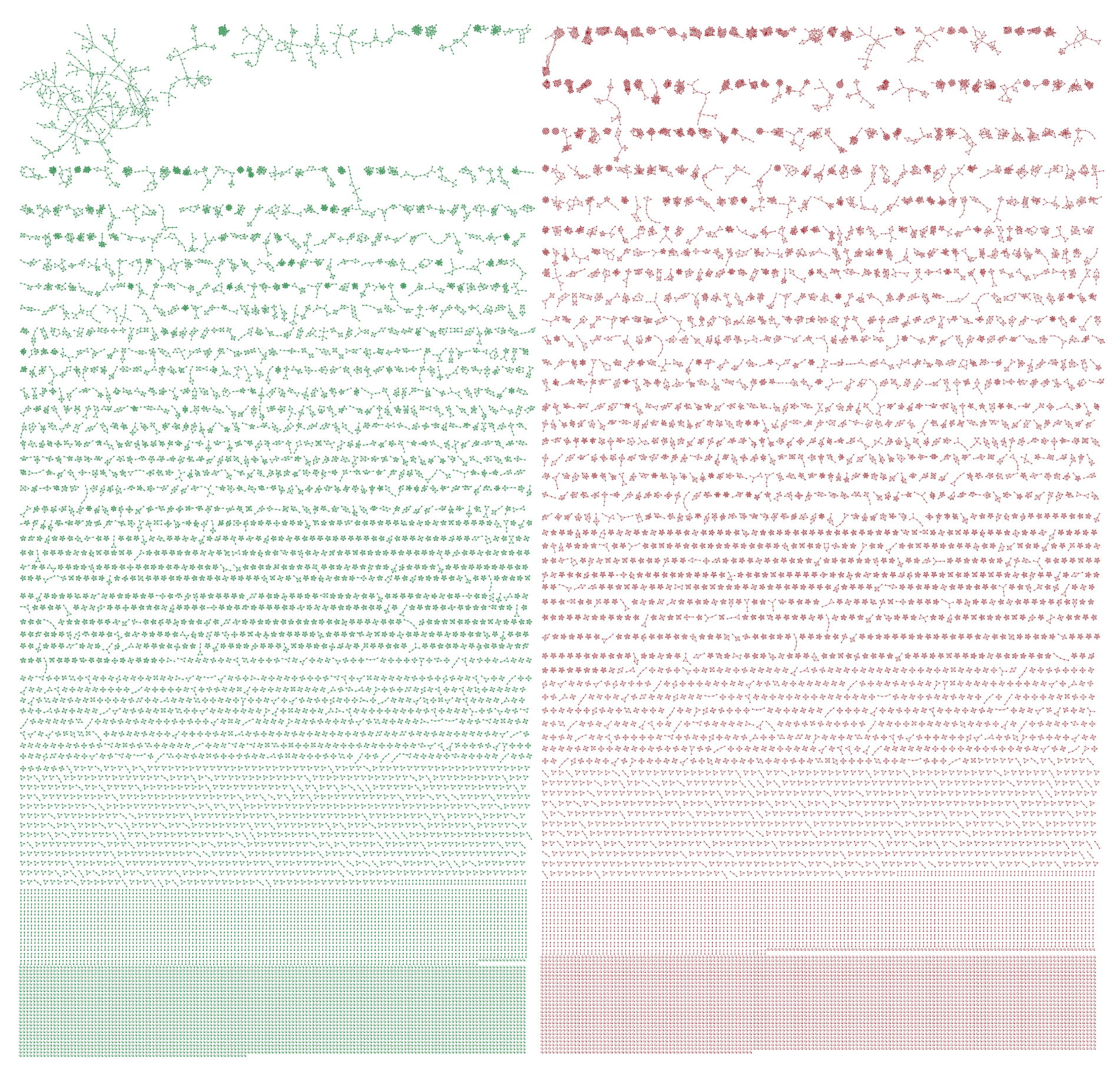
Fig. S7 | FBMN of negative (green) mode of ionization from *Ocotea* dataset.** Each node on networks represents a feature (*m/z*/ R_t_) from MS-DIAL processing connected with a basis on MS/MS spectral similarity. 21.838 nodes are shown in negative mode. The amplitude used in the MS-DIAL data processing for visualization of these MN was set at 1000. The complexity of our data is illustrated by these networks, showing that complex matrices as plant extracts aligned in big datasets (n=60) demand sophisticated tools for reliable processing, visualization and interpretation of the results.

**Fig. S8 | Chemical structures annotated for the *Ocotea* dataset with level 2 of confidence (ID 1-16).**

**Fig. S9 | Chemical structures annotated for the *Ocotea* dataset with level 2 of confidence (ID 17-32).**

**Fig. S10 | Chemical structures annotated for the *Ocotea* dataset with level 2 of confidence (ID 33-48).**

**Fig. S11 | Chemical structures annotated for the *Ocotea* dataset with level 2 of confidence (ID 49-60).**

**Fig. S12 | Chemical structures annotated for the *Ocotea* dataset with level 2 of confidence (ID 61-66).**


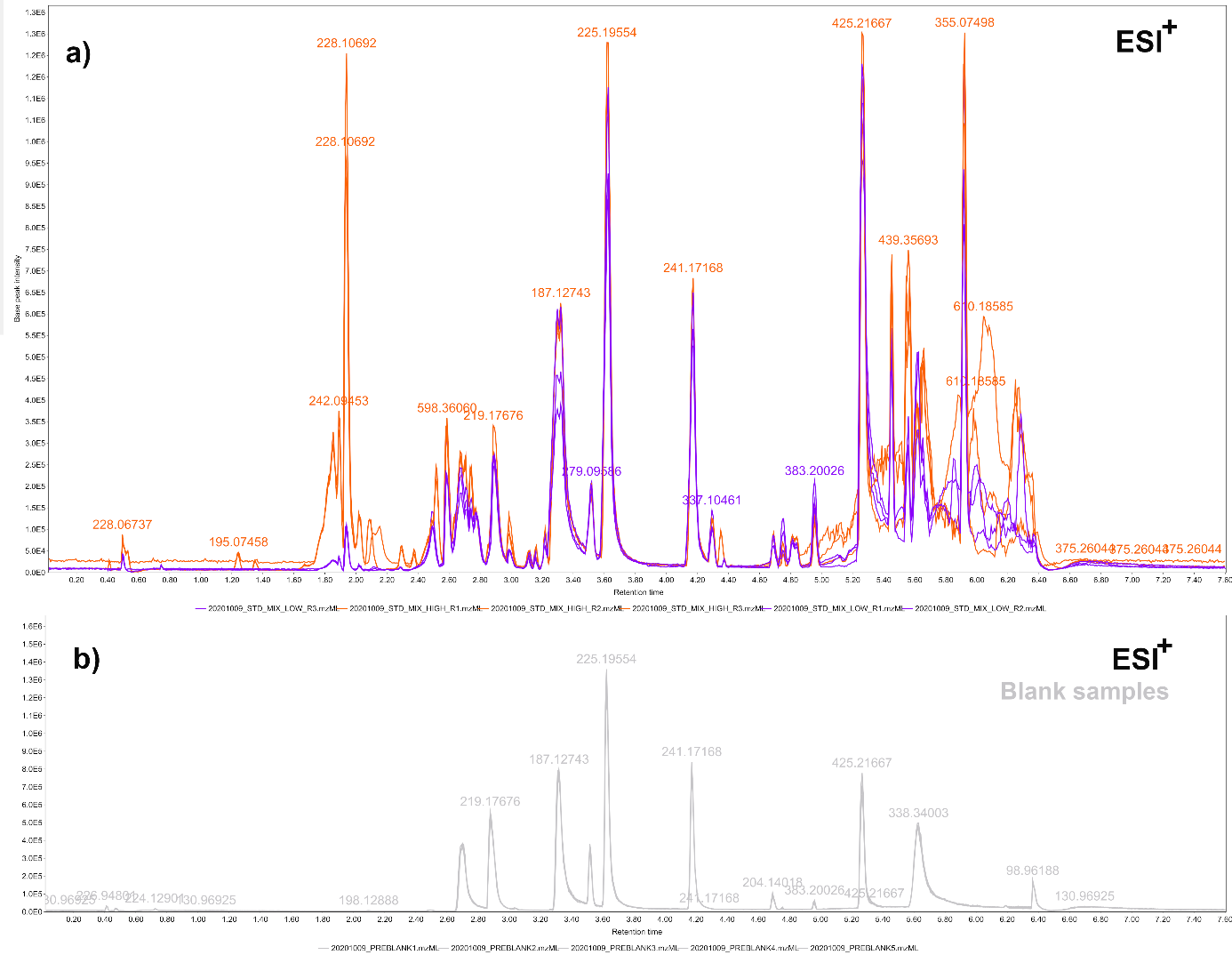


**Fig. S13 | LC-HRMS-MS^E^/DIA metabolic fingerprints shown as Base Peak Ion (BPI) chromatograms displaying in purple the overlapped actinobacterial extract spiked with chemical authentic standards in high (purple) and low (orange) concentration. (b) the overlapped blank replicates in the grey colour.** The actinobacterial extract and blank replicates were acquired only in positive electrospray ionization. The overlay of the BPI traces for 6 samples and 5 blank replicate injections demonstrates highly reproducible chromatograms.

**
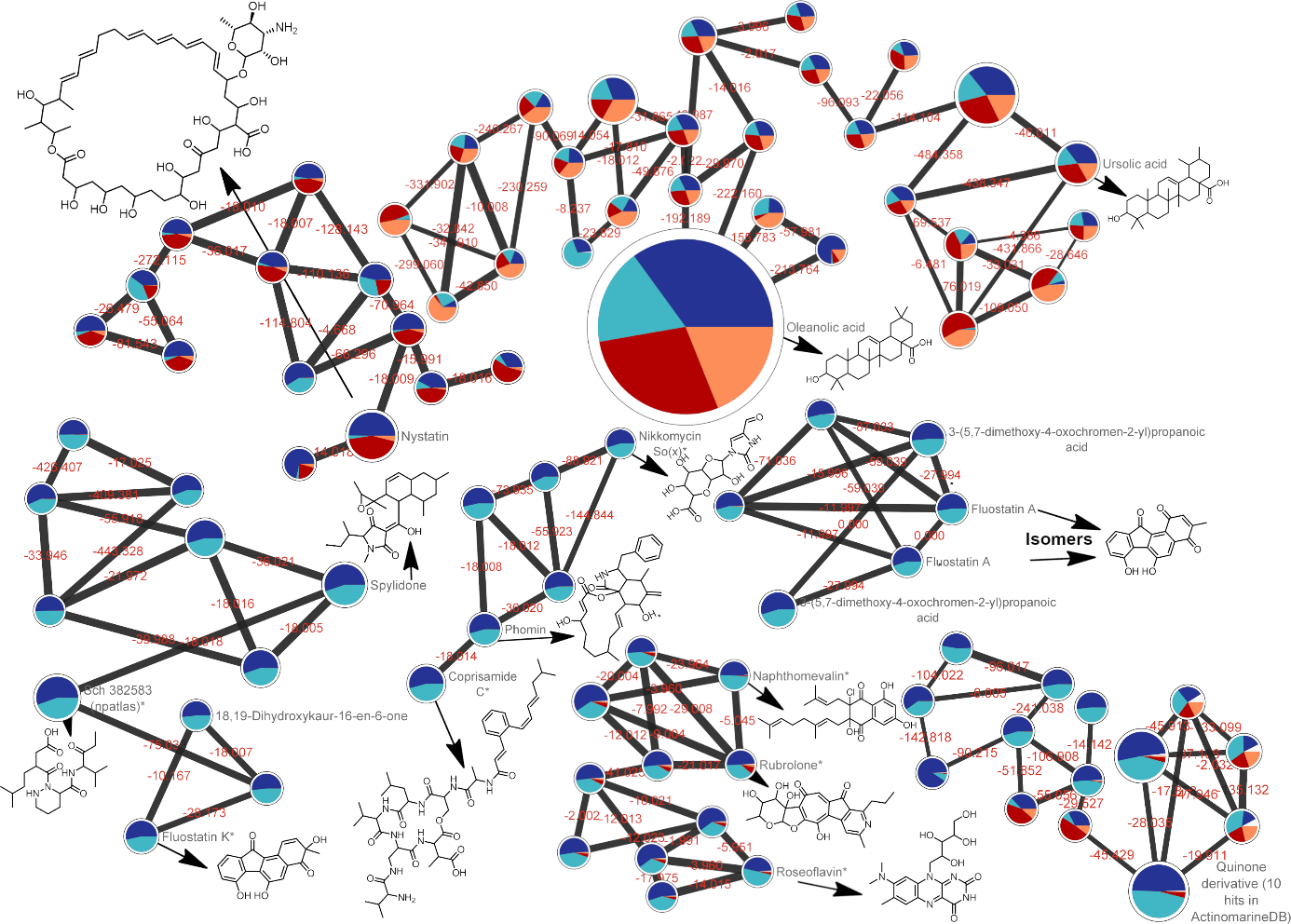
**

**Fig. S14 | Molecular families derived from the Feature-Based Molecular Networking (FBMN) for the Actinobacterial dataset.** Several marine NP were annotated with levels 2 and 3 of confidence using GNPS and MoNA spectral matches, and *in-house* *Actinomarine*DB. Besides, nystatin, oleanolic acid and ursolic acid were annotated as chemical standards spiked in the extracts. Pie chart colours refer to node distribution in extracts and standard samples. Blue colours represent actinobacterial extracts spiked with high (blue dark) and low (light blue) standard concentrations. Red and orange colours represent the pooled chemical standards samples, in high (red) and low (orange) concentrations, respectively. Fluostatin A isomers were clustered and automatically annotated as fluostatin A from GNPS libraries. Whereas, the coprisamide C, phomin and nikkomycin So(x) were clustered and annotated at level 3 of confidence by the monoisotopic mass match with *Actinomarine*DB. As well fluostatin K and spylidone, alongside the roseoflavin, rubrolone, and naphthomevalin MN clusters. The bottom right cluster family presented a node related to ten level three annotations from *Actinomarine*DB, which were annotated as quinone derivatives, e.g 8-*O-*methyltetragomycin, fujianmycin B, hatomarugibin A and B, zoumbericin A, brasiliquinone B and homo-dehydrorabelomycin E, rubiginone M, and two others NP not named from our *in-house*DB. *Level 3 of confidence annotations.








**Fig. S15 | All chemical structures of standards annotated from the dataset 2.**

**Fig. S16 | Chemical structures of metabolites annotated with level 2 of confidence based on *Actinomarine*DB.**

**Fig. S17 | Main proposed fragmentation pattern of some annotated chemical standards used for dataset 2.** 3a) Azithromycin. 9a) Tetracycline. 10a) Doxycycline.

**Fig. S18 | Main proposed fragmentation pattern of some level 2 annotated metabolites from the *Actinomarine*DB used for dataset 2.** 1b) Fluostatin A. 4b) Rosimicin.

**Table S1 | Details of metabolite annotation with level 2 of confidence for the *Ocotea* dataset.** Putative annotations of metabolites identified by matching accurate mass, isotopic pattern, and fragmentation spectra to public databases literature sources and chemical knowledge. Putative name, cLogP, molecular formula, observed *m/z, a*dducts, main fragments, database sources, and supporting international identifier (InChIKey) are provided.

| ID | Putative metabolite name | Observed *m/z* | Adduct | MS/MS fragments | Reference | InChIKey |
| --- | --- | --- | --- | --- | --- | --- |
| 1 | 4-hydroxy-*N*-methylproline | 146.08119 | [M+H]^+^ | 82.06643; 100.07568 | Proposed | FMIPNAUMSPFTHK-UHNVWZDZSA-N |
| 2 | Crotsparine | 284.12770 | [M+H]^+^ | 118.06763; 146.06143; 152.06110; 165.07108; 178.07968; 235.0754; 267.1027; | GNPS | LCAZZISCNMBVKG-LBPRGKRZSA-N |
| 3 | Glaziovine | 298.14359 | [M+H]^+^ | 141.07013; 152.06110; 165.06844; 167.08461; 178.07695; 194.07216; 235.0754; 267.1027; | GNPS | PNJUPRNTSWJWAX-UHFFFAOYSA-N |
| 4 | *N*-methylcoclaurine | 300.15932 | [M+H]^+^ | 103.05380; 107.04881 115.05604; 165.07108; 194.07501 | GNPS | BOKVLBSSPUTWLV-INIZCTEOSA-N |
| 5 | 3-hydroxynornuciferine | 298.14355 | [M+H]^+^ | 152.06110; 165.07108; 177.06862; 178.07695; 189.06778; 195.07916; 205.06406; 218.07373; (281.14987) | GNPS | AOGVVFDNSYRXJL-CYBMUJFWSA-N |
| 6 | Laurelliptine | 314.13850 | [M+H]^+^ | 151.07413; 237.07719; 238.08591; 255.08789; 265.07416; 266.08069; 283.08377; 297.09570; 298.10825 | Proposed | HORZNQYQXBFWNZ-UHFFFAOYSA-N |
| 7 | Laurolitsine | 314.13853 | [M+H]^+^ | 165.06844; 177.06862; 194.07216; 205.06406; 211.07594; 222.06624 | GNPS | KYVJVURXKAZJRK-LBPRGKRZSA-N |
| 8 | Pallidine | 328.15444 | [M+H]^+^ | 153.06898; 163.08540; 165.06844; 168.05618; 177.06862; 178.08514; 205.06406; 211.07594; 227.07082; 239.07147; 251.06995; (297.11334) | GNPS | FBCNBECEGOCMPI-LIRRHRJNSA-N |
| 9 | Flavinantine | 328.15448 | [M+H]^+^ | 134.05945; 163.06190; 178.08514; 205.06113; 297.14505 | Proposed | GSNZKNRMDZYEAI-AUUYWEPGSA-N |
| 10 | Boldine | 328.15447 | [M+H]^+^ | 165.07108; 177.06862; 197.07216; 205.06406; 222.06624; 237.08978; (297.09924) | GNPS | LZJRNLRASBVRRX-ZDUSSCGKSA-N |
| 11 | Isoboldine | 328.15440 | [M+H]^+^ | 165.06844; 177.06862; 194.07216; 205.06406; 222.06624; 237.08978; (297.09924) | GNPS | LINHZVMHXABQLB-ZDUSSCGKSA-N |
| 12 | Corytuberine | 328.15438 | [M+H]^+^ | 165.06844; 178.07695; 189.07059; 191.08656; 193.06491; 194.07216; 205.06406; 222.06624; 239.07147 | GNPS | WHFUDAOCYRYAKQ-LBPRGKRZSA-N |
| 13 | Lauroscholtzine | 342.17014 | [M+H]^+^ | 165.07108; 179.08540; 194.07216; 207.08092; 210.06694; 222.06624; 237.09293; 265.08417 | GNPS | ZFLRVRLYWHNAEC-AWEZNQCLSA-N |
| 14 | Reticuline | 330.16918 | [M+H]^+^ | 115.05604; 137.05959; 143.04938; 175.07408; 177.07678; 192.10281; 299.12604 | MoNA | BHLYRWXGMIUIHG-HNNXBMFYSA-N |
| 15 | Armepavine | 314.17505 | [M+H]^+^ | 103.05588; 107.04881; 115.05384; 121.06413; 131.04800; 151.07916; 237.09293 | GNPS | ZBKFZIUKXTWQTP-QGZVFWFLSA-N |
| 16 | Zenkerine | 298.14367 | [M+H]^+^ | 152.06110; 165.06844; 178.07695; 189.07059; 207.08092; 251.06995; (281.14987) | GNPS | RELZHBBKERFUAJ-CQSZACIVSA-N |
| 17 | Tuduranine | 298.14343 | [M+H]^+^ | 152.06110; 165.06844; 178.07695; 189.07059; 207.06621; 235.05991; 281.11902 | Proposed | KUECBJOPWMRHEX-CQSZACIVSA-N |
| 18 | Diospiriofoline | 326.13859 | [M+H]^+^ | 221.0586; 237.0898; 249.0552; 265.0875; 279.0586; 295.0969 | Proposed | * CO[C@@H]1[C@@]2([H])N(CCc3c2c (c4c1cccc4O)c5c(OCO5)c3)C |
| 19 | Thaliporphine | 342.16985 | [M+H]^+^ | 152.06110; 165.07108; 178.07695; 189.07059; 193.06491; 205.06406; 207.08092; 221.05864; 251.10883 | GNPS | SAERKXUSZPTMCQ-UHFFFAOYSA-N |
| 20 | Predicentrine | 342.17007 | [M+H]^+^ | 220.08670; 251.10559; 279.10300; 296.10599; 311.12857 | MoNA | OUTYMWDDJORZOH-AWEZNQCLSA-N |
| 21 | Nuciferine | 296.16469 | [M+H]^+^ | 165.06844; 178.07695; 179.08540; 189.07059; 190.07584; 191.08656; 207.08864; 219.08057; (265.12079) | GNPS | ORJVQPIHKOARKV-OAHLLOKOSA-N |
| 22 | Corydine | 342.17042 | [M+H]^+^ | 165.06844; 179.08540; 193.06491; 207.08092; 219.08057; 221.06168; 238.06065; 247.07423; 253.08632; 265.08417 | GNPS | IDQUPXZJURZAGF-ZDUSSCGKSA-N |
| 23 | Domesticine | 326.13834 | [M+H]^+^ | 151.05402; 165.07108; 177.06862; 189.07059; 205.06406; 233.06061; 235.07558 | GNPS | ZMNSHBTYBQNBPV-ZDUSSCGKSA-N |
| 24 | Dehydrodicentrine | 338.13891 | [M+H]^+^ | 165.07108; 177.06862; 205.06406; 235.07558; 263.07019; 295.09686; | Proposed | * COc1cc2cc3N(C)CCc4cc5OCOc5c(c2cc1OC)c34 |
| 25 | Norisocorydine | 328.15421 | [M+H]^+^ | 152.06110; 165.06844; 178.07695; 189.06778; 191.08656; 207.07799; 219.08057; 221.05864; 236.08318; 238.06381; 247.07423; 253.08632; 265.08417; (311.12857) | GNPS | OHDQLTAYHMLRBA-LBPRGKRZSA-N |
| 26 | Laurotetanine | 328.15429 | [M+H]^+^ | 165.06844; 182.06990; 194.07216; 205.06406; 207.07799; 222.06624; 225.09052; 237.08978; 253.08632; 265.08417; 281.08130; (311.12857) | GNPS | GVVXPMORGFYVOO-ZDUSSCGKSA-N |
| 27 | Nordicentrine | 326.13862 | [M+H]^+^ | 152.06110; 165.06844; 179.08267; 189.07059; 205.06406; 207.08092; 221.05864; 235.07558; 263.07019 | GNPS | YNWJEUJZYKLCJG-ZDUSSCGKSA-N |
| 28 | Nornantenine | 326.13864 | [M+H]^+^ | 165.06844; 177.06862; 189.07059; 205.06406; 223.07613; 235.07558; 251.06995; 263.07019; 279.06543 | GNPS | JWXKBCGJLCEZTJ-UHFFFAOYSA-N |
| 29 | Nornuciferine | 282.14870 | [M+H]^+^ | 152.06110; 165.06844; 178.07695; 179.08540; 189.07059; 191.08656; 202.07614; 207.08092; 235.07558; (265.12411) | GNPS | QQKAHDMMPBQDAC-AWEZNQCLSA-N |
| 30 | Lirinidine | 282.14850 | [M+H]^+^ | 141.06770; 152.06110; 165.06844; 178.07695; 179.08540; 189.07059; 190.07584; 191.08656; 207.08092; 218.07373; (251.10559) | GNPS | YXVXMURDCBMPRH-AWEZNQCLSA-N |
| 31 | Glaucine | 356.18513 | [M+H]^+^ | 165.06844; 178.07695; 191.08374; 208.08569; 220.05029; 236.08318; 251.06670; 267.10580; 279.09958; 295.09686 | GNPS | RUZIUYOSRDWYQF-HNNXBMFYSA-N |
| 32 | Roemerine | 280.13274 | [M+H]^+^ | 152.06110; 165.07108; 178.07695; 189.07059; 190.07584; 191.08656; 201.07150; 218.07071 | GNPS | JCTYWRARKVGOBK-CQSZACIVSA-N |
| 33 | Nantenine | 340.15406 | [M+H]^+^ | 165.06844; 177.06862; 205.06406; 223.07307; 235.07558; 263.07019; 278.09259; 294.08945; 309.11176 | MoNA | WSVWKHTVFGTTKJ-AWEZNQCLSA-N |
| 34 | Dicentrine | 340.15426 | [M+H]^+^ | 251.06670; 278.09601; 279.06543; 280.06732; 309.11176 | GNPS | YJWBWQWUHVXPNC-AWEZNQCLSA-N |
| 35 | Dehydronuciferine | 294.14903 | [M+H]^+^ | 165.06844; 193.09901; 207.07799; 218.07071; 219.07755; 235.07558; 249.09068; 263.07019 | Proposed | JBGSWIBJAGBGOP-UHFFFAOYSA-N |
| 36 | Dicentrinone | 336.08626 | [M+H]^+^ | 206.05804; 234.05450; 263.05695; 275.05881; 292.06232; 320.05484 | GNPS | NEQVOBXBOFZEMR-UHFFFAOYSA-N |
| 37 | Leucoxylonine | 400.17563 | [M+H]^+^ | 237.08978; 265.08749; 293.08026; 323.09140; 338.11688; 369.13336 | Proposed | SFHWHWVEDBDXLV-AWEZNQCLSA-N |
| 38 | Stephenanthrine | 294.14881 | [M+H]^+^ | 165.06844; 178.07695; 189.07059; 191.08374; 201.06860; 219.08057; 249.09068 | Proposed | FXTBDJZGDJJCQU-UHFFFAOYSA-N |
| 39 | Argentinine | 296.16515 | [M+H]^+^ | 152.06110; 165.06844; 179.08540; 189.07059; 190.07584; 191.08656; 208.08864; 217.06319 | GNPS | HCXNUWJYBNHDAE-UHFFFAOYSA-N |
| 40 | Thalicthuberine | 354.16966 | [M+H]^+^ | 165.07108; 177.05501; 189.06778; 205.06406; 223.07307; 233.06061; 235.07558; 251.06995; 278.09259; 279.06543; 294.08945 | GNPS | DDCILWXYWBKXKC-UHFFFAOYSA-N |
| 41 | Discretamine | 328.15342 | [M+H]^+^ | 135.05959; 146.05649; 151.07413; 163.06190; 178.08514; 207.06621 | GNPS | KNWVMRVOBAFFMH-UHFFFAOYSA-N |
| 42 | Sesamin | 355.11784 | [M+H]^+^ | 91.05564; 197.06081; 247.07423; 355.11987 | MoNA | PEYUIKBAABKQKQ-AFHBHXEDSA-N |
| 43 | Ocophylol B | 359.18565 | [M+H]^+^ | 123.04333; 151.07413; 165.08946; 177.09039; 189.09027; 229.12169 | Proposed | * COc1cc(cc(OC)c1OC)c2oc3c(OC)cc(C=O)cc 3c2C |
| 44 | Eusiderin | 387.17956 | [M+H]^+^ | 151.07413; 165.05530; 177.05501; 189.08746; | Proposed | * COc1c(OC)c(OC)cc([C@H]2Oc3c (O[C@@H]2C)c(OC)cc(CC=C)c3)c1 |
| 45 | Licarin B | 325.14306 | [M+H]^+^ | 107.04881; 135.04391; 143.08607; 151.07413; 162.06853; 163.07495; 203.10658 | Proposed | DMMQXURQRMNSBM-YZAYTREXSA-N |
| 46 | Licarin A | 327.15865 | [M+H]^+^ | 137.05719; 143.08607; 151.07413; 165.06844; 178.07695; 171.08144; 188.04839; 193.09901; 203.06870; 221.09512; 295.13553 | MoNA | ITDOFWOJEDZPCF-FNINDUDTSA-N |
| 47 | Armenin B | 373.16390 | [M+H]^+^ | 153.09177; 165.05855; 179.03342; 203.07161; 229.08763; 241.08679; 257.08096; 341.13953; 379.17303 | Proposed | SSPDVRMNHFFRCE-JDEKTIPCSA-N |
| 48 | Quinic acid | 191.05433 | [M-H]^-^ | 85.02814; 93.03156; 127.03786; 137.02254; 171.02654; 173.04324; 191.05431 | MoNA | AAWZDTNXLSGCEK-LNVDRNJUSA-N |
| 49 | Taxifolin | 303.04983 | [M-H]^-^ | 125.02214; 137.02254; 153.01779; 175.03928; 217.04663; 241.04916; 259.05661; 275.05774; 285.03790 | GNPS | CXQWRCVTCMQVQX-LSDHHAIUSA-N |
| 50 | Catechin / Epicatechin | 289.07091 | [M-H]^-^ | 93.0156; 109.02729; 123.04298; 137.02254; 159.04279; 187.03732 | MoNA | PFTAWBLQPZVEMU-DZGCQCFKSA-N / PFTAWBLQPZVEMU-UKRRQHHQSA-N |
| 51 | Isoquercitrin | 463.08689 | [M-H]^-^ | 151.00093; 243.02718; 255.02904; 271.02179; 300.02765; 463.0877 | MoNA | OVSQVDMCBVZWGM-QSOFNFLRSA-N |
| 52 | Vitexin-2'-O-rhamnoside | 577.15701 | [M-H]^-^ | 293.04349; 311.05334; 341.06476; 413.08524; 457.11850 | GNPS | LYGPBZVKGHHTIE-HUBYJIGHSA-N |
| 53 | Rutin | 609.14699 | [M-H]^-^ | 151.00345; 178.99718; 243.02718; 255.02904; 271.02179; 300.02408; 301.03207 | GNPS | IKGXIBQEEMLURG-NVPNHPEKSA-N |
| 54 | Quercimeritrin | 463.08762 | [M-H]^-^ | 199.04004; 227.03430; 243.02718; 255.02904; 271.02515; 289.07208; 300.02765; 301.03207; 463.08667 | Proposed | BBFYUPYFXSSMNV-HMGRVEAOSA-N |
| 55 | Vitexin | 431.09731 | [M-H]^-^ | 269.04352; 283.06070; 311.05695;323.05396; 341.06476 | GNPS | SGEWCQFRYRRZDC-VPRICQMDSA-N |
| 56 | Quercitrin | 447.09299 | [M-H]^-^ | 151.00345; 227.03430; 243.02718; 255.02904; 271.02515; 300.02765; 447.09302 | GNPS | OXGUCUVFOIWWQJ-HQBVPOQASA-N |
| 57 | Reynoutrin | 433.07686 | [M-H]^-^ | 151.00093; 199.04004; 227.03122; 227.03122; 243.02718; 255.02904; 271.02179; 300.02408 | MoNA | PZZRDJXEMZMZFD-BWYUNELBSA-N |
| 58 | Astragalin | 447.09298 | [M-H]^-^ | 183.04088; 227.03430; 255.02904; 284.03290 | MoNA | JPUKWEQWGBDDQB-QSOFNFLRSA-N |
| 59 | Apigenin-7-O-rutinoside | 577.15675 | [M-H]^-^ | 117.03406; 268.03702; 269.0475 | MoNA | FKIYLTVJPDLUDL-SLNHTJRHSA-N |
| 60 | Schaftoside / Isoschaftoside | 563.14164 | [M-H]^-^ | 353.06760; 383.08264; 413.08524; 443.09760; 473.10629; 563.14203 | GNPS | MMDUKUSNQNWVET-VYUBKLCTSA-N / OVMFOVNOXASTPA-VYUBKLCTSA-N |
| 61 | Afzelin | 431.09706 | [M-H]^-^ | 107.01432; 183.04364; 211.03810; 227.03430; 255.02904; 284.02945; 285.0238 | MoNA | SOSLMHZOJATCCP-AEIZVZFYSA-N |
| 62 | Kaempferol 3-4''-p-coumarylrhamnoside | 593.12992 | [M-H]^-^ | 163.0387; 285.0379; 291.0841; | Proposed | RFTKNPGPPJOOBI-NSPOHDSESA-N |
| 63 | Quercetin | 301.03286 | [M-H]^-^ | 107.01220; 121.02779; 151.00093; 227.03430; 243.02718; 271.02179; 301.03207 | MoNA | REFJWTPEDVJJIY-UHFFFAOYSA-N |
| 64 | Apigenin | 269.04407 | [M-H]^-^ | 117.03185; 149.02243; 151.00093; 201.05260; 225.05504; 227.03430; 269.04352 | GNPS | KZNIFHPLKGYRTM-UHFFFAOYSA-N |
| 65 | Kaempferol | 285.03873 | [M-H]^-^ | 143.04767; 159.04279; 171.04260; 187.03732; 201.05550; 211.03810; 227.03430; 239.05389; 285.03790 | GNPS | IYRMWMYZSQPJKC-UHFFFAOYSA-N |
| 66 | Kaempferol 3-(2'',4''-  di-(E)-*p-*coumaryl-rhamnoside) / Kaempferol 3-(3'',4''-  di-(E)-*p-*coumaryl-rhamnoside) | 723.17282 | [M-H]^-^ | 145.02815; 187.03732; 229.05013; 255.02904; 284.03290; 285.03790; 437.12308; 577.13794; 723.17230 | GNPS | KMOHJUXDKSMQOG-OLHCXIDTSA-N |

**^#^**Fragment ions between brackets were not found in the reference spectra but were proposed based on chemical knowledge of MS fragmentation and observed MS^E^ spectra. *SMILES is provided, once referred Inchkey is not available.

**Table S2 | Metadata for *Ocotea* plant samples.** Including collection details, species information, location, geographical coordinates, related activity, and references. Provides contextual information abou t analyzed samples to enable biological interpretation of metabolomics data.

| **Code** | **Date** | **Specie** | **Popular name** | **Synonym** | **Location** | **Geographical location** | **Related activity** | **Reference** |
| --- | --- | --- | --- | --- | --- | --- | --- | --- |
| AM | 1935 | *Ocotea amazonica (Meiss) Mez* | unknown | X | Fazenda da cachoeira, Tombos, Minas Gerais, Brazil (Jardim Botânico de Belo Horizonte) | - | No reported activity | x |
| AU | 2013 | *Ocotea acutifolia (Nees) Mez* | canela-branca | X | Santa Rosa, Ruta Nacional 118, Km 72, Concepción, Corrientes, Argentina | 72°28′18.0″S 58°08′21.0″ W | Cytotoxic activity against human cancer cell lines and mutagenic and genotoxic effects on wing cells of *Drosophila melanogaster* | Garcez *et al.* (2011) and Guterres *et al*. (2013) |
| AY | 2005 | *Ocotea aciphylla (Nees & Mart.) Mez* | canela-amarela | X | Serra das Almas - Inácio Pinto, Sítio Gaia da Mata, Rio de Contas, Bahia, Brazil | 13°32′14.0″S 41°54′14.0″ W | *In vitro* inhibitory activity over AChE and acaricidal activity against *Rhipicephalus (Boophilus) microplus* | Carneiro *et al.* (2018) and Conceição *et al.* (2017) |
| BA | 2004 | *Ocotea brachybotrya (Meisn.) Mez* | louro-verdadeiro | x | Paque estadual do vale do rio doce, Timóteo, Minas Gerais, Brazil (ICB-UFMG) | 19°35′28.0″S 42°34'′07.0″ W | No reported activity | x |
| BI | 2005 | *Ocotea bicolor Vattimo-Gil* | canela-preta | x | Parque Estadual de Ibitipoca, Floresta no acero do parque, Lima Duarte, Minas Gerais, Brazil | - | *In vitro* antioxidant activity | Damasceno *et al.* (2017) |
| BR | 2001 | *Ocotea bragai Coe-Teix.* | unknown | x | Parque Estadual da Cantareira, Região das águas, Mariporã, São Paulo, Brazil | - | No reported activity | x |
| CA | 2007 | *Ocotea caesia Mez* | unknown | x | Serra de Antônio Pereira, Samarco. Alegria 7, Ouro Preto, Minas Gerais, Brazil | - | No reported activity | x |
| CE | 2003 | *Ocotea cernua (Nees) Mez* | moena negra | x | Morro do Imperador, Juiz de Fora, Brazil | - | No reported activity | x |
| CJ | 2003 | *Ocotea cujumary Mart.* | cuchumari | x | APA logoa Silvana, Caratinga, Minas Gerais, Brazil | - | *In vitro* antibacterial and cytotoxic activities against *E. coli* and MCF-7 cells, respectively | Da Silva *et al.* (2017) |
| CL | 1974 | *Ocotea calliscypha L.C.S.Assis & Mello-Silva* | unknown | x | Serra do Frazão, Ouro Preto, Minas Gerais, Brazil | 20°17′15.0″S 43°30′19.1″ W | No reported activity | x |
| CM | 2003 | *Ocotea complicata (Meisn.) Mez* | unknown | x | Rodovia BA-001, Una, Bahia, Brazil (Prefeitura de Curitiba) | - | *In vitro* leishmanicidal activity | Rebouças-Silva *et al.* (2023) |
| CO | 2002 | *Ocotea corymbosa (Meisn.)Mez* | canela-fedida | x | Eldorado, Mato Grosso do Sul, Brazil | - | No reported activity | x |
| CT | 2002 | *Ocotea catharinensis Mez* | canela-coqueiro | x | mata atlântica | - | No reported activity | x |
| DI | 1993 | *Ocotea dispersa (Nees & Mart.) Mez* | canela-sassafrás | x | Serra do Itacolomi, Ouro Preto, Minas Gerais, Brazil | - | *In vitro* antileishmanial activity | Alcoba *et al*. (2017) |
| DO | 2004 | *Ocotea diospyrifolia (Meisn.) Mez* | canela-louro | x | Mata do Baú, Barroso, Minas Gerais, Brazil | - | *In vivo* anti-inflammatory activity and *in vitro* antibacterial activity against *Salmonella spp.* | Silva *et al*. (2021) and Weber *et al*. (2018) |
| DV | 2003 | *Ocotea divaricata (Nees) Mez* | canela-segueira | x | Reserva Biológica da Represa do Grama, Descoberto, Minas Gerais, Brazil | - | No reported activity | x |
| EL | 2001 | *Ocotea elegans Mez* | canela-broto | *Ocotea indecora (Schott) Mez* | Estrada para São Mateus, Camanducaia, Minas Gerais, Brazil (ICB-UFMG) | - | *In vitro* acaricidal and repellent activity on *Rhipicephalus (Boophilus) microplus* and insecticidal activity against *Dysdercus peruvianus* with AChE inhibition | Figueiredo *et al.* (2018) and Nascimento *et al.* (2020) |
| FE | 1999 | *Ocotea felix Coe-Teix.* | unknown | x | Parque Estadual do Itacolomi, Ouro Preto, Minas Gerais, Brazil | - | No reported activity | x |
| GA | 1997 | *Ocotea guianensis Aubl.* | canela-seda | x | Margem do Rio Xingú, São Jose do Xingú, Mato Grosso, Brazil | - | No reported activity | x |
| GL | 1971 | *Ocotea glauca (Nees & Mart.) Mez* | louro | x | Santa Rita Durão, Mariana, Minas Gerais, Brazil | 20°22′40.0″S 43°24′57.9″ W | No reported activity | x |
| GU | 2001 | *Ocotea glaucina (Meisn.) Mez* | unknown | *Ocotea notata (Nees & Mart.) Mez* | Estrada Grão Mongol-Cristália KM 6, Grão Mongol, Minas Gerais, Brazil (USP) | 16º35′'47.0″S 42º54′05.0″ W | *In vitro* antiviral activity against *Herpes simplex* virus types 1 and 2*,* antioxidant activity, *in vitro* anti-mycobacterial and immunomodulatory activities and *in vitro* antifungal activity against *Sporothrix brasiliensis* | Garrett *et al.* (2012), Pereira *et al*. (2019), Costa *et al.* (2015 and 2021) and de Souza *et al.* (2023) |
| GZ | 2006 | *Ocotea glaziovii Mez* | canela-amarela* | x | Mananciais da Serra, Represa do Carvalinho, FOM/FODM, Piraquara, Paraná, Brazil | - | Anxiolytic and antiviral | x |
| HY | 1993 | *Ocotea hypoglauca (Nees & Mart.) Mez* | unknown | x | Parque Estadual do Itacolomi, Estrada para a Fazenda do Manso, Ouro Preto, Minas Gerais, Brazil | - | No reported activity | x |
| IN | 2001 | *Ocotea indecora (Schott) Mez* | canela | *Ocotea elegans Mez* | Reserva Biológica da Represa do Grama, Descoberto, Minas Gerais, Brazil | - | *In vitro* larvicidal effect against *Aedes aegypti* larvae | Machado *et al.* (2023) |
| KU | 1979 | *Ocotea kuhlmannii Vattimo-Gi/Ocotea nectandrifolia Mez* | canela-burra | *Ocotea nectandrifolia Mez* | Ilha de Santa Catarina - Lagoa do Peri, Altitude: 300m., Florianópolis, Santa Catarina, Brazil | - | Antifungal-*Candida sp* | x |
| LA | 2010 | *Ocotea lanata (Nees & Mart.) Mez* | unknown | x | Rancho das Tábuas, Angelina, Paraná, Brazil (FURB) | 27°37′49.0″S 49°02′58.0″ W | No reported activity | x |
| LC | 2003 | *Ocotea lancifolia (Schott) Mez* | canela-sabão | *Ocotea lanceolata (Nees) Nees; Ocotea variabilis Mart.* | Parque do São Gonçalo do Rio Preto, São Gonçalo do Rio Preto, Minas Gerais, Brazil (ICB-UFMG) | 18°06′54.0″S 43°20′28.0″ W | *In vitro* acaricidal activity on *Rhipicephalus (Boophilus) microplus, in vitro* antifungal activity against *Fusarium moniliforme(a), Trametes versicolor* and *Gloeophyllum trabeum (b), in vitro* antioxidant *activity* and *in vitro* antiprotozoal activities against *Leishmania spp. a*nd *Trypanossoma cruzi* | Barbosa *et al.* (2013), da Silva *et al.* (2018) *(a)*and (2017) *(b),* and Fournet *et al*. (2007) |
| LF | 1992 | *Ocotea longifolia Kunth* | louro-ingá | x | Alto do Galo, Domingos Martins, Espírito Santo, Brazil | - | *In vitro* cytotoxic activities against cancer cell lines HepG2 and HL60 and insecticide activity against *Sitophilus zeamais* | da Silva *et al.* (2016) and Prieto *et al.* (2010) |
| LG | 1938 | *Ocotea langsdorffii (Meisn.) Mez* | unknown | x | Serra do Cipó Minas Gerais, Brazil | 19°15′30.0″S 43°33′04.0″ W | No reported activity | x |
| LN | 2009 | *Ocotea lanceolata (Nees) Nees* | canela-pilosa | *Ocotea lancifolia (Schott) Mez* | Parque Nacional do Iguaçu, Foz do Iguaçu, Paraná, Brazil (Prefeitura Municipal de Curitiba) | 25°32′52.0″S 54°35′17.1″ W | *In vitro* acaricidal activity on *Rhipicephalus (Boophilus) microplus, in vitro* antifungal activity against *Fusarium moniliforme(a), Trametes versicolor* and *Gloeophyllum trabeum (b), in vitro* antioxidant *activity* and *in vitro* antiprotozoal activities against *Leishmania spp. a*nd *Trypanossoma cruzi* | Barbosa *et al.* (2013), da Silva *et al.* (2018) *(a)*and (2017) *(b),* and Fournet *et al*. (2007) |
| LO | 2003 | *Ocotea lobbii (Meisn.) Rohwer* | unknown | x | Canavieiras, Bahia, Brazil (Prefeitura Municipal de Curitiba) | 22°05′21.1″S 43°49′40.0″ W | No reported activity | x |
| LX | 1997 | *Ocotea laxa (Nees) Mez* | canela-pimenta | x | Parque Estadual do Itacolomi, Córrego do Belchior, , Ouro Preto, Minas Gerais, Brazil | 20°17′15.0″S 43°30′19.0″ W | No reported activity | x |
| MI | 2003 | *Ocotea minarum (Nees & Mart.) Mez* | canela-vassoura | x | Fazenda Renascença, Bonito, Mato grosso do Sul, Brazil |  | *In vitro* antioxidant activity | Rodrigues *et al.* (2019) |
| MU | 2007 | *Ocotea nummularia* | canelinha | x | Serra da Piedade, Caeté, Minas Gerais, Brazil | 19°52′47.9″S 43°40′10.9″ W | No reported activity | x |
| NE | 2010 | *Ocotea nectandrifolia Mez* | canela-burra | x | Anta Branca (antigo Alto Rio do Oeste), Rio do Campo, Santa Catarina, Brazil (FURB) | 26°54′36.0″S 50°13′13.0″ W | No reported activity | x |
| NI | 2003 | *Ocotea nitida (Meisn.) Rohwer* | louro* | x | Morro do Gavião, Dionísio, Minas Gerais, Brazil | 19°50′03.0″S 42°33′07.0″ W | No reported activity | x |
| NO | 2010 | *Ocotea notata (Nees & Mart.) Mez* | louro-pipoca | *Ocotea glaucina (Meisn.) Mez* | Fazenda Lucuri, Serra do Curral Frio, Umburanas, Bahia, Brazil | 10°43′58.0″S 41°19′35.0″ W | *In vitro* antiviral activity against *Herpes simplex* virus types 1 and 2*,* antioxidant activity, *in vitro* anti-mycobacterial and immunomodulatory activities and *in vitro* antifungal activity against *Sporothrix brasiliensis* | Garrett *et al.* (2012), Pereira *et al*. (2019), Costa *et al.* (2015 and 2021) and de Souza *et al*. (2023) |
| NT | 2008 | *Ocotea nitidula (Nees et Mart. ex Ness)* | unknown | x | Parque Estadual do Itacolomi, Ouro Preto, Minas Gerais, | - | No reported activity | x |
| NU | 2009 | *Ocotea nutans (Nees) Mez* | unknown | x | Brazil, Minas Gerais, Mariana, Parque Estadual do Itacolomi | 20°17′15.0″S 43°30′19.0″ W | *In vitro* activity against *Aedes aegypt*i larvae and antioxidant activity | Betim *et al*. (2019) and (2021) |
| OD | 2004 | *Ocotea odorifera Vell. Rohwer* | canela-sassafrás | *Ocotea pretiosa (Nees) Mez* | Reserva Biológica da Represa do Grama, Descoberto, Minas Gerais, Brazil | - | *In vivo* anti-inflammatory activity, *in vitro* antibacterial activity against *Staphylococcus aureus* (*a, b* and *c*), *in vitro* antioxidant and antimutagenic activities (*a*), in vitro antifungal activity against *Candida parapsilosis, in vitro* antileishmanial activity and insecticidal and repellent activities against *Sitophilus zeamais* | Alcantara *et al*. (2021), Gontijo *et al*.(2017) (*a*), de Almeida *et al*.(2020) (*b*) and (2022) (*c*), Yamaguchi *et al*.(2010), Alcoba *et al*. (2017) and Mossi *et al*. (2013) |
| PA | 2011 | *Ocotea paranaenses Brotto, Baitello, Cervi & E.P.Santos* | unknown | x | Morro dos Perdidos, Serra da Araçatuba, Guaratuba, Paraná, Brazil | 25°52′58.0″S 48°34′28.9″ W | *In vitro* antibacterial activity against *Staphylococcus aureus* and antioxidant activity | Gribner *et al.* (2022) |
| PC | 2008 | *Ocotea pulchella (Nees & Mart.) Mez* | canela-lageana | x | Pedra Branca, Pocinhos do Rio Verde, Caldas, Minas Gerais, Brazil | 21°55′24.9″S 46°23′09.9″ W | *In vitro* antifungal activity against *Sporothrix brasiliensis, in vitro* antioxidant activity, in vitro antiviral activity against SuHV-1, and molluscicidal and antiparasitic effects on Biomphalaria glabrata and *Schistossoma mansoni*, respectively | de Souza et al. (2023), Reboucas et al. (2015), Padilla et al. (2018) and Passos et al. (2020) |
| PE | 1998 | *Ocotea percoriacea Kosterm.* | unknown | x | Cachoeira das Androinhas, Estrada à esquerda, Ouro Preto, Minas Gerais, Brazil | 20°17′15.0″S 43°30′19.1″ W | In vitro inhibitory activity over AChE | Cassiano *et al*. (2019) |
| PH | 2010 | *Ocotea pulchraVattimo-Gil* | canela | x | Rio Veado, Nova Trento, Santa Catarina, Brazil | 27°21′38.0″S 49°08′13.0″ W | No reported activity | x |
| PL | 2006 | *Ocotea pulchea Vattimo-Gil* | unknown | x | Reserva Biológica Municipal Santa Cândida, Juiz de Fora, Minas Gerais, Brazil | - | No reported activity | x |
| PO | 1906 | *Ocotea pomaderroides (Meisn.) Mez* | canela | x | Miguel Burneir, Ouro Preto, Minas Gerais, Brazil | - | In vitro inhibitory activity over AChE | Reis *et a*l. (2022) |
| PR | 1995 | *Ocotea porosa (Nees & Mart.) Barroso* | imbuia | x | Buraco do Padre, Ponta Grossa, Paraná (Prefeitura Municipal de Curitiba) | - | *In vitro* antibacterial activity against *Staphylococcus aureus* and *in vitro cytotoxic effects on* McCoy, B16F10 and MCF7 cell lines | Brito (2009) and Brutulim *et al*. (2020) |
| PT | 1994 | *Ocotea pretiosa (Nees) Mez* | canela-sassafrás | *Ocotea odorifera Vell. Rohwer* | Mata do Morro Redentor, Juiz de Fora, Minas Gerais, Brazil | - | *In vivo* anti-inflammatory activity, *in vitro* antibacterial activity against *Staphylococcus aureus* (*a, b* and *c*), *in vitro* antioxidant and antimutagenic activities (*a*), in vitro antifungal activity against *Candida parapsilosis, in vitro* antileishmanial activity and insecticidal and repellent activities against *Sitophilus zeamais* | Alcântara *et al*. (2021), Gontijo *et al*.(2017) (*a*), de Almeida *et al*.(2020) (*b*) and (2022) (*c*), Yamaguchi *et al*.(2010), Alcoba *et al*. (2017) and Mossi *et al*. (2013) |
| PU | 2008 | *Ocotea puberula (Rich.) Nees* | canela-babosa | x | Parque Estadual do Itacolomi, Ouro Preto, Minas Gerais, | 20°17′15.0″S 43°30′19.1″ W | *In vitro* activity against *Trypanosoma cruzi, in vivo* activities of wound healing and antinociceptive activity in murine models | Barbosa *et al.* (2020) and (2021), Arcaro *et al*. (2023) and Montrucchio *et al*. (2012) |
| SP | 1994 | *Ocotea spectabilis (Meisn.) Mez* | canela-baraúna | x | Mariana, Minas Gerais, Brazil | 20°22′40.0″S 43°24′57.9″ W | No reported activity | x |
| SX | 1994 | *Ocotea spixiana (Nees) Mez* | canelão | x | Parque Estadual do Itacolomi, Estrada para a Fazenda do Manso, Ouro Preto, Minas Gerais, Brazil | 20°17′15.0″S 43°30′19.1″ W | *In vitro* acaricidal activity against Rhipicephalus (Boophilus) microplus | Conceição *et al*. (2020) |
| TA | 2007 | *Ocotea tabacifolia (Meisn.) Rohwer* | unknown | x | Serra de Antônio Pereira, Samarco. Alegria 7, Ouro Preto, Minas Gerais, Brazil | - | No reported activity | x |
| TE | 2001 | *Ocotea tenuiflora (Nees) Mez* | unknown | x | Brazil, Minas Gerais, Descoberto, Reserva Biológica da Represa do grama | - | No reported activity |  |
| TL | 2001 | *Ocotea teleiandra (Meisn.) Mez* | canela-limão | x | Reserva Biológica da Represa do Grama, Descoberto, Minas Gerais, Brazil | - | No reported activity | x |
| TR | 1997 | *Ocotea tristis (Nees & Mart.) Mez* | canelinha | x | Antônio Pereira, Ouro Preto, Minas Gerais, Brazil | 20°17′15.0″S 43°30′29.1″ W | No reported activity | x |
| VA | 1977 | *Ocotea vaccinioides (Meisn.) Mez* | unknown | *Ocotea daphnifolia (Meisn.) Mez* | Rancharia, Ouro Preto, Minas Gerais, Brazil | - | No reported activity | x |
| VI | 2008 | *Ocotea villosa Kosterm.* | unknown | x | Sítio Malícia, Mata do Krambeck, Juiz de Fora, Minas Gerais, Brazil | - | No reported activity | x |
| VL | 1994 | *Ocotea velutina (Nees) Rohwer* | canelão-amarelo | x | Parque Estadual das Lauraceaes, Bocaiúva do sul, Paraná, Brazil | - | No reported activity | x |
| VR | 1967 | *Ocotea variabilis Mart* | canela-pilosa | *Ocotea lancifolia (Schott) Mez* | Estrada de Campo Alegre para Araguaí, Campo Alegre, Minas Gerais, Brazil | - | *In vitro* acaricidal activity on *Rhipicephalus (Boophilus) microplus, in vitro* antifungal activity against *Fusarium moniliforme(a), Trametes versicolor* and *Gloeophyllum trabeum (b), in vitro* antioxidant */* antiprotozoal activities against *Leishmania spp. a*nd *T. cruzi* | Barbosa *et al.* (2013), da Silva *et al.* (2018) *(a)*and (2017) *(b),* and Fournet *et al*. (2007) |
| VZ I | 2000 | *Ocotea velloziana (Meisn.) Mez* | canela-verde | x | Camarinhas, Ouro Preto, Minas Gerais, Brazil | - | *In vitro* larvicidal activity against Aedes aegypti larvae | Garcez *et al*. (2009) |

**Table S3 | Manual annotation chemical standards from Dataset 2 with level 2 of confidence on the final data processing list.** Chemical standards were detected and identified by matching accurate mass, isotopic pattern, and fragmentation spectra to public databases literature sources, and chemical knowledge. Name, exact mass, observed *m/z,* RT*,* adducts, detected in high or low concentration in processed .mzML data, main MS/MS fragments, and supporting international identifier (InChIKey) are provided.

| ID | Name | Exact mass | Observed *m/z* | RT | Adduct | High (H) or low (L) concentration | MS/MS fragments | InChIKey |
| --- | --- | --- | --- | --- | --- | --- | --- | --- |
| 1a | Vancomycin | 1447.4302 | 724.72009 | 0.50 | [M+2H]^2+^ | H | 100.07672; 144.10208; 329.07394; 800.10370; 1087.28760; 1115.28979; 1143.27893 | MYPYJXKWCTUITO-LYRMYLQWSA-N |
| 2a | Nystatin | 925.50350 | 926.51166 | 2.89 | [M+H]^+^ | H / L | 161.13705; 279.12503; 297.13632; 339.14346; 655.36560; 673.36810; 691.38489; 727.40198; 745.41968; 890.48761; 908.48932 | VQOXZBDYSJBXMA-RKEBNKJGSA-N |
| 3a | Azithromycin | 748.50853 | 749.51324 | 2.10 | [M+H]^+^ | H | 72.0827; 98.09959; 116.10944; 158.12018; 186.14938; 257.08594; 398.29184; 434.31146; 573.41479; 591.41864 | MQTOSJVFKKJCRP-BICOPXKESA-N |
| 4a | Novobiocin | 612.23191 | 613.24133 | 4.35 | [M+H]^+^ | H / L | 97.06465; 133.03056; 186.07791; 189.09122; 218.10526; 345.01031; 396.14288 | YJQPYGGHQPGBLI-KGSXXDOSSA-N |
| 5a | Puromycin | 471.22302 | 472.23154 | 1.93 | [M+H]^+^ | H / L | 150.09512; 164.09294; 264.11874; 292.11609; 309.14447; 371. ; | RXWNCPJZOCPEPQ-NVWDDTSBSA-N |
| 6a | Betulinic acid | 456.36035 | 457.37149 | 5.55 | [M+H]^+^ | H | 95.08782; 163.14926; 191.17993; 231.20955; 249.05836; 297.08365; 393.35013; 411.36081; 439.35933 | QGJZLNKBHJESQX-FZFNOLFKSA-N |
| 7a | Oleanolic Acid | 456.36035 | 457.37149 | 5.67 | [M+H]^+^ | H / L | 95.08782; 163.14926; 191.17993; 221.08742; 231.20955; 269.98254; 297.08365; 393.35013; 411.36081; 439.35933 | MIJYXULNPSFWEK-GTOFXWBISA-N |
| 8a | Ursolic acid | 456.36035 | 457.37149 | 5.68 | [M+H]^+^ | H / L | 95.08782; 163.14926; 191.17993; 221.08742; 231.20955; 297.08365; 393.35013; 411.36081; 439.35933 | WCGUUGGRBIKTOS-GPOJBZKASA-N |
| **9a** | Tetracycline | 444.15327 | 445.15884 | 1.95 | [M+H]^+^ | H / L | 86.02639; 98.06068; 126.05542; 154.05173; 241.00900; 269.07956; 337.07254; 365.06461; 410.12222; 427.1499; 428.13821 | NWXMGUDVXFXRIG-WESIUVDSSA-N |
| **10a** | Doxycycline | 444.15327 | 445.15884 | 2.29 | [M+H]^+^ | H | 84.04588 ; 98.09527; 126.05542; 154.05179; 201.05211; 267.06400; 321.07751; 339.08719; 410.12222; 428.13821 | SGKRLCUYIXIAHR-AKNGSSGZSA-N |
| 11ª | Florfenicol | 357.00046 | 358.00943 | 2.52 | [M+H]^+^ | H / L | 132.06059; 170.06255; 206.04065; 208.03178; 210.05780; 241.00900; 243.00554; 319.99149; 339.00684; 341.00305 | AYIRNRDRBQJXIF-NXEZZACHSA-N |
| 12a | Thiamphenicol | 355.0048 | 356.01337 | 2.02 | [M+H]^+^ | H / L | 146.06334; 198.06124; 228.10692; 230.00967; 240.00029; 242.01303; 307.12915 ; 309.14447 ; 311.14328 | OTVAEFIXJLOWRX-NXEZZACHSA-N |
| **13a** | Mitomycin C | 334.12772 | 335.13580 | 1.89 | [M+H]^+^ | H / L | 77.03879; 104.05007; 131.06418; 132.07062; 169.07913; 171.09450; 215.08574; 242.09453 | NWIBSHFKIJFRCO-WUDYKRTCSA-N |
| 14a | Chloramphenicol | 322.01233 | 323.02032 | 2.63 | [M+H]^+^ | H / L | 132.06059; 165.06738; 206.03439; 241.00900; 243.00554; 257.99667; 259.99185; 274.99786; 276.9948; 305.0064; 307.00674 | WIIZWVCIJKGZOK-RKDXNWHRSA-N |
| 15a | Cycloheximide | 281.16271 | 282.16724 | 2.70 | [M+H]^+^ | H / L | 107.08649; 159. 12080; 187.10951; 219.13799 229.12338; 246.15276; 264.16132 | YPHMISFOHDHNIV-FSZOTQKASA-N |

**Table S4 Annotation with an enhanced level of confidence (level 2) of automated annotation with the level of confidence 3 using the *Actinomarine*DB.** Putative metabolites were annotated by matching accurate mass, isotopic pattern, and fragmentation spectra to public databases literature sources and chemical knowledge. Putative name, exact mass, observed *m/z,* RT*,* adducts, main MS/MS fragments, *in-house*DB, and supporting international identifier (InChIKey) are provided.

| ID | Putative metabolite name | Exact mass | Observed *m/z* | RT | Adduct | MS/MS fragments | Reference | *Actinomarine*DB hit | InChIKey |
| --- | --- | --- | --- | --- | --- | --- | --- | --- | --- |
| 1b | Fluostatin A | 306.0528 | 307.0603 | 3.16 | [M+H]^+^ | 236.04697; 251.06715; 263.06979; 279.06668; 281.0886 | GNPS | Yes | ISHOMJGAOPXCEF-  HFFFAOYSA-N |
| 2b | Fluostatin K | 308.0684 | 309.0754 | 3.22 | [M+H]^+^ | 263.06979; 281.07571; 291.06593 | Proposed | Yes | CKTLJJYDUPSLCZ-  SFHVURJKSA-N |
| 3b | Kinobscurinone | 306.0528 | 613.1116 | 4.32 | [2M+H]^+^ | 223.279.0666; 265.0489; 289.0502; 307.0603; | Proposed | Yes | CC1=CC(=C2C(=C1)C(=O)C3=C2C(=O)C4=C(C3=O)C(=CC=C4)O)O |
| 4b | Rosamicin | 581.3564 | 582.3661 | 2.27 | [M+H]^+^ | 98.0976; 113.05816; 116.10474; 158.12018 | Proposed | Yes | IUPCWCLVECYZRV-  ZMZINANSA-N |
| 5b | γ-Actinorhodin | 630.1009 | 631.1201 | 3.88 | [M+H]^+^ | 499.10809; 543.10413; 571.09656; 585.11890; 613.11163 | GNPS | No | * CC1OC(CC(O7)=O)C7C(C (C3=C2C(O)=C(C4=CC(O)=C(C (C(C(O8)C(CC8=O)OC6C)=C6C5  =O)=O)C5=C4O)C=C3O)=O)=  C1C2=O |
| 6b | Phomin | 479.2671 | 480.2815 | 3.11 | [M+H]^+^ | 81.07118; 91.05359; 105.06780; 107.08649; 119.08701; 145.01472; 444.25668 | GNPS | Yes | GBOGMAARMMDZGR-TYHYBEHESA-N |
| 7b | Desferrioxamine B | 560.3533 | 585.3195 | 1.36 | [M-2H+Al]^+^ | 70.06599; 241.11066; 300.10849; 303.19546; 368.17395; 385.20248; 467.2079 | GNPS | No | UBQYURCVBFRUQT-UHFFFAOYSA-N |

*SMILES are provided when InChIKey is not available.

**Supplementary Text (ST-1)**

**Alkaloids**

Forty-one alkaloids were level 2 annotated in the LC-HRMS QC ESI^+^ metabolic fingerprint. In the range of retention time at 0.5-1.5 min, the pyrrolidine alkaloid 4-hydroxy*-N*-methylproline (**1,** R_t_ 0.49 min) was annotated in high intensity with the parent ion at *m/z* 146.081. Subsequently, from 1.5 to 2.5 min, two proaporphines, a variety of noraporphines and aporphines and two benzylisoquinoline alkaloids were annotated. More specifically, from 1.4 to 1.54 min, the proaporphines were annotated as crotsparine (**2**, R_t_ 1.42 min) and glaziovine (**3,** R_t_ 1.49 min), at *m/z* 284.127 and *m/z* 298.144, respectively. The *N-*methylcoclaurine (**4**, R_t_ 1.54 min), a benzylisoquinoline, was annotated with parent ion at *m/z* 300.160, while the ion at *m/z* 298.144 was annotated as the aporphine 3-hydroxynornuciferine (**5**, R_t_ 1.60 min) (**Fig. 4; Table 1; Table S1**). Additionally, other two other noraporphine were annotated with parent ion at *m/z* 314.139, as the laurelliptine (**6**, R_t_ 1.66 min), and the laurolitsine (norboldine) (**7**, R_t_ 1.74 min). Furthermore, five main alkaloid isomers eluted at R_t_ 1.61 – 1.96 min displayed the same observed parent ion at *m/z* = 328.155. Throughout MS^E^ fragment analyses, compounds were putatively annotated as pallidine (**8,** R_t_ 1.61 min), flavinantine (**9**, R_t_ 1.75 min), which belongs to the morphinandienone alkaloid class that derived from the benzyltetrahydroisoquinoline core. In addition, the aporphine isomers boldine (**10**, R_t_ 1.85 min), isoboldine (**11**, R_t_ 1.96 min), and corytuberine (**12**, R_t_ 2.06 min) could be differentiated by their respective observed MS^E^ fragment ions. Moreover, the aporphine lauroscholtzine (*N*-methyllaurotetanine) was also annotated at *m/z* 342.170 (**13**, R_t_ 2.02 min) (**Fig. 4-5; Table 1; Table S1**).

Regarding the gas-phase fragmentation reactions involved in high energy channels on MS^E^/DIA mode to these isoquinoline alkaloids, several exhibited similar product ions. For example, for the noraporphines **6** and **7** and aporphines **10**, **11** and **12** the main difference between them lies in their respective spectra abundances and the first neutral losses they undergo, which are NH_3_ and NH_2_CH_3_, respectively. Consequently, these compounds share a common diagnostic ion at *m/z* 297.112. Successively, the presence of the fragment ion at *m/z* 265.085 (C_17_H_13_O_3_^+^) was attributed to the loss of CH_3_OH (32.03 Da). The next observed fragment ion at *m/z* 237.092 (C_16_H_13_O_2_^+^) is a result of a CO (27.99 Da) neutral loss from the *m/z* 265.085.

Moreover, the fragmentation pathway of proaporphine alkaloids, such as **2** and **3** is similar to the aporphines, although with two successive CO neutral losses. The fragment ion at *m/z* 267.102 corresponds to heterocycle ring opening for both alkaloids. However, for alkaloid **2** this fragment ion indicates an NH_3_ loss, while for alkaloid **3** it indicates a CH_3_NH_2_ loss. The subsequent CH_3_OH elimination yielded the ion at *m/z* 235.075. Consecutive loss of two CO units led to the ion at *m/z* 207.080 with contraction of the isoquinoline A ring, subsequently, the ion at *m/z* 179.086 was formed due to the contraction of ring D. A CH_2_ (14.01 Da) elimination led to the diagnostic ion at *m/z* 165.068, which is the most intense fragment for these alkaloids (**Fig. 4; Table 1; Table S1**). Regarding the fragmentation of the morphinandienone alkaloids, the isomers **8** and **9** could not be accurately differentiated. They show the same fragmentation pattern of aporphines. The fragment ion at *m/z* 297.112 was observed due to the CH_3_NH_2_ neutral loss. Further, the ion at *m/z* 265.086 was formed due to a CH_3_OH loss followed by CO, evidenced by the presence of the ion at *m/z* 237.091 (**Fig. 6; Table 1; Table S1**).

Following the annotations, in the R_t_ range of 2.0-2.33 min, with parent ion at *m/z* 330.170 and parent ion at *m/z* 314.175, the benzylisoquinolines reticuline (**14**, R_t_ 2.07 min) and armapevine (**15**, R_t_ 2.19 min) were respectively annotated. Together with **4** (*m/z* 300.160, R_t_ 1.54 min), these benzylisoquinoline alkaloids show a similar fragmentation. The common tertiary amine loss [M + H - CH_3_NH_2_]^+^ of 31 Da in the high energy spectrum, showed low-intensity fragment ions at *m/z* 269.117, 299.128 and 283.133 to **4**, **14** and **15**, respectively. The observed fragment ions at *m/z* 192.102, *m/z* 175.074, *m/z* 143.049 and *m/z* 137.057 from MS^E^ spectra are diagnostic ions for **14** (**Fig. 5; Table S1**). The ions at *m/z* 175 and 143 are products of subsequent fragmentations of *m/z* 299, first with a loss of the substituted phenyl group (124.06 Da) and then with a common CH_3_OH neutral loss of 32.03 Da with epoxide formation. However, the most intense ions were at *m/z* 192 and *m/z* 137, which are a direct consequence of the fragmentation of the C ring (benzyl), forming either the charged isoquinoline or the broken C charged rings. The *m/z* 192 is also diagnostic for the benzylisoquinoline alkaloid **4**, in which the rings A and B are identical to the **14** (**Fig. 5**). To alkaloid **15**, the respective fragment ion was observed at *m/z* 206.117, which indicates one more methyl group in the isoquinoline ring A. In addition, the ion observed at *m/z* 107.049 is common to **4** and **15** and is related to the charged benzyl fragment and equivalent to the mentioned *m/z* 137, with the difference of 30 Da due to an additional methoxy group attached to benzyl moiety of **14**. (**Fig. 5; Table 1; Table S1**).

In the next, two isomers of noraporphine alkaloids were annotated with parent ion at *m/z* 298.144, as the zenkerine (**16**, R_t_ 2.11 min) and tuduranine (**17**, R_t_ 2.16 min), exhibiting the same fragmentation pattern observed for the previously discussed aporphines. In the MS^E^ high energy spectra were observed for the product ions at *m/z* 281.150, which is related to the isoquinoline opening ring and elimination of NH_3_. Also, the fragment ions at *m/z* 235.076 were attributed to the loss of CH_3_CH_2_OH (46.04 Da), and at *m/z* 207.078, due to the common neutral loss of CO (**Fig. 4; Table 1; Table S1**). In addition, at *m/z* 326.139, the aporphines diospirifoline (**18**, R_t_ 2.13 min), and other two aporphine isomers were annotated with parent ion at *m/z* 342.167 as taliporphine (**19**, R_t_ 2.24 min) and predicentrine (**20**, R_t_ 2.35 min) (**Fig. 4-5; Table 1; Table S1**). Moreover, in the range of 2.4-2.5 min, several aporphine alkaloids were also annotated, as the nuciferine at *m/z* 296.165 (**21,** R_t_ 2.40 min), corydine (**22**, R_t_ 2.41 min) at *m/z* 342.171, domesticine (**23**, R_t_ 2.47 min) at *m/z* 326.139, and dehydrodicentrine (**24**, R_t_ 2.61 min) at *m/z* 340.154. In addition, two noraporphine isomers were also annotated at *m/z* 328.155 as norisocorydine (**25**, R_t_ 2.48 min) and the laurotetanine (**26**, R_t_ 2.52 min). Other three noraporphine were observed, which two are annotated isomers at *m/z* 326.139, as nordicentrine (**27**, R_t_ 2.81 min), and nornantenine (**28**, R_t_ 2.99 min), in addition to the ion at *m/z* 282.149 annotated as nornuciferine (**29**, R_t_ 3.12 min) (**Fig. 4-5; Table 1; Table S1**). Lastly, more six aporphines were observed in the range of 2.5-3.10 min, lirinidine (**30**, R_t_ 2.70 min) at *m/z* 282.149, R_t_ glaucine (**31**, R_t_ 2.90 min) at *m/z* 356.185, and R_t_ roemerine (**32**, R_t_ 3.07 min) at *m/z* 280.133. Also, two aporphine isomers with observed parent ion at *m/z* 340.154 were annotated as nantenine (**33**, R_t_ 2.89 min) and dicentrine (**34**, R_t_ 3.09 min), as well and dehydronuciferine annotated at *m/z* 294.149 (**35**, R_t_ 3.15 min) (**Fig. 4-5; Table 1; Table S1**).

The isomers **29** and **30** could be differentiated by the NH_3_ and CH_3_NH_2_ neutral losses as well. The former generates the fragment ion at *m/z* 265.122 resulting from the characteristic elimination of NH_3_. Then, the product ion at *m/z* 234.104 is formed due to the OCH_3_ loss. The fragment ion at *m/z* 250.099 (C_17_H_14_O_2_^+^) was observed because of the parallel loss of CH_3_. While, for the **30**, the neutral loss of CH_3_NH_2_ has resulted in the ion at *m/z* 251.107, in addition to the consequent neutral loss of CH_3_OH and CO, which has yielded fragment ions at *m/z* 219.081 and *m/z* 191.086, respectively (**Fig. 4; Table 1; Table S1**). The same fragmentation pattern occurs for aporphines **21** and **35**. The fragmentation pathway for the aporphine **32** yielded a fragment ion at *m/z* 249.091 due to the characteristic elimination of CH_3_NH_2_ and CH_3_OH, which formed the fragment ion at *m/z* 219.081. The known consequent neutral loss of CO generated a fragment ion at *m/z* 191.086 (**Fig. 5; Table 1; Table S1**). The same fragmentation pattern was also observed for the other six different aporphines: **13**, **20**, **22**, **24**, **33**, and **34** (**Fig. 5; Table 1; Table S1**).

Moreover, in the range of 3.3-3.9 min, fewer alkaloids were detected. Only one major oxo-noraporphine known as dicentrinone (**36**, R_t_ 3.33 min) was annotated at *m/z* 336.086. The last high-intensity aporphine found in the positive metabolic fingerprint included leucoxylonine (**37**, R_t_ 3.52 min), annotated with precursor ion at *m/z* 400.176. Other high-intensity observed metabolites were phenanthrene alkaloids annotated as stephenanthrine (**38**, R_t_ 3.06 min) at *m/z* 294.149*,* argentinine (**39**, R_t_ 3.20 min) at *m/z* 296.165, and thalictuberine (**40**, R_t_ 3.67 min) at *m/z* 354.169 (**Fig. 4-5; Table 1**). At last, the only member of the tetrahydroprotoberberine alkaloid class was annotated as discretamine (**41**, R_t_ 3.88 min) at *m/z* 328.160 (**Fig. SX;** **Table 1**). The fragmentation pattern of the phenanthrene alkaloids was perceived for **38**, **39** and **40**. The fragment ion at *m/z* 251.107 was observed for **39**, while at *m/z* 249.091 it was attributed to **38**. A subsequent CH_3_OH neutral loss followed by the CO led to the fragment ions at *m/z* 219.081 and *m/z* 191.086, respectively (**Fig. 5-6; Table 1; Table S1**).

**Lignoids**

To the lignoids class, a relatively smaller subset of compounds was successfully annotated with level 2 of confidence, compared to the alkaloid class. Nonetheless, our investigation revealed the presence of numerous lignoids in the *Ocotea* spp. samples. These lignoids were level 3 annotated according to the MSI, mainly in the LC-HRMS QC ESI^+^ metabolic fingerprint by multiple annotation hits from the *Ocotea*DB ((<https://doi.org/10.5281/zenodo.8303382>). More specifically, 89 hits from *Ocotea*DB were observed, and thus, the presence of these compounds substantiates the notion that *Ocotea* species are indeed natural sources of lignoids as well. The limited availability of publicly accessible spectra, combined with the high diversity of possible isomers and complex scaffolds, posed challenges in annotating a larger number of lignoids with level 2 of confidence, where we have annotated specifically one lignan and five neolignans.

We were able to assign the parent ion at *m/z* 355.118 as the sesamin lignan (**42**, R_t_ 3.21 min), exhibiting characteristic fragments. Additionally, we successfully annotated five neolignoids at high-intensity levels, including the bicycloneolignan ocophylol B (**43**, R_t_ 5.38 min) of *m/z* 359.185 with its respective characteristics ions as well (see Supplementary **Table S1**). And the other four neolignans were annotated as eusiderin (**44**, R_t_ 5.46 min) with an ion at *m/z* 387, licarin B (**45**, R_t_ 6.01 min) with parent ion at *m/z* 325.143, licarin A (**46**, R_t_ 6.31 min) with an ion at *m/z* 327.159, and armenin B (**47**, R_t_ 6.35 min) with an ion at *m/z* 373.165. The fragmentation pattern of the last three, which are benzofuran is demonstrated in **Fig. 6** and observed fragments in **Table S1.**

Regarding the **46** a loss of water (18.01 Da) led to a fragment ion at *m/z* 309.147. This fragmentation event is suggestive of the presence of hydroxyl groups. The fragment at *m/z* 295.135 arises from a five-membered ring opening and the concurrent loss of a methoxy group (32.03 Da). This fragmentation pathway suggests the formation of an epoxide and structural rearrangements. Whereas, the fragment ion at *m/z* 203.069 can also be formed after the opening of a five-membered ring, followed by a hydrogen rearrangement that leads to the breakage of phenylpropanoid units. This rearrangement results in a neutral loss of 124.05 Da, which is the diagnostic ion for different benzofurans, including **45** and **47.** Moreover, the **44** has a little different fragmentation pattern because there are no hydroxyl groups as substituents. Instead, there are only methoxy groups, and the five-membered ring is a heterocycle six-membered ring containing 2 oxygens, classified as an oxyneolignan. The characteristic ions are demonstrated together with the fragmentation of **42** and **43** in (Supplementary **Table S2**).

While these findings provide valuable insights into the composition of the investigated lignoids, it is important to acknowledge the limitations imposed by the unavailability of online spectra as well as the absence of deep investigations of fragmentation pathways for most of those compounds previously described in the *Ocotea* genus. Further efforts are warranted to obtain and incorporate additional spectrometric data, facilitating more comprehensive characterization and accurate annotation of lignoid compounds in future studies.

**Flavonoids**

The majority of the high-intensity ionized metabolites in the LC-HRMS QC ESI^+^ metabolic fingerprint were assigned as flavonoids, majorly the glycosylated flavonoids of kaempferol, quercetin and apigenin. Besides the flavonoids, a small phenolic metabolite was annotated as the cyclic polyol quinic acid (**48**, R_t_ 0.56 min) in high-intensity levels at *m/z* 191.054. The non-glycosylated flavonoid backbones of taxifolin (**49**, R_t_ 1.96 min) at *m/z* 303.050, catechin/epicatechin (**50**, R_t_ 2.07 min) at *m/z* 289.071, quercetin (**63**, R_t_ 3.57 min) at *m/z* 301.033*,* apigenin (**64**, R_t_ 2.89 min) at *m/z* 269.044, and kaempferol (**65**, R_t_ 4.10 min) at *m/z* 285.039 were annotated (**Table 1**).

High-intensity annotated glycosylated flavonoids include the class apigenin-based flavonoid patterns. Among them, vitexin (**55**, R_t_ 2.54 min) was annotated at *m/z* 431.097. Compound **55** corresponds to apigenin-8-*C*-glucoside, thus a β-*D*-glucosyl residue attached at position C-8 of the apigenin structure. Another notable glycosylated flavonoid is vitexin-2'-*O*-rhamnoside (**52**, R_t_ 2.42 min) annotated at *m/z* 577.157, a derivative of vitexin with an additional α-*L*-rhamnosyl residue attached at position C-2' of the flavonoid. Additionally, apigenin-7-*O-*rutinoside (**59**, R_t_ 2.74 min) at *m/z* 577.157 was identified as a rutinoside derivative of apigenin with a rutinose moiety attached at position C-7 of the apigenin structure. Lastly, the annotated isomers apigenin 6-*C*-glucoside-8-*C*-arabinoside (schaftoside) or apigenin 8-*C*-glucoside-6-*C*-arabinoside (isoschaftoside) (**60**, R_t_ 2.85 min) at *m/z* 563.142 was observed. The former exhibits a β-*D*-glucosyl residue attached at position C-6 and and α-*L*-arabinosyl residue attached at position C-8 of the apigenin structure, while **60** is the opposite. Several apigenin-based flavonoid characteristic fragments could be observed. For compound **55**, the fragments correspond to the aglycone carbonyl-apigenin backbone at *m/z* 311.052 and a neutral loss of 120.04 Da related to the C-C bond cleavage product, a modified β-*D*-glucosyl residue (C_4_H_8_O_4_) at position C-8. In the case of flavonoid **52**, the fragments also correspond to the carbonyl-apigenin backbone, but with the neutral loss of 266.10 Da related to the additional α-*L*-rhamnosyl residue attached to the oxygen at position 2’ of **55**. To compound **59** the fragments also correspond to the classic apigenin backbone at *m/z* 269.047 with a loss of a deoxygenated rutinose moiety (C_12_H_22_O_9_, 308.11 Da) at position C-7. Finally, the fragment ions observed for compound **60** also repeat the apigenin backbone structure, with losses corresponding to the modified *β*-D-glucosyl residue (120.04 Da) at position C-6, giving a fragment ion at *m/z* 443.09, and the *α*-L-arabinosyl residue (90.03 Da) at position C-8, giving a fragment ion at *m/z* 473.106 (**Fig. 6, Table 1; Table S1**).

A tetrahydroxyflavone compound exhibiting a pattern similar to quercetin, with a parent ion at *m/z* 609.147 (C_21_H_20_O_12_), was detected and annotated as rutin (**53**, R_t_ 2.46 min). Other flavonoids with parent ions at *m/z* 463.087 (C_21_H_20_O_12_) and *m/z* 447.093 (C_21_H_20_O_11_) were annotated as the respective isoquercitrin (**51**, R_t_ 2.40 min) and quercitrin (**56**, R_t_ 2.66 min). These compounds are also tetrahydroxyflavone *O*-glycosides, where the quercetin is substituted by α-*L*-glucosyl and rhamnosyl moieties, respectively, at position C-3 via glycosidic linkage. Another tetrahydroxyflavone, with a parent fragment ion at *m/z* 463.088 (C_21_H_20_O_12_), was identified as quercimeritrin (**54**, R_t_ 2.54 min). Flavonoid **51** corresponds to a quercetin *O*-glucoside, characterized by the presence of a β-*D*-glucosyl residue attached to position C-7 of the quercetin structure. Furthermore, the parent ion at *m/z* 433.077 (C_20_H_18_O_11_), was annotated as reynoutrin (**57**, R_t_ 2.73 min), corresponding to quercetin-3-*O*-xylopyranoside. In the MS^E^ high-energy spectra corresponding to these flavonoids, several characteristic fragments of the quercetin backbone could be observed. For **53**, the fragments correspond to the aglycone quercetin backbone at *m/z* 301.035, with potential losses of the rutinose sugar (308.11 Da) moiety at position 3. The **54** also exhibits fragments corresponding to the quercetin backbone, with a loss of the *β*-*D*-glucosyl residue (162.053 Da) at position C-7. Flavonoid **57** shows fragments corresponding to the quercetin backbone as well, with a loss of the xylopyranosyl residue (132.042 Da) at position C-3. Finally, compounds, **56** and **51** also exhibit fragments corresponding to the quercetin backbone, with a loss of the α-*L*-rhamnosyl residue (146.06 Da) at position C-3 and a glucosyl residue (162.05 Da), respectively (**Fig. 6**, **Table 1; Table S1**).

The kaempferol-based glycosylated flavonoids were also observed in high-intensity levels, including the ion at *m/z* 447.093 that was annotated as astragalin (**58**, R_t_ 2.74 min). Another parent ion at *m/z* 431.098 (C_21_H_20_O_10_) was annotated as afzelin (**61**, R_t_ 3.15 min). Compounds **58** and **61** are glycosyl flavones in which the kaempferol aglycone is attached to *α-L*-glycosyl and rhamnosyl residues, respectively, via a 3-*O*-glycosidic bond. This is evidenced by the presence of the deprotonated aglycone ion at *m/z* 285.039 in the high-energy channel spectra, reflecting the loss of a rhamnose sugar moiety (146.06 Da) for **58** and a glucose moiety (162.05 Da) for **61**. Additionally, kaempferol 3-4''-*p*-coumarylrhamnoside (**62**, R_t_ 3.52 min) and isomers kaempferol 3-(2'',4''-di-(E)-*p*-coumarylrhamnoside) and kaempferol 3-(3'',4''-di-(E)-*p*-coumarylrhamnoside) (**66**, R_t_ 5.56 min) were also annotated. The former is also a glycosyl flavone with a parent ion at *m/z* 577.136 (C_30_H_26_O_12_). The latter with observed parent ion at *m/z* 723.174 (C_39_H_32_O_14_) (**Table 1**). **62** exhibited a single loss of the coumarylrhamnoside moiety (292.10 Da) in high-energy channel spectra, while the loss of a di-coumarylrhamnoside unit (438.132 Da) in the same channels indicated the presence of the isomers **66** (**Fig. 6**, **Table 1; Table S1**). The MS^E^ spectrum further confirmed the aglycone’s identity with characteristic product ions at *m/z* 255.029 and 227.034, matching the literature and corroborating it as a kaempferol derivative.

Moreover**,** fragmentations not included in the manuscript, such as the fragmentation of **1** was proposed based on a 4-hydroxyproline available spectrum in the MoNA database. The difference is 14 Da more is related to the presence of a tertiary amine (*N*-methylated proline). In the MS^E^ high energy scan was observed the main fragment ions at *m/z* 100.0756, which was related to the carboxylic acid neutral loss (46.01 Da). In addition to the ions at *m/z* 82.0651 that are explained by a water loss (18.01 Da), and at *m/z* 72.0808 related to the opened pyrrolidine ring. Besides, the **1** was a hit from the *Ocotea*DB, and it was also putatively identified with high peak areas among the QCs and *Ocotea* sp. extract samples (**Table 1** and **S1**).

**Discussion regarding Supplementary Fig S3-S7.**

The high complexity of our dataset, arising from the diverse matrix of plant extracts and large sample size (n=60), is evident in Supplementary **Fig. S3-S7**. Supplementary **Fig. S3** displays the overlaid chromatograms of features at *m/z* 328.145 within the R_t_ 1.45-2.40 min window from the final aligned feature list generated through MZmine 3 processing. These features represent 7 isomers eluting nearby that could be adequately resolved using the local minimum peak resolver algorithm, although some peaks remained outside the aligned area, suggesting potential for even further refinement. Effective resolution of complex chromatographic regions enables reliable differentiation and semi-quantification of compounds for comparative metabolomics. Furthermore, Supplementary **Figs. S4** and **S5**, showing the final MS-DIAL alignment plots of *m/z* versus R_t_, and Supplementary **Figs. S6** and **S7**, depicting the full MN of both ionization modes, demonstrate the substantial data complexity. These figures illustrate that intricate matrices in large datasets demand sophisticated analytics for robust processing, visualization, and interpretation.

**Supplementary Protocol**

**Step-by-step protocol for the DIA-IntOpenStream pipeline**

*Software download*

- Begin by downloading all necessary software tools for the pipeline. The list of required software includes Waters2mzML, Msconvert, MZmine 3, MS-DIAL, KNIME, WinSPC, and Cytoscape;
- Ensure that each software tool is compatible with your computer's operating system. Check the minimum system requirements on the respective websites of these tools to confirm that your machine has the necessary computational capacity to run them efficiently;
- Follow the detailed instructions provided on each software's website for proper download and installation. These guidelines are found on their respective websites;
- Waters2mzML: <https://github.com/AnP311/Waters2mzML>;
- MSconvert: <https://proteowizard.sourceforge.io/download.html>;
- MZmine 3: <http://mzmine.github.io/download.html>;
- MS-DIAL: <http://prime.psc.riken.jp/compms/msdial/main.html>;
- KNIME: <https://www.knime.com/downloads>;
- WinSPC: <https://winscp.net/eng/download.php>;
- Cytoscape: <https://cytoscape.org/download.html>;
- By carefully downloading and installing these software tools, you will establish a solid foundation for the successful execution of the pipeline;

*Step 1) Sample preparation and data acquisition*

- Begin by preparing your samples according to standard protocols. Collect LC-HRMS/DIA raw data (AIF or MS^E^), ensuring consistency in data acquisition parameters. It could be either profile or centroided data;

*Step 2) Data conversion to .mzML*

- For MS^E^ Waters.RAW data, utilize Waters2mzML for conversion. Place your Waters.RAW files in the “raw_files folder” and run “Waters2mzML-1.2.0.exe”. Decide on centroiding and wait for the conversion completion. The processed .mzML files will be located in the “mzML_files folder”. For more details access the GitHub web page;
- In the case of other types of DIA data (e.g, AIF from Thermo Fischer Orbitrap), conventional conversion on Proteowizard’s msconvert is suitable as well described in MZmine and GNPS documentation, online available at <https://mzmine.github.io/mzmine_documentation/data_conversion.html> and <https://ccms-ucsd.github.io/GNPSDocumentation/fileconversion/>, respectively;

*Step 3) Custom in-house database preparation*

- Begin by selecting a suitable database containing metabolites pertinent to your study. For plant and microbial natural products, databases such as KNApSAcK and the Natural Product Atlas are open-recommended databases. For human metabolites, the Human Metabolome Database (HMDB) is a suitable choice.-;
- Download information related to the metabolites' structures (formats like .mol, .mol2, and .sdf) or simplified text identifiers (such as SMILES, CAS number, InChIKey, and IUPAC name).-;
- Download the KNIME workflow (<https://hub.knime.com/-/spaces/-/~8bZEbbknV8tVptea/current-state/>) for the creation of your *in-house*DB, which is compatible with four common types of chemical input data: .mol, .mol2, .sdf, and .csv (universal table format). As an alternative option, the information can be also added as table input files, which can include SMILES, InChIKey, CAS number, or IUPAC names;
- The outcome of this workflow is a .csv file containing three columns: chemical structure name, calculated molecular formula, and calculated monoisotopic mass. This file will be instrumental for subsequent steps in the pipeline, particularly for enhancing the confidence level of the annotation to level 3;
- This step is crucial for ensuring that your workflow is tailored to the specific metabolites relevant to your research, thereby enhancing the accuracy and relevance of your analysis;

*Step 4) MZmine 3 data processing*

- Import the .mzML converted data into MZmine 3 either by dragging and dropping the files or using the “Import Data Module” under “Raw Data Methods”;
- Follow the basic processing sequence, utilizing separate modules for each step;
- Mass Detection: Identifying ions from the mass spectrometry data;
- ADAP Chromatogram Builder: Constructing chromatograms for detected ions;
- Chromatogram Deconvolution: Resolving overlapping signals in chromatograms;
- C^13^ Isotope Filter: Filtering out carbon-13 isotopes to reduce data complexity;
- Alignment: Using either “Join Aligner” or “RANSAC” depending on dataset size and complexity. The former is the standard, although if your data is highly complex more robust algorithms are sought such as the latter;
- Gap Filling: Filling in missing data points in the aligned feature list;
- Optional steps, such as “Duplicate Feature Filter” and “Feature List Blank Subtraction”, can be applied;
- The final step involves annotation using the *in-house* custom database in the .csv format in the “Annotation” Module. Proper mapping of .csv column names to MZmine features is crucial, as shown in **Figure S19***;*


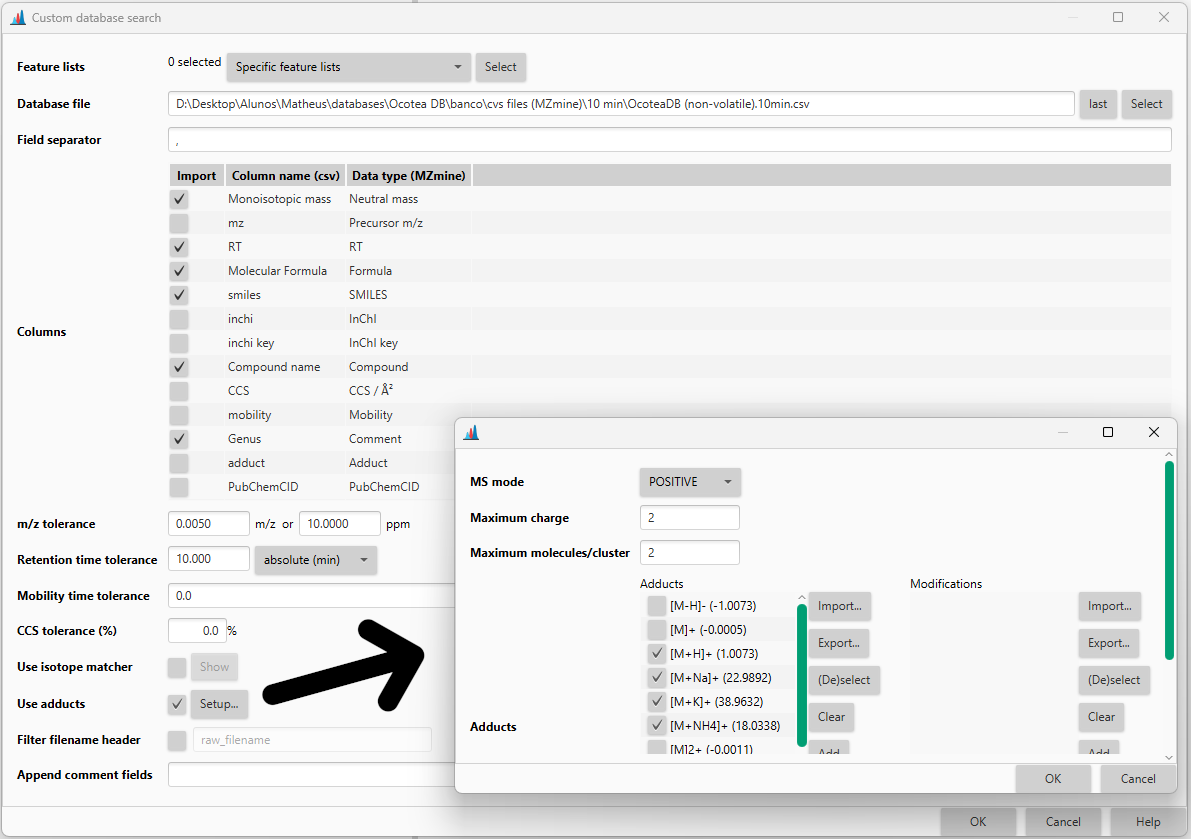


**Fig. S19 | Custom database search settings of the “Annotation” module from MZmine 3.**

- Set the “Use Adducts” option based on your sample and experimental requirements. For instance, in **Fig. S19**, different adducts were selected including [M+H]^+^, [M+Na]^+^, [M+K]^+^, [M+NH4]^+^;
- For detailed explanations of each step and parameter, visit the MZmine Documentation, including tutorial videos <https://mzmine.github.io/mzmine_documentation/index.html>;

*Step 5) Step MS-DIAL data processing*

- Start a new project in MS-DIAL and ensure the directory path is set to where the .mzML files are stored;
- Configure your settings based on your experimental method of LC-MS/DIA, including soft ionization, chromatographic separation, all ions MS method, centroid data, the ionization mode, and metabolomics as target omics);
- Upload the experiment file as described in the MS-DIAL documentation. As an example, we have provided our experiment file at the bottom of this supplementary material;
- Define the sample types (blank, sample, QC) and classes as required;
- Processing in MS-DIAL is an integrated step encompassing peak picking, deconvolution, compound identification, and peak alignment;
- Upload a .txt version of your *in-house* database for custom database annotation during the identification step;
- Export the aligned results to your computer using the “GNPS export” option in MGF or MSP format. In the same page window of alignment parameter settings, the option to filter based on ion abundances from blank samples can also be set (**Figure S20**);


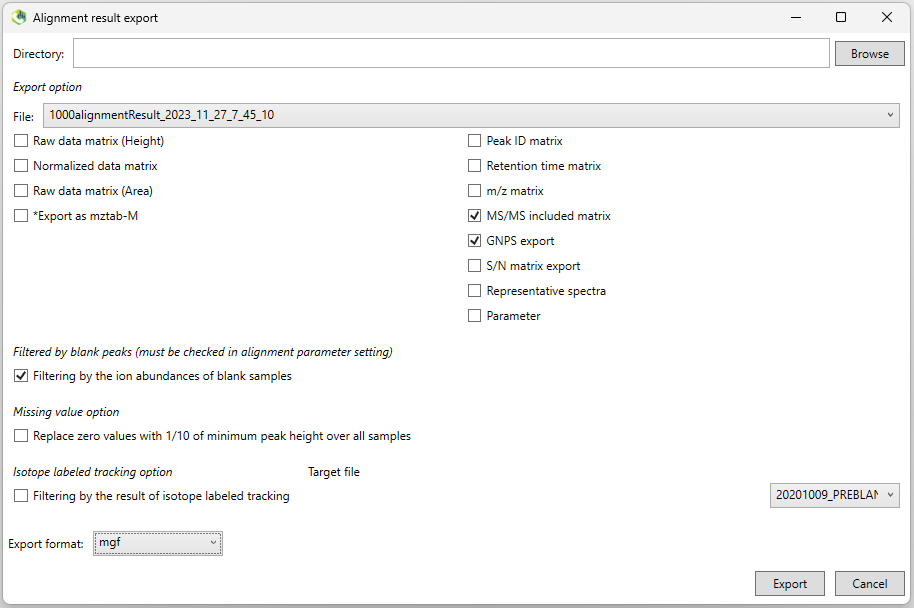


**Fig. S20 | Alignment result export settings from MS-DIAL.**

- Detailed information about processing steps and parameters is described online at <https://mtbinfo-team.github.io/mtbinfo.github.io/MS-DIAL/tutorial>;

*Step 6) Data upload to GNPS server (WinSPC)*

- For uploading files to GNPS, use an FTP client such as WinSPC. If you don't have a GNPS account, create one at <https://gnps.ucsd.edu/ProteoSAFe/user/register.jsp>;
- Connect to GNPS via WinSPC using FTP with no encryption, entering massive.ucsd.edu as the server (port 21), and using your GNPS credentials, as represented in **Figure S21**.


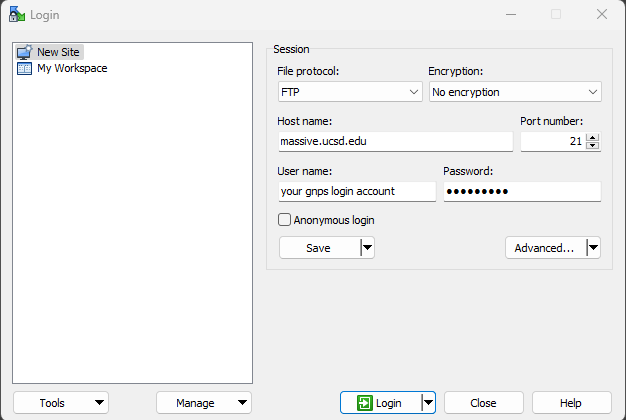


**Fig. S21 | WinSCP login settings to connect to GNPS server.**

- From there, you can upload the files related to the MS-DIAL alignment result, located in the respective directory on your computer (in the left panel) and then drag them over to the GNPS server (in the right panel);
- More information about this process can be found online at <https://ccms-ucsd.github.io/GNPSDocumentation/fileupload/> and <https://winscp.net/eng/docs/guides>;

*Step 7) Feature-based Molecular Networking (FBMN) on GNPS*

- On the GNPS platform homepage (<https://gnps.ucsd.edu/ProteoSAFe/static/gnps-splash.jsp>), after login in, select the option “Feature Networking” in the “Advanced Analysis tools” section;
- Essential for FBMN are two files: the feature table (.txt or .csv format) and the MS/MS spectral file (.mgf format), typically named GNPStable.txt and GNPSmgf.mgf in MS-DIAL exports, respectively;
- Optionally, upload a metadata table via WinSPC for enhanced data analysis. This tab-separated text file can enhance dataset flexibility during data analysis and visualization. It is a text file (Tab-separated) that users must create these file themselves using a text editor (e.g. Microsoft Excel, Notepad++ for Windows, Gedit for Linux, TextWrangler for Mac OS). Using a Metadata table can greatly ease the visualization and analysis of data within Cytoscape before analysis;
- MS/MS libraries can also be uploaded to the GNPS server or selected among the GNPS libraries, to perform an automated annotation;
- In the FBMN interface (**Figure S22**), set parameters such as mass tolerance, cosine similarity, topK, and the number of fragments, as detailed in the main text. In addition, on the FBMN interface, by placing the cursor above some parameter an explanation about it appears;
- Comprehensive guidance on FBMN is online available at <https://lfnothias.github.io/GNPSDocumentation/featurebasedmolecularnetworking/>;


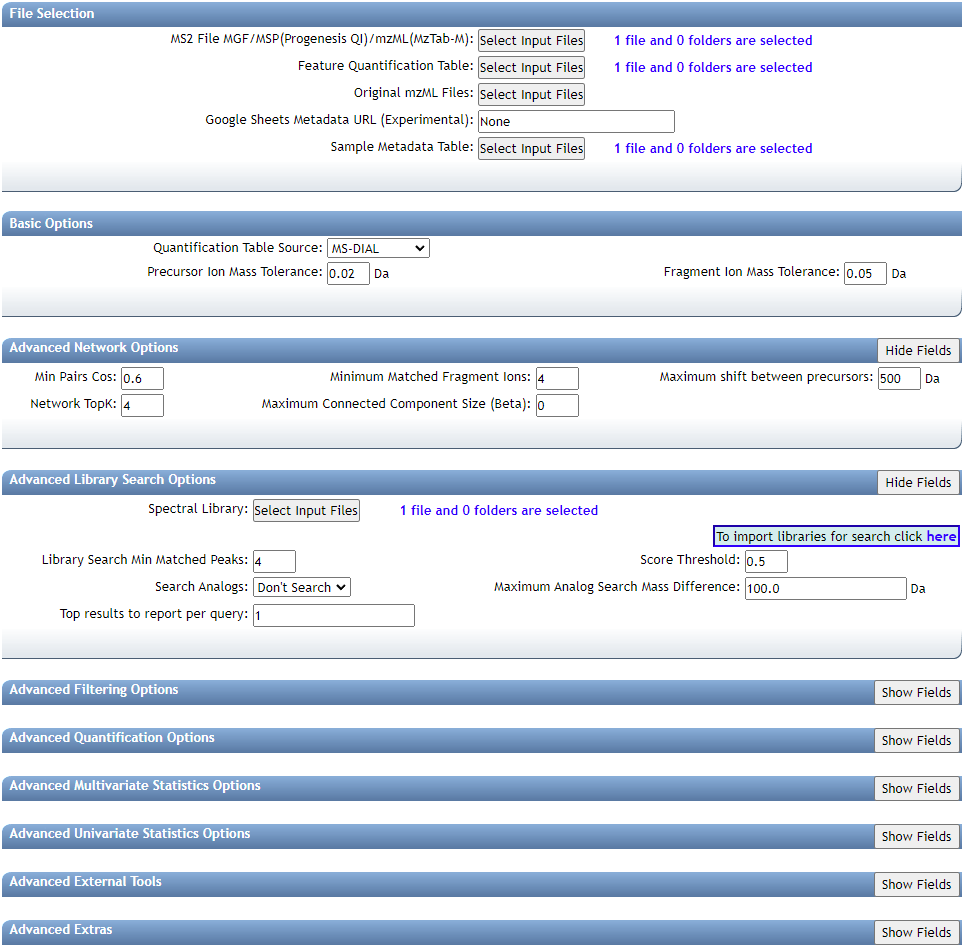


**Fig. S22 | FBMN interface with File selection and other parameter settings.**

*Step 8) Data integration, interpretation and visualization*

*Part 1: MS/MS database search*

- Inspect prominent annotations from the custom *in-house* database (annotation with confidence level 3). Once the biosynthetic-related hits were matched, to enhance the confidence level of these annotations we manually inspect the FBMN and the MS^2^ spectra of these compounds;
- Pseudo MS^2^ spectra from MS-DIAL (automatic) or raw MS^2^ spectra on MZmine 3 (manual) can be compared with MS^2^ spectra available in public databases to search for key fragments. We have applied the public repositories MassBank of North America (<https://mona.fiehnlab.ucdavis.edu/>) and Global Natural Products Social Molecular Networking (<https://gnps.ucsd.edu/ProteoSAFe/libraries.jsp>);
- For compounds without available spectra, propose fragmentation pathways using gas phase fragmentation reactions to bolster confidence in the annotations;

*Part 2: Cytoscape visualization*

- Upon completing FBMN, export the network for visualization in Cytoscape using the “Export/Download Network Files (Download Cytoscape Data)” or “Advanced Views - External Visualization (Direct Cytoscape Preview/Download)”;
- Information about how to customize your network on Cytoscape and improve the visualization can be found online at <https://cytoscape.org/cytoscape-tutorials/contents/index.html#/>;
-
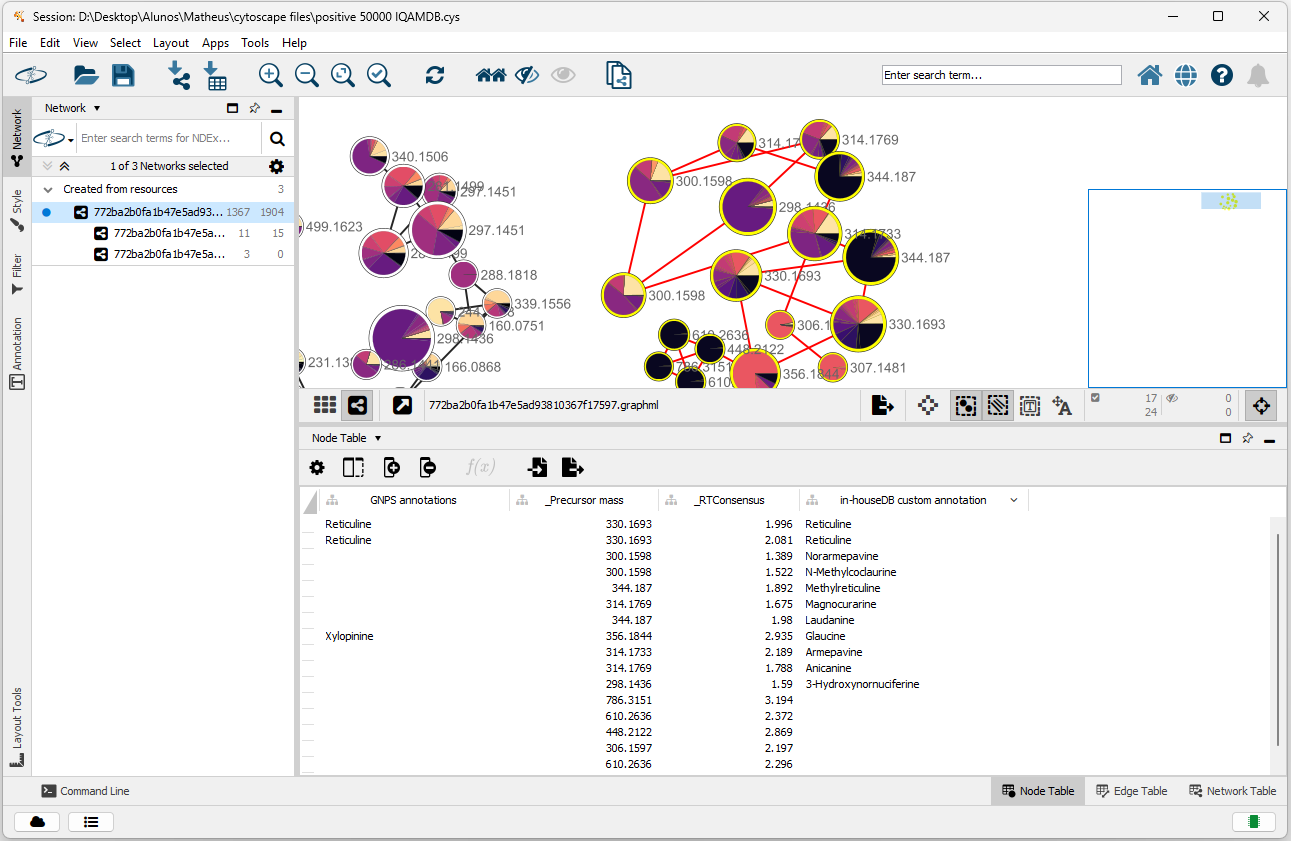
Integrate results from the custom database and GNPS annotations by adding the *in-house* data as a new column in the node table, as shown in **Figure S23**.

**Fig. S23 | Customized molecular network on Cytoscape interface.** In detail, the node table with GNPS automatic annotations for precursor mass and retention time, and *in-house* custom database annotations (monoisotopic mass match) integrated with visual resources.

Concluding remarks: Dive deep into your data! This exploratory phase is crucial in turning raw data into valuable knowledge, potentially guiding future research directions, informing decision-making processes, or contributing to scientific advancements.

**Supplementary material and methods**

**Solvents, plant material and crude extracts preparation (SM-1)**

All solvents used were LC-MS (acetonitrile) and HPLC grade, including hexane, methanol, and ethanol all were acquired from Sigma Aldrich® (St Louis, MO, USA). Formic acid was also supplied by Sigma-Aldrich® (St Louis, MO, USA). Ultrapure water was purified using a Millipore Milli-Q® water purification system (Millipore, Bedford, MA, USA). The liquid nitrogen was purchased from Linde® (Pullach, Munique, Germany). The OUPR herbarium (Federal University of Ouro Preto - UFOP) and CESJ herbarium (Federal University of Juiz de Fora - UFJF) supplied 1-3 leaves of 60 *Ocotea* sp. vouchers to this study. **Table S2** includes information about the geographical location of the plant collection, together with the deposit voucher numbers. The present research was registered on the National System for Governance of Genetic Heritage and Associated Traditional Knowledge (SisGen # A5A8F67). The *Ocotea* species received an identification code (ID) according to their specie scientific plant names, e.g. the *O. odorifera* (OD) (**Table S2)**.

The 60 different *Ocotea* species' leaves vegetal material were weighed (20 mg each) and then pulverized using pistil and liquid nitrogen. Subsequently, 1.7 mL of ethanol: water 7:3 (v/v) was added to the powdered material for extraction. The extracts were placed into a warm ultrasound bath for 15 minutes at 35 °C (170 W, 50 kHz, L100 Schuster) and then centrifuged at 22 °C and 112 rcf (G-force). The collected supernatants (~1.6 mL) were partitioned with hexane (2 x 200 µL) for the removal of fatty material. Next, the extracts were filtered through polytetrafluoroethylene syringe filters (PTFE) of 0.22 μm and dried using a speed vacuum apparatus at 40 °C for 3 h. The extracts were maintained in a freezer at the temperature of -20 °C before UPLC-MS experiments.

**Quality control, data acquisition and analysis (SM-2)**

As part of our internal management system, to fulfil current quality requirement practices for data acquisition and metabolic fingerprint analysis, an analytical quality control (QC) sample was prepared by pooling together 10 μL from each *Ocotea* sample extract at a concentration of 1 mg/mL (600 μL total volume). Thus, the QC consisted of a composite of all *Ocotea* sp. crude extracts prepared at 1 mg/mL concentration. The QC was spread into three vials, kept at 4° in the instrument rack during the whole batch analysis, and injected at the beginning, middle and final chromatographic batch run. To monitor the system's background noise and reproducibility, one replicate (ID: VI), pooled QC, and one blank (water: acetonitrile, 1:1 *v/v*) were randomly injected every 15 injections, covering the start, middle, and end of the chromatographic batch data acquisition. The injection volume of each sample was 5 µL.

Chromatographic separation was achieved using a high-quality C18 (ACQUITY UPLC®HSS T3) reversed-phase column (1.8 μm, 100 x 2.1 mm) at 40 °C. The mobile phase consisted of two components: (A) 1% acidified water with formic acid, and (B) pure acetonitrile. A flow rate of 0.5 mL/min was maintained throughout the analysis. The chromatographic gradient began with an initial composition of 1% B and, was followed by a transition to 15% B at 0.1 min. Further changes in solvent composition occurred at 7.5 min (80% B), 8.5 min (99% B), and 8.6 min (1% B) until 10 min.

The mass spectrometer operated in MS^E^ acquisition mode with alternating high and low-energy scans. The collision energy was set at 3 eV for low-energy scans and 25-40 eV for high-energy scans. Instrument parameters, including cone voltage (40 V), capillary voltage (3.0 kV), cone gas flow (30 L/h), desolvation temperature (300 °C), source temperature (120 °C), and desolvation gas flow (600 L/h), were carefully optimized. High-purity nitrogen was employed for desolvation, collision, and cone gas. The full mass scan range was set from 50 to 1000 *m/z* for functions 1 and 2. To ensure accuracy and reproducibility, a solution of leucine-encephalin was used as a lock mass with *m/z* 554.2622 (ESI^-^) and *m/z* 556.2768 (ESI^+^) for identification. MS data were continuously collected, and lock spray calibration was performed every 10 seconds.

**Waters2mzML development and custom *in-house*DB (SM-3)**

The converter was developed by Anja Miriam Prisching (email: [anja.prisching@uni-oldenburg.de](mailto:anja.prisching@uni-oldenburg.de)). Affiliation: Institute for Chemistry and Biology of the Marine Environment (ICBM), Carl-von-Ossietzky University, Oldenburg, Germany.

URL: <https://github.com/AnP311/Waters2mzML/releases/tag/v1.2.0>.

The proposed KNIME workflow streamlined and facilitated confident level 3 annotation by leveraging chemical structures from diverse sources. We utilize popular formats such as .mol, .mol2, or .sdf files, redrawn from original articles or downloaded from online chemical databases, e.g. ChEMBL (<https://www.ebi.ac.uk/chembl/>) as input files to the KNIME node readers. Our custom *in-house* database, *Ocotea*DB, contains 492 carefully curated chemical structures in .mol format. We have utilized the Nuclei of Bioassays, Ecophysiology and Biosynthesis of Natural Products Database (NuBBE) (<https://nubbe.iq.unesp.br/portal/nubbe-search.html>) on the construction of *Ocotea*DB. NUBBE (<https://nubbe.iq.unesp.br/portal/nubbe-search.html>) stores chemical structures from Brazilian plant species with simple access to downloadable data, allowing the construction of particular plant species, genera, and family databases that can be automatically used in this workflow. For the *Actinomarine*DB, it compromised 6481 NPs related to the genera of *Actinomyces, Streptomyces, Salinospora, Micromonospora, Nocardia, Actinomadura* and *Rhodococcus*, downloaded from the npatlas database (<https://www.npatlas.org/>) in .csv format.

Also, the KNapSAck (<http://www.knapsackfamily.com/knapsack_core/top.php>) or any other NP product database can be used for the assembling of a SMILES or InChIKey table list (.csv or .xlsx). The KNIME output of this workflow is an automatic .csv table containing name, molecular formula and monoisotopic mass. This file can be directly uploaded to MZmine 3 or MS-DIAL. For MS-DIAL, a simple conversion to .txt format is necessary, with the table formatted to include columns for name, monoisotopic mass, and retention time (Metabolite name or ID, *m/z*, and RT, respectively). In both software, the retention time column of the .csv table should be null and ignored (typically set to the full run time for all rows).

**MZmine 3 data processing and analysis (SM-4)**

The following parameters were set of *Ocotea* extracts: mass detection of MS^1^ scans (Scan filters, MS^1^ level=1) using the centroid algorithm (Noise level, 500 and 350 for positive and negative, respectively), feature detection using the ADAP Chromatogram Builder module (Scan filter, MS^1^ level = 1; Minimum consecutive scans, 5; Minimum intensity for consecutive scans, 2 times the noise level; Minimum absolute height, 4 times the noise level; *m/z* tolerance, 0.005 *m/z* or 10 ppm). Chromatogram resolving using Local minimum feature resolver (Dimension, retention time; Chromatographic threshold, 0.85; Minimum search range RT/mobility, 0.04; Minimum absolute height, equal to ADAP; Minimum ratio of peak top/edge, 1.7; Peak duration range, 0.0-1.0 absolute; Minimum *#* of data points, 5). Isotope filtering using 13C isotope filter module (*m/z* tolerance, 0.003 *m/z* or 5.0 ppm; Retention time tolerance, 0.05 absolute; Monotonic shape, true; Maximum charge, 1; Representative isotope, most intense). Alignment was performed using the RANSAC aligner (*m/z* tolerance, 0.007 or 12 ppm; RT tolerance, 0.15 absolute; RT tolerance after correction, 0.1 absolute; Minimum number of data points, 0.2; Threshold value, 0.1) and then gap filling using the Peak finder algorithm (Intensity tolerance, 0.2; *m/z* tolerance, 0.005 *m/z* or 10 ppm; RT tolerance, 0.1 absolute; Minimum data points, 4). Duplicate feature list rows filter (Filter mode, new average; *m/z* tolerance, 0.005 *m/z* or 10 ppm; R_t_ tolerance, 0.07 absolute) and Feature list blank subtraction (Select blanks raw files; Minimum # of detection in blanks, 3; Quantification, area; Ratio type, Average; Fold change increase, true, 3; Keep or remove features below fold change, remove), were applied to achieve the final aligned feature list. All the other existing parameters not mentioned were set to default. The chemical level 3 annotation of the features was performed by applying the Search precursor mass using the local compound database (CSV) search of *Ocotea*DB (*m/z* tolerance: 0.005 *m/z* or 10 ppm; Retention time tolerance: 10 absolute; Use adducts, true, Setup: MS mode, respective to the data; Maximum charge, 1; Maximum molecules/cluster, 2; Adducts, [M+H]^+^, [M+Na]^+^; [M+K]^+^ and [M+NH_4_]^+^ to positive mode; [M-H]^-^, [M+Cl]^-^, [M+Br]^-^ and [M+FA]^-^ to negative mode. The Feature list summaries of individual and aligned features with all parameters are online available (https://zenodo.org/records/10383866).

To optimize the processing parameters, QC samples were used to achieve better deconvolution, resolution and alignment of the features. The first processing with only the QC and blanks was performed looking for the best result and then a second processing with QC, samples and blanks were performed with the same parameters. To validate the data, samples and QC alignment were compared using the mainly known features as markers. Regarding data processing of the publicly available dataset of actinobacterial extracts, some minor alterations were made compared to the *Ocotea* dataset processing. For mass detection, the noise level was set to 200. Chromatogram building and resolving continued in the same configuration. To deisotoping, in addition to ^13^C isotope filter (same configuration of parameters), the Isotope pattern finder module was utilised (Chemical elements, Br, Cl; *m/z* tolerance, 0.004 *m/z* or 7 ppm; maximum charge of isotope *m/z*, 1; search in scans, Single most intense). Alignment and gap filling were kept unaltered. Duplicate feature list rows filter was not necessary in this case due to the lower number of samples. Feature list blank subtraction was used in the same parameter configuration. The Search precursor mass using the local compound database (.csv) was also used to level 3 chemical annotation of the features but through *Actinomarine*DB (*m/z* tolerance: 0.005 *m/z* or 10 ppm; Retention time tolerance: 7.6 absolute; Use adducts, true, Setup: MS mode, respective to the data; Maximum charge, 2; Maximum molecules/cluster, 2; Adducts, [M+H]^+^, [M+2H]^2+^; [2M+H]^+^).

**MS-DIAL data processing and analysis (SM-5)**

For data processing the parameters were set to *Ocotea* extracts as Data collection (MS^1^ and MS^2^ tolerance, 0.01 and 0.05 Da, respectively; Retention time begin/end, 0/10 minutes; MS^1^ and MS/MS mass range begin/end 100/1000 Da; Maximum charged number, 2; Number of threads, 4), Peak detection (Maximum minimum peak height, 1000 and 50000 amplitude; Mass slice width of 0.1 Da for peak detection; Smoothing method, Linear weighted moving average; Smoothing level, 3 scans; Minimum peak width, 5 scans), MS^2^Dec data deconvolution (Sigma window value, 0.5; MS/MS abundance cut-off, 10), Adduct search ([M+H]^+^ and [M-H]^-^, for each mode, respectively) and Alignment (Reference file, B-01p (blank sample); Retention time tolerance, 0.01 minute; MS^1^ tolerance, 0.015; Remove features based on blank information, true; Sample max/blank average, 3 fold change; Gap filling by compulsion, true). Concerning the second data processing (publicly available dataset of actinobacterial extracts), the steps were highly similar to the *Ocotea* dataset, however, some minor alterations were performed to adequate the matrix differences. In Data collection, RT begin/end was set to 0/7 minutes and MS^1^ and MS/MS mass range begin/end to 0/2000 Da. In addition, the option for considering Cl and Br elements was set. For peak detection, an amplitude of 1000 was applied to the Maximum minimum peak height. To adduct search, in addition to [M+H]^+^, [2M+H]^+^ and [M+2H]^2+^ were also sought. For alignment, the option of removing features based on blank information was not set due to several blank chromatogram peaks being present in the same region of the peaks from the actinobacterial samples. The other parameters were not changed. Following data processing the alignment lists were exported to GNPS required file format (Export, Alignment results, GNPS export, true; MS/MS included matrix, true; Filtering by the ion abundances of blank samples, true; Export format, .mgf), with feature quantification table (.txt) and MS/MS spectral summary (.mgf).

**Molecular networking and metabolite annotation analysis (SM-6)**

The set parameters used for generating FBMN were the same for both datasets. Precursor ion mass tolerance of 0.02 Da and fragment ion mass tolerance of 0.05 Da. Advanced network options were set accordingly to our MS^E^ data type (Min pairs Cos, 0.6; Network TopK, 4; Minimum Matched Fragment ions, 4; Maximum Connected Component Size (Beta), 0; Maximum shift between precursors, 500 Da), as well the advanced library search options (Library Search Min Matched Peaks, 4; Search Analogs, Don’t; Top results to report per query, 1; Score Threshold, 0.5; Maximum Analog Search Mass Difference, 100 Da). Other advanced options were not set or set as default. All the GNPS job links are provided together with Zenodo uploaded data.

The initial automated annotation was conducted by searching against GNPS libraries using FBMN. This automated annotation can also be performed within MS-DIAL using metabolomics MSP spectral kits. We utilized the isoquinoline-specific bank IQAMDB from GNPS to the positive mode for the *Ocotea* dataset as various alkaloids of *Ocotea* species belong to this class. Additionally, we used the NIH Natural Products Round 2 libraries in negative ion mode to access the flavonoid content in these samples. As no specific libraries to actinobacterial-derived compounds NP were available on GNPS spectral libraries, we have preconized the main GNPS NP libraries to access the biggest number of metabolites in our samples. In this way, we have applied the following GPNS libraries: Sumner, Respect, NIH Natural Products Library, NIH Natural Products Library Round 2 in positive mode, Birmingham-UHPLC-MS-Pos, Berkeley Lab, GNPS Library, Tuebingen Natural Product Collection and MoNA libraries. It is worth noting that NP class and organism (family or genera) specific libraries are highly beneficial in enhancing the annotation process, as they help avoid irrelevant and non-real hits, and currently, it is not available in GNPS for use for annotation. Regarding the manual spectra checking, structural confirmation through careful inspection of fragmentation patterns by examining the MS^1^ and MS^E^ spectra of the selected metabolite candidate was performed for each metabolite present in **Table 1** using the MZmine 3.

**Supplementary archives (Zenodo)**

Complete additional information regarding the applicability case of *Ocotea* spp. metabolomics data is available in the Zenodo link. The following data is available online: Level 3 annotation in both positive and negative mode; Metadata for GNPS jobs; Level 2 annotation from GNPS IQAMDB and NIH libraries; Feature tables generated from both MZmine 3 and MS-DIAL 4.9. The *Ocotea*DB and the *Actinomarine*DB were generated using the KNIME workflow (<https://zenodo.org/records/10383866>).

**FBMN job links**

Negative mode – NIH Natural Products (50000 amplitude cut-off) <https://gnps.ucsd.edu/ProteoSAFe/status.jsp?task=79db88981e6844a7b9f1315dcebb587a>

Positive mode – IQAMDB (50000 amplitude cut-off)
<https://gnps.ucsd.edu/ProteoSAFe/status.jsp?task=6e3ef1b75b7341c786b1b740e844d970>
Negative mode – NIH Natural Products (1000 amplitude cut-off)

<https://gnps.ucsd.edu/ProteoSAFe/status.jsp?task=92dda20a10d84beab0dc6c5bcb6659a2>
Positive mode – IQAMDB (1000 amplitude cut-off)

<https://gnps.ucsd.edu/ProteoSAFe/status.jsp?task=cb02eaf164ac476195a8255a0af6a682>

Positive mode – Actinobacterial samples – NP GNPS libraries (1000 amplitude cut-off)

<https://gnps.ucsd.edu/ProteoSAFe/status.jsp?task=1148592a538c4a63b55f05a883cf2127>

**MS-DIAL experiment file (.txt format file)**


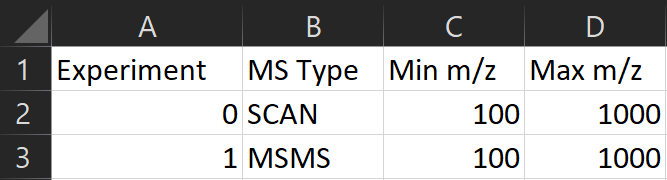

Supplement: Revised_supplemental_material_bbae013 [file revised_supplemental_material_bbae013.docx]
